# Screening of a Microbial Culture Collection: Empowering Selection of Starters for Enhanced Sensory Attributes of Pea-Protein-Based Beverages

**DOI:** 10.1021/acs.jafc.4c02316

**Published:** 2024-07-02

**Authors:** Andrea Spaccasassi, Florian Utz, Andreas Dunkel, Rosa Aragao Börner, Lijuan Ye, Filippo De Franceschi, Biljana Bogicevic, Arne Glabasnia, Thomas Hofmann, Corinna Dawid

**Affiliations:** † Chair of Food Chemistry and Molecular and Sensory Science, TUM School of Life Sciences, Technical University of Munich, Lise-Meitner-Strasse 34, 85354 Freising, Germany; ‡ TUM CREATE, 1 CREATE Way, #10-02 CREATE Tower, Singapore 138602; § Leibniz-Institute for Food Systems Biology, Technical University of Munich, 85354 Freising, Germany; ∥ Nestlé Research, Société des Produits Nestlé S.A., Route du Jorat 57, CH 1000 Lausanne 26, Switzerland; ⊥ Professorship for Functional Phytometabolomics, TUM School of Life Sciences, Technical University of Munich, Lise-Meitner-Strasse 34, 85354 Freising, Germany

**Keywords:** pea protein, plant-based, fermentation, metabolomics, flavor, sensory analysis, sensomics, sensoproteomics

## Abstract

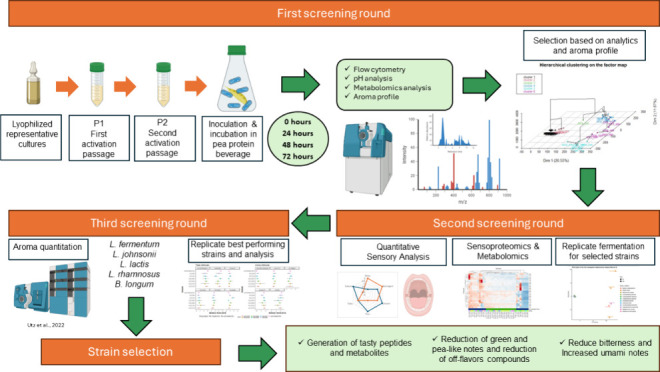

Pea-protein-based ingredients are gaining attention in the food industry due to their nutritional benefits and versatility, but their bitter, astringent, green, and beany off-flavors pose challenges. This study applied fermentation using microbial cultures to enhance the sensory qualities of pea-protein-based beverages. Using UHPLC–TOF–MS analyses along with sensory profile comparisons, microbial species such as *Limosilactobacillus fermentum*, *Lactococcus lactis*, *Lactobacillus johnsonii*, *Lacticaseibacillus rhamnosus*, and *Bifidobacterium longum* were preselected from an entire culture collection and found to be effective in improving the overall flavor impression by reducing bitter off-notes and enhancing aroma profiles. Notably, *L. johnsonii* NCC533 and *L. fermentum* NCC660 exhibited controlled proteolytic activities after 48 h of fermentation, enriching the matrix with taste-active amino acids, nucleotides, and peptides and improving umami and salty flavors while mitigating bitterness. This study has extended traditional volatile analyses, including nonvolatile metabolomic, proteomic, and sensory analyses and offering a detailed view of fermentation-induced biotransformations in pea-protein-based food. The results highlight the importance of combining comprehensive screening approaches and sensoproteomic techniques in developing tastier and more palatable plant-based protein products.

## Introduction

Pea-protein-based ingredients have garnered considerable attention in the food industry due to their nutritional and economic merits, rendering them versatile for diverse food products due to their distinctive technological attributes.^1^ However, the sensory qualities of pea protein, particularly the occurrence of grassy, beany, bitter, and astringent off-flavors, have presented notable challenges for their effective incorporation into various food applications.^2,3^ Recent research has revealed that a broad range of secondary plant metabolites, which bind noncovalently to proteins, are primarily responsible for the undesirable taste and odor characteristics of pea protein isolates and concentrates.^2−6^

**Figure 1:**
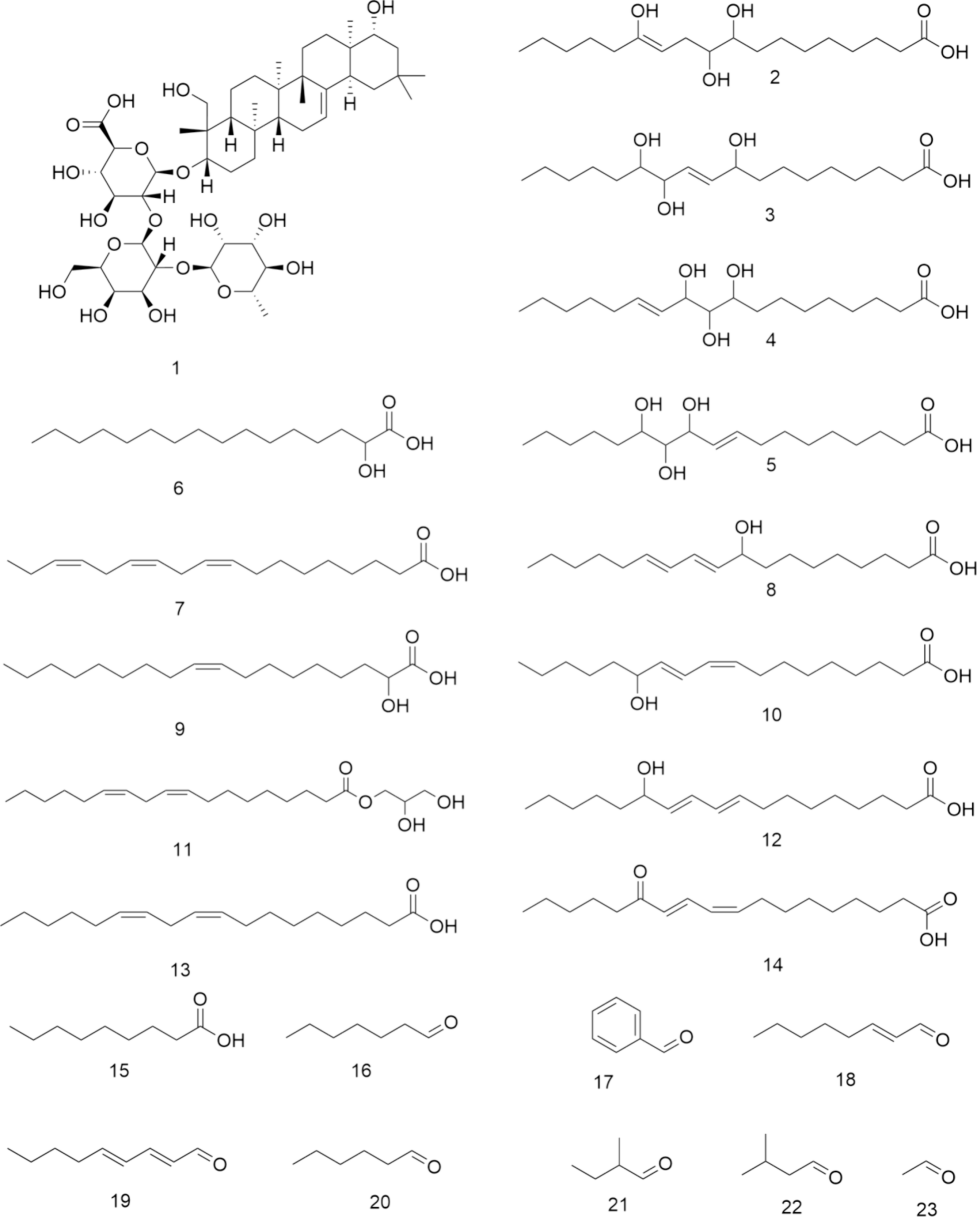
Chemical structures of soyasaponin I (**1**), 9,10,13-trihydroxyoctadec-12-enoic acid (**2**), 9,12,13-trihydroxyoctadec-10-enoic acid (**3**), 9,10,11-trihydroxyoctadec-12-enoic acid (**4**), 11,12,13-trihydroxyoctadec-9-enoic acid (**5**), 2-hydroxypalmitic acid (**6**), α-linolenic acid (**7**), (10*E*,12*E*)-9-hydroxyoctadeca-10,12-dienoic acid (**8**), 2-hydroxyoleic acid (**9**), (9*Z*,11*E*)-13-hydroxyoctadeca-9,11-dienoic acid (**10**), 1-linoleoyl glycerol (**11**), (9*E*,11*E*)-13-hydroxyoctadeca-9,11-dienoic acid (**12**), linoleic acid (**13**), (9*Z*,11*E*)-13-oxooctadeca-9,11-dienoic acid (**14**), nonanoic acid (**15**), heptanal (**16**), benzaldehyde (**17**), (*E*)-2-octenal (**18**), (*E,E*)-2,4-nonaedienal (**19**), hexanal (**20**), 2-methylbutanal (**21**), 3-methylbutanal (**22**), and acetaldehyde (**23**).

Numerous studies have explored the characteristics of volatile organic compounds (VOCs) and aroma compounds in pea-derived enriched ingredients. These studies have focused on changes these compounds undergo during processing as well as their noncovalent protein interactions.^3,6−9^ Notably, Utz et al. (2022) employed a sensomics approach to identify nine odor-active compounds that are crucial in defining the typical “green” and “beany” aromas of pea protein isolates. These compounds include 3-methylbutanal, hexanal, acetaldehyde, (*E,E*)-2,4-nonadienal, (*E*)-2-octenal, benzaldehyde, heptanal, 2-methylbutanal, and nonanoic acid (Figure 1).^3^ These key food odorants are primarily derived from precursors such as unsaturated fatty acids and amino acids via the enzymatic activity of lipoxygenase (LOX) enzymes, auto-oxidation, and amino acid degradation.^10^

Several phytochemicals, especially saponins such as soyasaponin I and DDMP (2,3-dihydro-2,5-dihydroxy-6-methyl-4*H*-pyran-4-one)-saponin)), have previously been associated with a bitter or astringent off-taste in dry peas (*Pisum sativum* L.) and commercial pea protein isolates (PPIs).^11,12^ Human taste threshold determination of pure soyasaponin I followed by quantitation and dose over threshold (DOT) calculation has indicated that the soysaponin I contributes mainly to astringency perception in the tested commercial isolates.^2^ Bitterness molecularization, based on activity-guided fractionation and subsequent calculations of DOT values, has shown that lipids and lipid oxidation products, such as 9,10,13-trihydroxyoctadec-12-enoic acid, 9,12,13-trihydroxyoctadec-10-enoic acid, 9,10,11-trihydroxyoctadec-12-enoic acid, 11,12,13-trihydroxyoctadec-9-enoic acid, (10*E*,12*E*)-9-hydroxyoctadeca-10,12-dienoic acid, (9*Z*,11*E*)-13-hydroxyoctadeca-9,11-dienoic acid, (9*E*,11*E*)-13-hydroxyoctadeca-9,11-dienoic acid, 1-linoleoyl glycerol, α-linolenic acid, 2-hydroxypalmitic acid, 2-hydroxyoleic acid, linoleic acid, (9*Z*,11*E*)-13-oxooctadeca-9,11-dienoic acid, and octacosa-6,9,19,22-tetraen, generated through enzymatic pathways, play pivotal roles in the development of the bitter off-taste of pea protein isolates (Figure 1).^2,4^ Depending on the processing conditions and the specific material under investigation, other sources of bitter stimuli may also play a role in the overall taste impression. Recent investigations have proposed bitter peptides formed during protein isolate processing as additional contributors to the bitter off-flavor in pea protein isolates.^13,14^

As summarized from the perspective of Mittermeier-Kleßinger et al. (2021), identifying and quantifying aroma compounds holds promise for devising strategies to mitigate unfavorable aromas and enhance the overall flavor of pea-protein-based beverages.^15^ Controlled fermentation is a biotechnological avenue for enhancing plant-based foods’ functional, nutritional, and sensory attributes.^16^ Recent studies have shown that fermentation can improve the sensory characteristics of pea-protein-based foods, mainly using various microorganisms such as lactic acid bacteria and *Bacillus* species, which help reduce off-flavors and enhance aromas while also producing health-beneficial bioactive peptides.^17−22^ Despite these advances, much remains to be learned regarding how different starter cultures affect the final product, particularly with regard to nonvolatile, taste-active compounds. While previous research, notably by Harb et al. in 2019, 2020, and 2022, has focused on selecting microbes for fermenting pea protein in beverages,^23−25^ these studies mainly involved volatile compounds and did not extensively examine nonvolatile aspects or correlate flavor to fermentation-induced metabolome changes.

Thus, this study aimed to determine the ideal microbial cultures for fermenting pea-protein-based beverages using a three-step empirical screening process. It employed high-resolution untargeted UHPLC–ToF–MS based metabolomics, targeted LC–MS/MS quantitation for known aroma compounds, sensory analysis, and growth monitoring techniques to check for cell growth and viability. The goal was to identify the most effective starter culture from a large collection to improve the overall flavor of the pea protein beverages. The research examined how fermentation changes these products’ (senso)metabolome. It was further hypothesized that applying cultures directly to the media and conducting thorough metabolomic and sensory analyses would yield detailed information regarding the effects of fermentation on aroma and taste. The methodology intentionally avoided optimizing the fermentation matrix of the pea beverage for the specific nutritional requirements of the cultures. This approach was chosen to evaluate the inherent ability of the bacteria to grow, adapt, and exhibit beneficial flavor effects in a pea-protein-based beverage. Additionally, no additives were included to align with the trend toward “clean labels” in food products, which is particularly important in the sectors focusing on plant-based and sustainable food ingredients.^26^

## Materials and Methods

### Pea Protein Isolate

The pea protein isolate involved in this study was a commercial protein isolate from Roquette (Lestrem, France), presenting a protein content of 84%, a carbohydrate content of 3%, and a fat content of 6%.

### Chemicals

The following chemicals were obtained commercially from Sigma-Aldrich (Steinheim, Germany): de-Man-Rogosa-Sharpe (MRS) medium, propidium iodide, (10*Z*,12*E*)-9-hydroxyoctadeca-10,12-dienoic acid, (9*Z*,11*E*)-13-hydroxyoctadeca-9,11-dienoic acid, linoleic acid, α-linolenic acid, methanol-*d*_4_ (MeOD) ammonium acetate (NH_4_Ac; aqueous solution, 5 mM), formic acid, and acetic acid, l-amino acids (including l-cysteine hydrochloride for media production), nucleotides (Supporting Information (SI), Table S4), nucleosides (SI, Table S4), formic acid, 3-methylbutanal, hexanal, magnesium sulfate, (*E*)-2-octenal, (*E,E*)-2,4-nonadienal, (*E,Z*)-2,6-nonadienal, (*E,E*)-2,4-decadienal, (*E*)-2-undecenal, (*E*)-2-dodecenal, 2-undecanone, heptanoic acid, phenylacetaldehyde, 4-ethylbenzaldehyde, 4-hydroxy-3-methoxybenzaldehyde (vanillin), hexanal-*d*_12_, hexanoic acid-*d*_3_, and phenylacetic acid-^13^C_2_. Heptanal was obtained from Tokyo Chemical Industry (Tokyo, Japan), 3-(methylthio)propanal (methional), hexanoic acid, d-(+)-glucose, d-(−)-fructose, sodium acetate trihydrate, diammonium hydrogen citrate, potassium dihydrogen phosphate, manganese sulfate, and iron(II) sulfate were obtained from Merck KGaA (Darmstadt, Germany), 2,3-octanedione, and (*E,E*)-3,5-octadien-2-one were obtained from aromaLAB (Planegg, Germany), and 3-methylbutanal-*d*_2_ and diacetyl-*d*_6_ were obtained from CDN Isotopes (Pointe-Claire, QC, Canada). (10*E*,12*E*)-9-Hydrox-yoctadeca-10,12-dienoic acid, (9*E*,11*E*)-13-hydroxyoctadeca-9,11-dienoic acid, (9*S*,10*S*,11*R*,12*Z*)-9,10,11-trihydroxyoctadec-12-enoic acid, (9*S*,10*S*,11*E*,13*S*)-9,10,13-trihydroxyoctadec-11-enoic acid, (9*S*,10*E*,12*S*,13*S*)-9,12,13-trihydroxyoctadec-10-enoic acid, (9*Z*,11*E*)-13-oxooctadeca-9,11-dienoic acid, (9*Z*)-12,13-dihydroxyoctadec-9-enoic acid, and 9,10-hydroxyoctadec-12(*Z*)-enoic acid were obtained from Larodan (Larodan AB, Solna, Sweden). Tween 80, tryptone, and HJL medium were obtained from BD (Becton Dickinson, Franklin Lakes, NJ, USA). BHI medium was obtained from Bactor (Bacto laboratories, Mt Pritchard, Australia). 5(6)-CFDA was acquired from C195, ThermoFisher Scientific (Waltham, United States). Dipeptide reference standard solutions were obtained from Bachem (Bubendorf, Switzerland) or peptides and elephants (Hennigsdorf, Germany). All reference standards used for identification and quantitation were obtained in purities >90% and verified using quantitative nuclear magnetic resonance spectroscopy (qNMR) according to a literature-based protocol^27^ and UHPLC–ToF–MS according to the “UHPLC–TOF–MS as a screening platform” methods section.

The water used for chromatography was purified using an Advantage A10 water system (Millipore, Molsheim, France). Bottled water (Evian, Danone; Wiesbaden, Germany) was used for sensory analysis. Methanol and acetonitrile (ACN) used for ultrahigh-performance liquid chromatography–mass spectrometry and for extraction analysis were of LC–MS grade (Honeywell, Seelze, Germany).

### Production of Pea Beverage

A pea protein suspension was prepared by mixing the pea protein isolate with deionized water. The suspension was then homogenized and preheated to 75 °C, immediately followed by UHT treatment. Sterilization of pea beverage by ultrahigh-temperature (UHT) treatment was performed at a 50 L scale. For this treatment, the prewarmed suspension was heated for 4 s to 143 °C at a flow rate of 30 L/h and then efficiently cooled to 4 °C. Finally, the plant protein beverage was aseptically filled into sterile 2 L plastic bottles and stored at 4 °C until use. Before fermentation, the sterilized beverage was manually homogenized. The raw material concentration in the beverage was 10% (m/v), resulting in a protein concentration of 8.4%, a carbohydrates concentration of 0.3%, and a fat concentration of 0.6% in the final beverage.

### Selection of Strains from the Culture Collection

Food grade strains from Nestlé’s Culture Collection (NCC) (Nestlé Research, Lausanne, Switzerland) were selected based on genome diversity to provide a representative sample of the culture collection. The strains employed, as well as their NCC codes, are summarized in SI, Table S1. In summary, during the first round, 69 starter cultures belonging to the families *Lactobacillaceae*, *Staphylococcaceae*, *Bifidobacteriaceae*, *Propionibacteriaceae*, *Streptococcaceae*, *Leuconostocaceae*, *Enterococcaceae*, and *Bacillaceae* were tested.

### Fermentation of Pea Beverages

Three fermentation rounds were conducted in this study. For each fermentation round, one replicate was performed. The first round employed 69 strains from the Nestlé Culture Collection (see SI, Table S1), stored as frozen stocks in 30% glycerol at −80 °C. Activation involved rehydrating lyophilized cultures in specific liquid media for each bacterial group (P1), followed by incubation under specific conditions (detailed in SI, Table S2). A second activation (P2) employed 1% of the P1 culture in new media. Once turbidity appeared in P2 (OD_600 nm_ > 1), 2 mL of this culture was used to inoculate 200 mL of pea beverage were transferred in sterile 500 mL Erlenmeyer flask and incubated on a rotary shaker under specific conditions (130 rpm, 80% humidity, 5 cm shaking diameter). Temperature and oxygen levels were kept as detailed in SI, Table S2. Control samples were uninoculated and treated similarly. Samples were freshly analyzed by flow cytometry to detect microbial metabolic activity. Remaining sample aliquots were frozen then thawed for pH monitoring, metabolomics sample preparation, and sensory analysis.

Fermentation involved 17 preselected strains in the second round and six strains in the third round, based on analytical and sensory results, with incubation times of 0 h (control) and 48 h. Strain selection was based on sensory and untargeted metabolomics analyses. All procedures were conducted under sterile conditions. For the third round only, colony-forming units (CFUs) were measured using the plate serial dilution spotting method on MRS agar and pH values were recorded for all samples to monitor starter culture metabolic activity. Further details of the growth conditions and strain parameters are provided in SI, Table S2.

### Flow Cytometry Analysis

To verify the cellular activity status and cell number of the bacterial strains during the fermentation of pea beverage, single cell analysis of samples from rounds 1 and 2 was performed by flow cytometry. Fermented samples were stained with 5(6)-CFDA mixed isomers at 5 μM final concentration for 10 min at 37 °C. Before acquisition, propidium iodide was added to the samples at a final concentration of 1.5 mM. The acquisition was done with a Beckman Coulter Cytoflex S (Beckman Coulter, Brea, United States) equipped with four lasers: 405, 488, 561, and 638 nm. The fluorescence of CFDA was acquired with the FICT channel (excitation 488, em 525/40), and the fluorescence of PI was acquired with the ECD channel (excitation 561, em 610/20). The combination of PI and CFDA allows the enumeration of dead (PI+) cells, damaged (PI+/CFDA+) cells, and live (CFDA+) cells. CFDA penetrates the membrane of all cells, but only bacteria with an active esterase activity can cleave the CFDA emitting fluorescence. The total number of live, damaged, and dead bacteria (total fluorescent units, TFUs) was normalized to 100% to remove the unstained particles coming from the pea protein matrix. The flow cytometry data were processed with De Novo FCSExpress 6.0.

### Sample Preparation for Untargeted Metabolomics

First, 2.00 g ± 10 mg of each pea beverages were weighed into Precellys 15 mL homogenization tubes filled with 1.4 mm ceramic beads (Bertin Technologies, Montigny-le-Bretonneux, France). Then 5 mL of solvent (80% MeOH, 20% water) was added, and the tubes were cooled overnight at −20 °C. The samples were homogenized using a Precellys evolution homogenizer supplied with a Cryolys cooling module (Bertin Technologies, Montigny-le-Bretonneux, France) according to the following parameters: 6000 rpm, 3 × 30 s, 30 s pause between cycles, temperature maintained at 4 °C using liquid nitrogen. The homogenized samples were centrifuged at 3220 relative centrifugal force (RCF) for 15 min using an Eppendorf centrifuge 5810 R (Eppendorf, Hamburg, Germany) at a stable temperature of 10 °C. Supernatants were filtered with a 0.45 μm Minisart RC 15 membrane filter (Sartorius AG, Gottingen, Germany), placed in a 1.5 mL liquid chromatography vial, and then directly measured by LC–MS analysis. Furthermore, a pooled sample containing an equal amount of each extract was prepared and used as a quality control (QC).

### UHPLC–TOF–MS as a Screening Platform

Samples prepared according to the protocol “Sample Preparation for Untargeted Metabolomics” were analyzed. Analysis was performed using UPLC–TOF–MS on a Sciex TripleTOF 6600 mass spectrometer (Sciex Darmstadt, Germany) and a Shimadzu Nexera X2 system (Shimadzu, Kyoto, Japan) with an IonDrive ion source, operating in both positive and negative ESI modes. After every fifth sample, the instrument’s calibration was verified and corrected using ESI Positive or ESI Negative Calibration Solution and a Calibrant Delivery System (Sciex Darmstadt, Germany). Metabolite separation was performed on two chromatographic columns in distinctive batches. The first method, operated in reverse phase (RP) liquid chromatography, consisted of a 100 mm × 2 mm, 1.7 μm Kinetex C18 column (Phenomenex, Aschaffenburg, Germany) with a gradient of 0.1% formic acid in water (A) and ACN containing 0.1% formic acid (B) at a flow rate of 0.3 mL/min with the following gradient: 0 min, 5% B; 2 min, 5% B; 18 min, 100%B; 21 min, 100% B; 22 min, 5% B; 25 min, 5% B. The second method, operated in hydrophilic interaction liquid chromatography (HILIC), consisted of Acquity BEH amide 100 mm × 2 mm, 1.7 μm column (Waters Corporation, Milford, Unites States) with a gradient of 5 mM NH_4_Ac in H_2_O at pH 3 (A); 5 mM NH_4_Ac, 2% H_2_O in ACN at pH 3 (B) with a gradient of 0 min, 95% B; 2 min, 95% B; 10 min, 50% B; 12 min, 0% B; 15 min, 0% B; 15.5 min, 95% B; 20 min, 95% B. The column oven was set at 40 °C, and TOF–MS scanning was performed from *m*/*z* 50 to *m*/*z* 1500 acquiring for RP runs and from *m*/*z* 50 to *m*/*z* 1000 for HILIC chromatography. Positive and negative polarities were employed for reverse phase (RP) separation, whereas only the negative ESI mode was used for the HILIC separation. MS/MS data were acquired in both data-dependent acquisition (IDA) and data-independent acquisition (SWATH). Ion spray voltage was set at 5500 eV for positive ESI mode and −4500 eV for negative ESI mode; the source temperature was 550 °C, nebulizing gas (0.38 MPa), and heating gas (0.45 MPa). The declustering potential was set to 80 V for all experiments, and the collision energy was 10 V for precursor ion scans and 35 V (including a 20 V collision energy spread) for fragmentation in the individual SWATH windows as well as in the IDA experiments.

In IDA mode, we selected 14 precursor ions per cycle and set switching criteria for the isotope and precursor ions after three occurrences for 5 s to maximize the amount of acquired information. Regarding the SWATH mode, different parameters were used between RP and HILIC separation runs. In RP mode, a series of 23 experiments covering a range from 50 to 1500 Da, overlapping 1 Da (25 ms accumulation time in high-sensitivity mode) were employed. Regarding the HILIC mode, 19 SWATH experiments were used to cover a range from 50 to 1000 Da with 25 ms accumulation time per window acquired in high-sensitivity mode were used. Details of the SWATH windows are reported in SI, Table S3. The sample list was randomized during the run. Then 20 QC samples were run in an initial batch to equilibrate the system according to the matrix. In addition, a QC sample was regularly inserted between samples to provide a reference sample with which to detect analytical variation within the batch as well as a normalization tool as described in the literature.^28^

### Detection and Quantification of Aroma-Active Compounds

Detection, quantitation, and odor activity value (OAV) calculation for the analyzed aroma compounds were performed as described in the literature.^3,29^ Briefly, pea protein beverages (100 mg) were first suspended in a mixture of acetonitrile and water (960 μL, 50:50 v/v) (in technical triplicate for the third-round fermented samples), spiked with the IS solution (20 μL containing 3-methylbutanal-*d*_2_ (22.0 μg/mL), hexanal-*d*_12_ (1.3 μg/mL), decanal-*d*_2_ (23.3 μg/mL), diacetyl-*d*_6_ (19.9 μg/mL), hexanoic acid-*d*_3_ (5.5 μg/mL), phenylacetic acid-^13^C_2_ (2.7 μg/mL), and vanillin-*d*_3_ (1.8 μg/mL) prepared in acetonitrile/water (50:50, v/v) and equilibrated overnight at room temperature under continuous shaking. After at least 20 h, the suspensions were mixed with a solution of 3-nitrophenylhydrazine (NPH, 20 μL, 200 mmol/L) in acetonitrile and water (50:50 v/v) and a solution of *N*-(3-(dimethylamino)-propyl)-*N*′-ethylcarbodiimide hydrochloride (EDC, 20 μL, 120 mmol/L) in acetonitrile and water (50:50, v/v) containing 6% pyridine and derivatized for 30 min at 40 °C. A membrane-filtered (Minisart RC 15, 0.45 μm, Sartorius AG, Göttingen, Germany) aliquot (1 μL) was then analyzed via UHPLC–MS/MS using the same protocol and parameters as described by Utz et al. (2021, 2022).^3,29^ Specifically an Exion LC UHPLC-system (AB Sciex, Darmstadt, Germany) was connected to a QTRAP 6500+ mass spectrometer (AB Sciex, Darmstadt, Germany) and operated in electrospray ionization (ESI) mode (ion spray voltage at +5500 V/–4500 V). Chromatographic separation was achieved on a 100 mm × 2.1 mm, 100 Å Kinetex 1.7 μm XB-C18 column (Phenomenex, Aschaffenburg, Germany) using the following gradient of 0.1% formic acid in water (solvent A) and 0.1% formic acid in acetonitrile (solvent B) with a flow of 0.4 mL/min: 0 min, 27% B; 0.5 min, 27% B; 1 min, 50% B; 6 min, 100% B; 7 min, 100% B; 7.5 min, 27% B, and 9 min, 27% B. The QTRAP 6500+ mass spectrometer was conducted in full-scan mode. As nebulizer (55 psi) and turbo gas (450 °C), zero grade air was used for solvent drying (65 psi). Nitrogen served as curtain (35 psi) and collision gas (1.5 × 10^–5^ Torr), and the quadrupoles were set at unit resolution. Data acquisition and instrumental control was performed with the Analyst 1.6.3 software (AB Sciex, Darmstadt, Germany) and obtained data was evaluated with the MultiQuant software (AB Sciex, Darmstadt, Germany). The quantitative data are expressed in μg/kg of pea beverage.

### Odor Thresholds

Odor thresholds were taken from the Leibniz-LSB@TUM odorant database^30^ or for 2,3-octanedione, and (*E,E*)-3,5-octadien-2-one determined in water from Utz et al., 2022.^3^

### LC-MS Non-quantitative Screening of Taste Actives

The same samples as described in the “sample preparation for untargeted metabolomics” were used for the analysis described in this section. These analytical measurements were performed only for the samples prepared in the third screening round. Reference standards compounds (SI, Table S4) were injected in the same batch with the samples of round 3, accurate *m*/*z* [M – H]^−^, retention time (RT), and the MS/MS fragments were used to identify and map compounds in the samples using the UHPLC–TOF–MS methodology described above in HILIC separation mode and ESI negative mode. Details of the retention time, the TOF-MS *m*/*z* ratio of the precursor in negative mode, and MS/MS fragments are reported in SI, Table S4. Following the identification, their area was extracted from the peak table and used qualitatively to compare the controls against the 48 h fermented materials in round 3. The detected peaks in the samples were then centered, scaled, and plotted to visualize changes between samples. Glutamyl, arginyl, and prolyl dipeptides’ presence and intensity were screened using existing targeted LC-MS/MS methods, and the detected peptide’ peak areas were used to qualitatively compare the samples.^31−33^ Same samples as detailed in the “sample preparation for untargeted metabolomics” section was analyzed using the method’s parameters described in the literature. The results are expressed in the peak areas to compare the intensity of fermented versus unfermented samples.

### Data Analysis and Statistical Evaluation

The UHPLC–TOF–MS data (one replicate for each fermented sample) from HILIC and RP columns were preprocessed using MS-DIAL software (version 4.9.221218).^34^ MS/MS analysis and feature annotation were conducted using MSFINDER software.^35^ In MSDIAL, settings included MS1 and MS2 tolerance at 0.1 Da, a minimum peak height of 1000, a mass slice width of 0.1 Da, linear weighted average smoothing (4 scans), and a minimum peak width (5 scans). Middle QC files were used for retention time alignment, with a higher tolerance for HILIC (0.2 min) compared to RP (0.05 min) and a mass tolerance set at 0.05 Da. Peak table filtering was based on the ion presence in blank samples, with an intensity ratio threshold of 5. Normalization employed LOESS regression for regularly injected QC intensities.

Feature annotation in MSFINDER involved spectral database searches, formula prediction, and *in silico* fragmentation using internal libraries (MassBank, GNPS, and ReSpect). Mass tolerance was 0.05 Da for MS1 and MS2. The formula finder included oxygen, nitrogen, phosphorus, and sulfur atoms. Data processing and visualization were performed in R (version 4.2.3), employing ggplot2, ggpubr, and complexheatmap packages for heatmapping and plotting. Unsupervised multivariate analysis was performed using the R packages FactoMineR, Factoextra. Sciex Software Analyst, PeakView, and multiquant (Sciex, Darmstadt, Germany) were used for data quantification and chromatogram visualization.

Proteomic analysis with MaxQuant (version 1.6.6.0)^36^ was involved *in silico* peptide identification. The UniProt database provided FASTA files for “*Pisum sativum*”. The parameters included an unspecific search due to unpredictable proteinase and peptidase production by bacteria, a minimum peptide length of 3, modifications (oxidation, acetylation), maximum peptide mass of 4600 Da, and Sciex qTOF as MS setting. The deconvoluted evidence file was analyzed in R using similar packages, as described above.

### Sensory Analysis

#### Assessors and Conditions

Sensory evaluations were performed by a diverse group of 14 trained panelists, including seven females and seven males aged between 23 and 35 years, at the Chair of Food Chemistry and Molecular Sensory Science in Freising, Germany. These individuals had been selected based on the absence of known taste disorders and had provided informed consent to participate. Evaluations were conducted in controlled sensory booths maintained at a temperature of 22–25 °C and under yellow lighting to minimize color–flavor interactions.^37^ To focus solely on taste attributes, nose clamps were used to prevent olfactory interference; the clamps were removed for aroma assessments. Panelists were also asked to examine the samples at the end of the evaluation. In addition to Evian water, palate cleansers were provided to clean the mouth between samples.

#### Training Program

Panelists attended weekly meetings to become familiar with the sensory methodologies being used and to be able to evaluate aqueous reference solutions of taste and aroma compounds. For taste attribute familiarization, the training involved evaluating various taste qualities using 5 mL aqueous solutions of distinct taste compounds following procedures described in the literature.^2,38^ To familiarize panelists with aroma attributes, they were introduced to pure aroma compounds (reference standards) diluted in water at 10-fold concentration of the their odor threshold concentration according to the Leibniz-LSB@TUM odorant database.^39^ The reference standards were selected based on the decoded flavor of pea protein and dairy products in literature.^3,29^Table S5 in the SI lists the compounds used for panel training and associated descriptors. Five familiarization sessions (one session per week) were performed prior to the evaluation, and reference standards were made available to the panelists to remind them of the attributes at each sensory session.

#### Qualitative Aroma Descriptive Analysis by Flavor Profile

A qualitative descriptive flavor profile (FP) analysis was used in a consensus session regarding the first round’s samples, as described in the literature;^37^ only the five most experienced panelists who took part in the study from Utz et al., 2022, participated.^3^ This session occurred after aroma training. This method systematically recorded the flavors detected for each 72 h fermented product and the unfermented product after fermentation and their cumulative sensory effect (amplitude). When an attribute was perceived by the panelist, this was rated as 1 (barely recognizable), 2 (slight), 3 (moderate), and 4 (strong). When an attribute was not identified or mentioned, this was filled with 0 (not present). The evaluation of the attributes was not limited to those present in SI, Table S5, but panellists were instructed to add any additional attributes they may perceive in the samples. Each session, five products were evaluated, taking adequate breaks between each evaluation. Upon competition of this process the panel leader derived a consensus profile from the responses of the panel to construct SI, Figure S7. This process was conducted to identify products containing pleasant notes or diminished off-flavors after 72 h of fermentation (first round samples). The criteria for selection were the production of interesting dairy flavors and the reduction of pea protein off-notes.

#### Taste Profile Analysis

An aliquot (5 mL) of the unfermented protein beverage suspended in water was presented to the trained panel. The panel was asked to evaluate bitter, sweet, sour, umami, salty, and astringent taste perceptions on a scale from 0 (not detectable) to 5 (strongly detectable). This procedure allowed the fixation of values for the unfermented reference for the entire study prior to comparative profile analysis performed in the second and third round.

#### Comparative Sensory Profile Analysis

Quantitative sensory profile comparisons were conducted in the second and third rounds. These sessions were conducted with the entire panel. Each session consisted of 3 samples: 1 reference (unfermented sample) and 2 fermented samples. Trained panelists were given a 5 mL aliquot of unfermented reference beverage and an identical aliquot of fermented pea-based beverage for evaluation. The panelists were tasked with rating the intensity of bitter, sweet, sour, umami, salty, and astringent tastes on a scale from 0 (not detectable) to 5 (strongly detectable) relative to the reference unfermented scores, as described in the literature.^2^

For a comparison of aroma profiles, the assessors evaluated the perceived intensities of pea (AR-PEA), green (AR-GREEN), malty (AR-MALTY), fatty (AR-FATTY), and dairy aromas (AR-DAIRY). This assessment was made on a scale ranging from 0 (not detectable) to 3 (strongly noticeable) using the reference unfermented samples as a fixed benchmark. In the first round, panelists were instructed to evaluate the aroma dimensions orthonasally (through the nose) before proceeding with taste evaluation. Conversely, in the second round, the panelists were asked to assess the aroma dimensions retronasally (through the back of the nose), following the taste evaluation.

## Results and Discussion

The objective of this research was to mitigate the presence of undesirable olfactory notes, characterized as “green” or “pea-like”, and to reduce persistent bitterness and astringency in pea-protein-derived beverages. The goal was to improve the palatability for human consumption through fermentation processes. Utilizing the culture collection maintained by Nestlé, we identified food-grade bacterial strains that enhance flavor profiles in pea protein formulations for beverage applications were identified. This selection process was conducted over three experimental phases, employing an integrated approach that combined metabolomic profiling, sensory evaluation, and sensoproteomic analysis.

In the *first phase*, 69 fermented pea-protein-based samples were created using selected strains from Nestlé’s culture collection. This phase involved detailed high resolution untargeted LC–MS analysis to track the influence of these cultures on the metabolome during fermentation, focusing on (senso)metabolites that affected off-flavors in pea protein. Measurements of pH were also taken to monitor fermentation acidification, indicating the metabolic activity. An additional readout of metabolic activity was assessed by flow cytometry to assess the numbers of live, damaged, and dead bacteria. In addition, a qualitative aroma analysis was conducted through a consensus panel evaluation, which selected further strains. These strains, identified as effectively altering the sensometabolome and aroma attributes, were replicated in the second round.

In the *second phase*, the same process as that in the first round was repeated for the 17 preselected strains, including high-resolution untargeted metabolomics analysis, and in addition, quantitative aroma and taste sensory profile analyses were conducted. This process was used to test the initial hypothesis, that those strains enhance the flavor impression of pea-based proteins via fermentation. The top six performing strains then advanced to a final, *third phase*. This round included a combination of untargeted and targeted metabolomics (now totaling three replicates for the elected strains), quantitative aroma and taste comparisons, and aroma quantification using an existing LC–MS/MS_MRM_ method. This final stage determined the most effective strains from the culture collection.

### First Screening Round

In the initial screening round, 69 fermentation starter strains were tested on pea beverages over 72 h in total; these strains are listed in SI, Table S1. Sampling at 0, 24, 48, and 72 h allowed the analysis of pH changes and metabolomics shifts using high-resolution LC–MS. The main goal was to verify growth, cell viability, and select strains that could influence the metabolome of pea beverage. Additionally, at the 72 h time point, aroma profile analysis was conducted to identify bacterial cultures that had significantly affected sensory attributes.

#### pH and Cell Viability Trends

**Figure 2:**
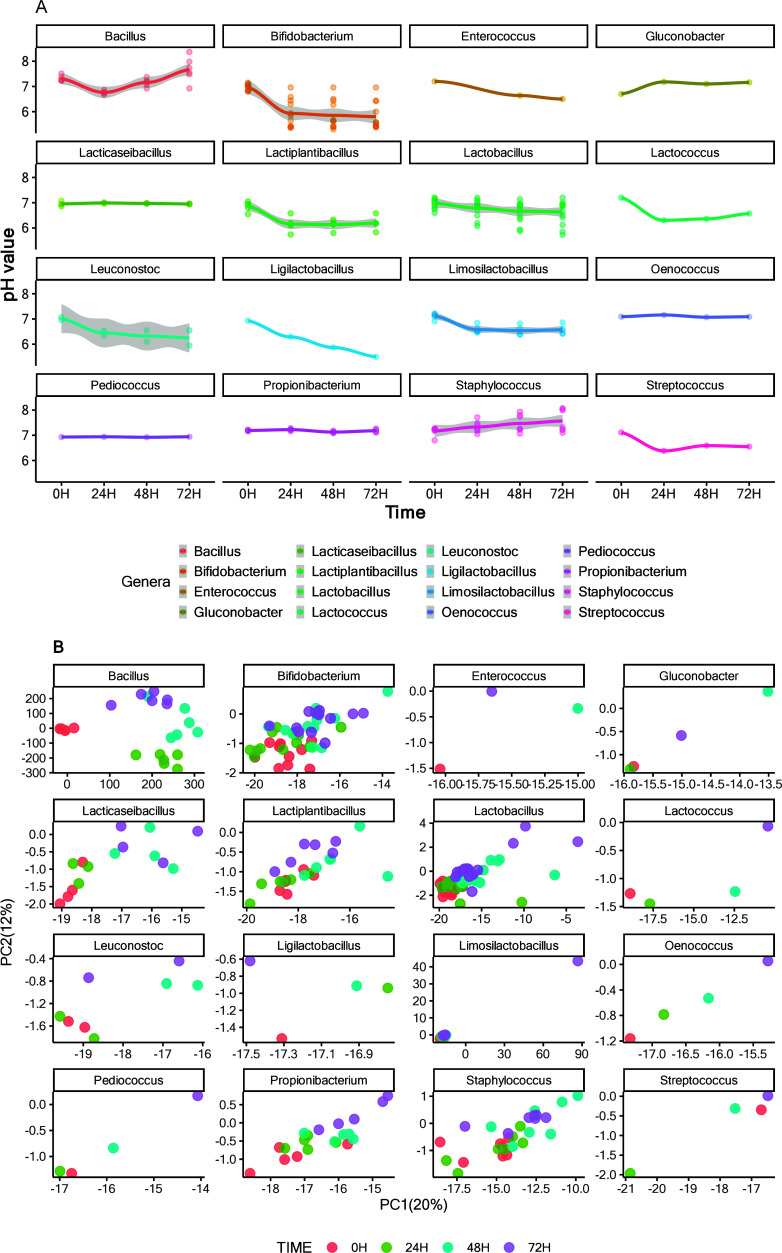
This figure presents a comprehensive analysis of the temporal changes in pH and metabolomic profiles derived from the first screening round. Specifically, (A) illustrates the variation in pH values across a fermentation period exceeding 72 h, with the data stratified by genus. (B) Biplot generated from principal component analysis (PCA) of the untargeted metabolomics data set, with stratification by genus and differentiation by fermentation time.

Data for pH and flow cytometry reflecting metabolic activity and cell viability during fermentation together with the taxonomy of the tested bacteria are detailed in an appendix table (SI, Table S6). Figure 2A illustrates the pH changes throughout fermentation, highlighting the trends for each strain over the 72 h fermentation. Specific genera, such as *Bifidobacterium*, *Lactiplantibacillus*, *Lactococcus*, *Limosilactobacillus*, and *Streptococcus*, primarily acidified within the first 24 h, then stabilized. Others, such as *Ligilactobacillus*, *Lactobacillus*, and *Leuconostoc*, demonstrated continuous pH reduction.

However, *Bacillus* and *Staphylococcus* strains produced an increase in the pH, which was attributed to ammonia production from protein hydrolysis. This rise in pH, or alkalinization, during legume-based fermentation, especially related to *Bacillus* bacteria, is consistent with findings that have been reported in the existing scientific literature.^24^*Propioniibracerium*, *Pediococcus*, *Oenococcus*, and *Lacticaseibacillus* genera did not significantly influence the final pH, probably due to limited growth capabilities in the matrix.

The analysis of bacterial growth trends via flow cytometry analysis in the fermented samples over a 72 h period reveals diverse responses to the fermentation environment. Data is graphically represented in SI, Figures S1 and S2, and detailed in SI, Table S6. Some species, such as *L. fermentum*, *B. bifidum*, *L. citreum*, *L. crustorum*, *L. delbrueckii*, *L. paracasei*, *L. lactis*, *G. oxidans*, *B. bifidum*, *S. condimenti*, *S. thermophilus*, *L. johnsonii*, *L. rhamnosus*, and *L. san franciscensis*, adapt effectively and can proliferate especially in the initial stages of the fermentation process. Remarkably, *Staphylococcus* and *Bacillus* exhibit robust growth, significantly increasing in concentration early on and maintaining high levels throughout the fermentation, suggesting that they thrive well in the pea beverage matrix. *B. breve* and *L. salivarius* showed a peak at a later stage, at 48 h. *L hilgardii*, *B. pseudocantelanum* showed a continuous decline in total bacteria conc, while *L. reuteri* and *O. oeni*, in contrast, showed constant increase.

From these data, it seems that the growth and/or the survival of bacterial strains in a fermentation matrix can occur independently of significant pH changes. This can be attributed to the metabolic diversity of the bacteria. This suggests that while some strains are more resilient or adapted to the pea beverage environment, they can utilize available nutrients and survive without markedly altering the pH. Particularly for *Propioniibracerium*, *Pediococcus*, *Oenococcus*, and *Lacticaseibacillus* does not have change of pH but maintains viable cells through the fermentation.

#### Untargeted Metabolomics

Moreover, metabolomic profiles were analyzed using UHPLC–TOF–MS. The results of the untargeted metabolomics analysis involved processing 17 830 features through principal component analysis (PCA), with data obtained from RP separation in both positive and negative ionization modes. The biplot in Figure 2B, categorized by genera, shows that the *Bacillus* genus produced the most significant metabolomic shifts, indicating strong metabolic activity and changes during fermentation. This effect was further confirmed by hierarchical clustering in SI, Figure S3, in which the unique profile of *Bacillus* forms three distinct groups (24, 48, and 72 h).

According to the data, key components affected by these genera included bitter-tasting fatty acids characteristic of pea protein, their oxidation products, and peptides from *Pisum sativum* storage protein, as highlighted in detail in the following section. The mass spectral and elution details of the features discussed in the following description are presented in the SI, Table S7; which further reports the feature identifiers, precursor types, average mass-to-charge ratios, retention times on the C18 column, MS/MS fragmentation patterns, errors in [mDa] and MSFINDER *in silico*, and database annotation structure identifiers.

The *Bacillus* genus was found to longitudinally affect the intensity of features related to the bitter tasting monohydroxy octadecadienoic acids (**9** and **10**, and **12**, Figure 1). In addition, *B. subtilis* affected the levels of trihydroxy octadecenoic acid features 930N, 931N, and 925N, with an average *m*/*z* 329.2, formula C_18_H_34_O_5_, with retention times of 9.2, 10.1, and 9.8 min, respectively, belonging to the class of bitter-active trihydroxy octadecenoic acids (**3**–**5**, Figure 1) and features 804N and 805N, with an average *m*/*z* 313.2 [M – H]^−^ and retention times of 11.6 and 11.7 min, respectively, formula C_18_H_34_O_4_, matching dihydroxy octadecenoic acid (9,10-DHOME or 12,13-DHOME, taste impression not reported in literature, but bitterness is assumed).

Example line plots indicating the time-related change in the logarithmically transformed peak area of features 585, 685, and 930N extracted from the alignment file obtained are visualized in SI, Figure S4. *L. fermentum* NCC3059 impacted the level of monohydroxy octadecadienoic acid feature 685 as well.

Peptide production from the hydrolysis of *Pisum sativum* storage protein mainly characterized the variance related to data obtained in positive mode acquisition. Figure S6 in SI summarizes the deconvoluted peptidome analysis of fermented products after 72 h. The *Bacillus* genus produced many peptides detected during the 72 h fermentation time compared to those in unfermented products. *Bacillus subtilis* strains NCC4032, NCC2982, NCC2976, NCC2971, and NCC2957, and *Limosilactobacillus fermentum* strain NCC3059 were found to be strong peptide producers (SI, Figure S6). In flow cytometry data, we observed a higher number of small particles in the matrix fermented with these genera. This observation is potentially connected to the strong proteolytic activity and consequent degradation of the pea granules (observational data).

#### AP Sensory Analysis

In addition to alterations in pH, comprehensive analyses through metabolomics and proteomics, particularly in relation to the orthonasal properties of the fermentates, hold significant relevance. Consequently, the aroma profiles of the 69 ferments at 72 h were subjected to examination. The findings derived from the AP consensus panel evaluation are concisely presented in Table 1 and visually represented in Figure S7 in the SI. Several strains emerged as promising aroma improvers for the next evaluation phase. *Lactobacillus rhamnosus* (NCC525 and NCC4007) enhanced fatty, flowery, honey, and yogurt aromas. *Lactobacillus paracasei* (NCC2511) increased cheesy notes. *Bifidobacterium longum* (NCC283 and NCC2705) added fatty cheese/mozzarella aromas and reduced pea-like notes. *Lactobacillus johnsonii* (NCC533) lessened pea-like and grassy notes while enhancing dairy flavors. *Lactobacillus fermentum* (NCC660 and NCC3059) reduced green and pea-like notes. *Staphylococcus carnosus* (NCC 1061) decreased green and pea-like notes, adding leather notes. *Lactococcus lactis* subsp. *lactis* (NCC2378) lowered pea-like notes and introduced new dairy aroma impressions. *Streptococcus thermophilus* (NCC 2795) contributed butter, caramel, malt, and fruity notes. *Lactobacillus plantarum* (NCC1240) reduced the green notes.

**Table 1:** Summary of the Aroma Profile Analysis Obtained from the Consensus Panel at the End of Round 1^a^

strain	results summary from the aroma profile analysis
*L. rhamnosus* NCC525	enhanced fatty, flowery/honey, yogurt aromas
*L. rhamnosus* NCC4007	enhanced fatty, flowery/honey, yogurt aromas
*L. paracasei* NCC2511	enhanced cheesy notes (“cheese crust”)
*B. longum* NCC283	fatty cheese/mozzarella, less pea-like
*B. longum* NCC2705	fatty cheese/mozzarella, less pea-like
*L. johnsonii* NCC533	lowered pea-like, less grassy, enhanced dairy
*L. fermentum* NCC660 and NCC3059	reduced green and pea-like notes
*S. carnosus* NCC 1061	less green and pea-like, new leather notes
*L. lactis* subsp. *lactis* NCC2378	decreased pea-like, new dairy aromas
*S. thermophilus* NCC 2795	butter, caramel-like, malty, fruity notes
*L. plantarum* NCC1240	less green final product
*B. subtilis*	able to reduce tri- and dihydroxy fatty acids, high proteolytic activity

a The table summarizes the impact of different strains on flavor profiles in fermented pea protein beverages.

#### Summary of the First Screening Round

The preliminary analytical screening identified 17 strains for further investigation based on their significant capacity to degrade bitter fatty acids in pea protein. Strains selected during the consensus panel session were additionally incorporated into the second screening phase, as delineated in Table 1. Subsequent investigations explored their effect on the final sensory profiles of the product in more detail.

### Second Screening Round

**Figure 3:**
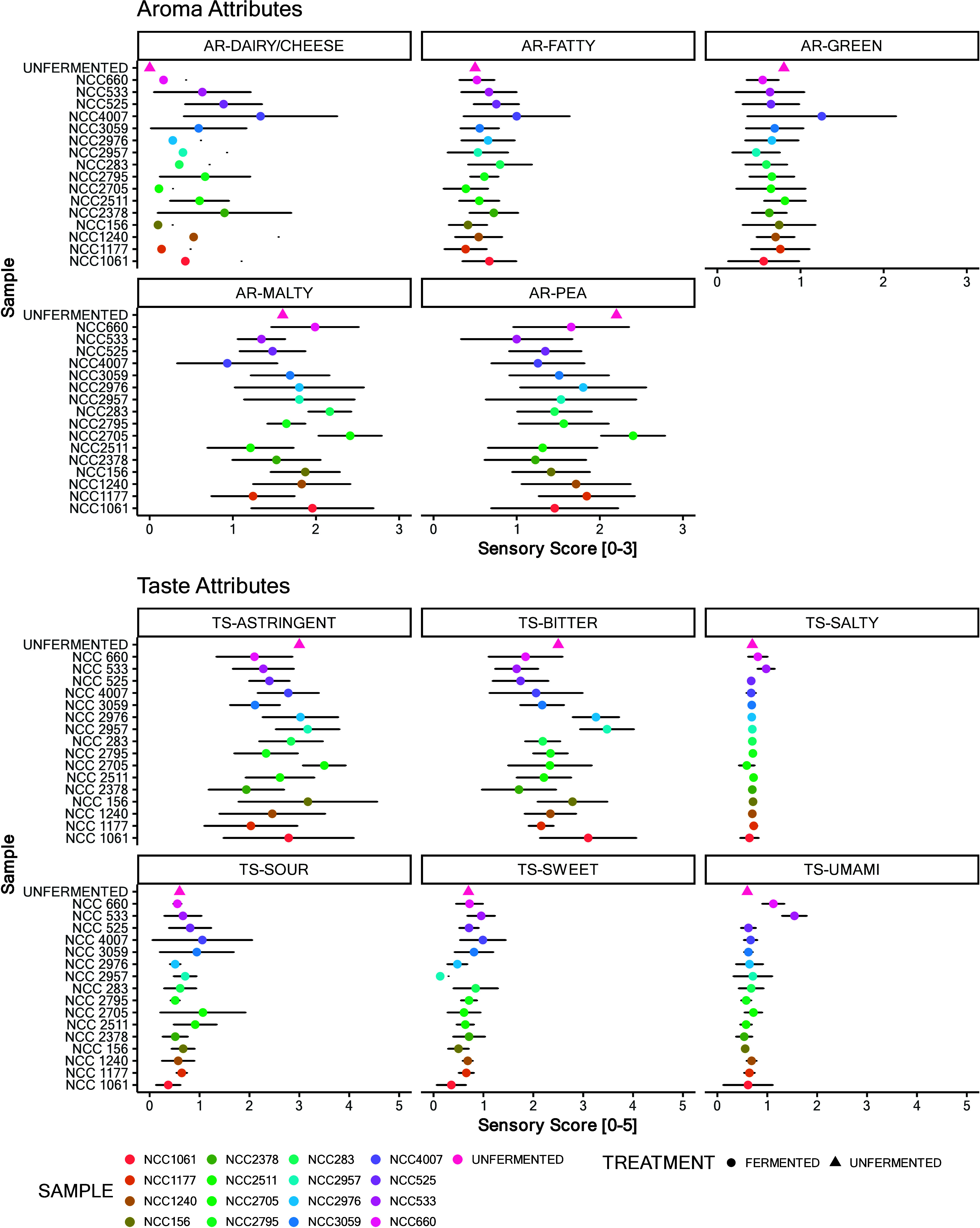
Sensory profile analysis from the second screening round depicts the outcomes of the sensory profile analysis from the second screening round, focusing on samples subjected to a 48 h incubation period. The reference point for comparison is the unfermented product, positioned at the top of the *y*-axis. This figure illustrates the mean sensory scores of the unfermented baseline, against which the sensory attributes of the 48 h fermented samples were evaluated by panelists. Both the mean values and standard deviation for these assessments are depicted.

In this phase of the study, fermentation starter strains selected from the culture collection were tested for their impact on pea beverages over 48 h, as detailed in Table 1 and SI, Table S9. also reports the flow cytometry data for the 48 h fermented samples from the second round. This data indicated the presence of viable cells in the samples after 48 h incubation time. The primary aim of this phase was to evaluate their influence on the sensory profiles quantitatively and confirm the metabolome changes observed in the earlier round of pea beverages. Using comprehensive quantitative sensory analysis, we compared the attribute scores of these fermented beverages to their unfermented counterparts regarding both taste and aroma, as illustrated in Figure 3 and detailed in SI, Tables S10 and S11.

#### Taste Evaluation

Significant variations in taste were observed, particularly in the reduction of bitterness and astringency by preselected lactic acid bacteria (notably strains *L. fermentum* NCC660, *L. johnsonii* NCC533, and *L. lactis* subsp. *lactis* NCC2378, and *L. rhamnosus* NCC 4007). Conversely, *B. subtilis* and *B. amyloliquefaciens* as well as *S. carnosus* strains increased bitterness and altered sweetness (the latter effect likely due to taste–taste interactions).^40^ Enhanced umami taste in fermented samples was particularly noted in products fermented with *L. johnsonii* NCC533 and *L. fermentum* NCC660. This increase in umami flavor is an important aspect of the sensory enhancement of the final fermented product, as it often leads to greater palatability.^41^

In terms of astringency, the changes (general reduction upon fermentation by *L. fermentum* NCC660, *L. johnsonii* NCC533, *L. lactis* subsp. *lactis* NCC2378, *L. fermentum* NCC 3059, and *L. rhamnosus* NCC 4007) depicted in Figure 3 are most likely related to the impact of fermentation on gel formation, which occurred during the pH shift, as no changes in the areas of the saponin-related features were observed in the metabolomics analysis (SI, Figure S5). Formation of microgels has been connected to improvement of the lubricating properties of food, which may result in decreased astringency and sandiness perception.^42^ The degradation and destabilization of pea proteins (especially albumin) may also play a role in astringency perception.^43^

#### Aroma Evaluation

As both odor and taste impressions are crucial for the overall flavor impression, aroma analysis was performed as well. Figure 3 indicates average sensory scores for each aroma attribute perceived orthonasally. Relevant changes in the intensity of the aroma attributes occurred during fermentation. The AR dairy/cheese attribute increased in perceived intensity levels compared to the reference value in products fermented with all the evaluated bacteria except for strains of the species *B. subtilis*, *B. amyloliquefaciens*, and *P. freuderehcihii*.

Generation of dairy aroma notes is expected during fermentation, especially with lactic acid bacteria or dairy bacteria, such as *S. thermophilus*, *L. lactis*, *L. rhamnosus*, *L. plantarum*, *L. paracasei*, *L. fermentum*, and *B. longum*. Regarding AR-green and AR-fatty attributes, a relevant difference could be seen only for *L. rhamnosus*. This bacterium seems to have impacted the products’ overall aroma profiles the most. In addition, the AR-pea attribute presented major differences; most of the strains reduced its perceived intensity compared to the unfermented reference products. The overall decrease in the pea-like aroma is consistent with other reports in the literature.^44−49^

An in-depth investigation of the mechanism of aroma improvement has been published elsewhere.^10^ Briefly, off-flavor reduction via fermentation is primarily connected to the action of alcohol dehydrogenase (ADH) and aldehyde dehydrogenase (ALDH). These enzymes convert aldehydes and ketones into alcohols and carboxylic acids, which contribute less significantly to off-flavors. *Limosilactobacillus fermentum*, *Lactiplantibacillus plantarum*, and *Streptococcus thermophilus* are regarded as capable of carrying out this metabolic activity.^10^

Moreover, *B. subtilis*, *S. carnosus*, and *B. amyloliquefaciens* worsened the overall sensory properties of the pea beverage. This result suggests that excessive fermentation with these bacteria produced undesirable off-flavors. This effect was most likely due to increased bitterness and the presence of sensory attributes (spontaneously added by panelists during aroma evaluation) such as “rancid” “fecal,” and “leather-like,” which are considered unwanted in such a food product.

#### Metabolomic Screening

**Figure 4:**
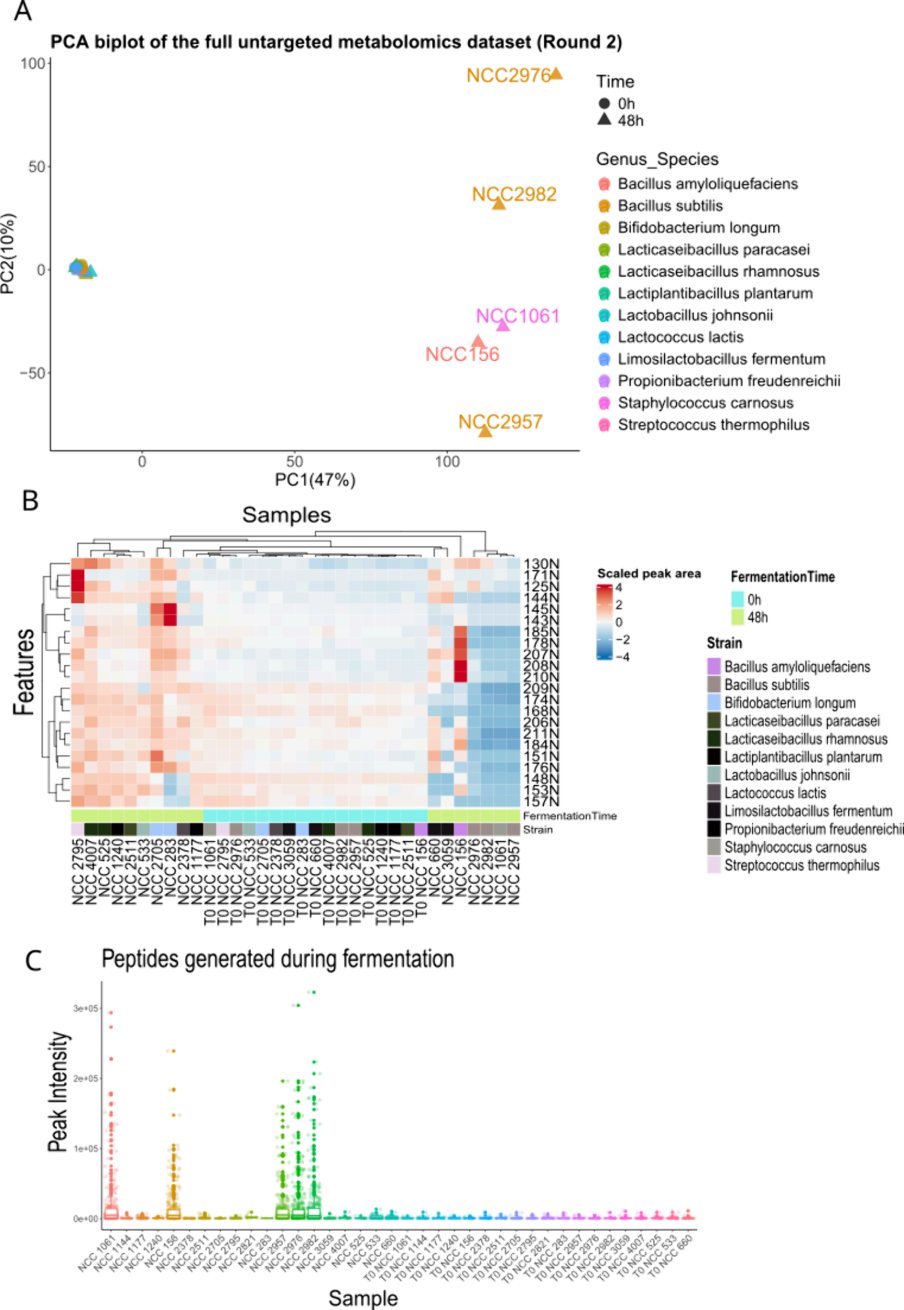
General summary of the analytical results of round 2. (A) Biplot obtained from a principal component analysis of the untargeted data. Data included peak areas from the alignment table of the two reverse-phase (RP) runs in positive and negative ionization modes and the hydrophilic interaction liquid chromatography (HILIC) run in negative ionization mode. (B) Heatmap that shows the peak areas of features downregulated during fermentation and belonging to the molecular cluster of C18 fatty acids and their oxidation products. (C) Point plot that summarizes the identified peptides and their intensities as a plot. The boxplot indicates the average intensity distribution for each sample, and the points indicate the individual peptides detected in that sample and their respective peak areas (intensity).

Untargeted metabolomic analyses*e*s were performed again to confirm the results from round one as biological replicates. All findings were consistent with observations from the initial round and revealed that *Bacillus* strains as well as *Staphylococcus carnosus* NCC1061 significantly altered the beverage metabolomes, as evidenced by the dramatic decrease in the signature fatty acids of the pea protein and the increased presence of peptides, indicating strong proteolytic activity (Figure 4A,B). Details of the features with ontology related to the fatty acids and their oxidation products depicted in Figure 4B are presented in SI, Table S12.

Proteomic analysis revealed diverse proteolysis patterns among the species. Strains like *L. fermentum* NCC660 and *L. johnsonii* NCC533 were associated with reduced bitterness and enhanced umami flavors. In contrast, *Bacillus* and *Staphylococcus* strains were linked to increased bitterness, likely due to excessive protein hydrolysis. This was supported by peptidome data (Figure 4C and SI, Figures S8 and S9) and panelists’ feedback, which noted a sharp, immediate bitter taste in fermented samples, as opposed to the enduring bitterness of unfermented pea protein.

#### Summary of the Second Round

In summary, certain bacteria such as *L. johnsonii*, *L. fermentum*, *L. rhamnosus*, and *L. lactis* supsp. *lactis*, and *B. longum* did not significantly alter the bitter and astringent-tasting detectable plant metabolites of pea protein, they nevertheless beneficially affected sensory properties by reducing bitterness, enhancing umami flavor (particularly *L. johnsonii*, *L. fermentum*), and decreasing aroma off-notes, as depicted in Figure 3. The six preselected strains were further examined in the next fermentation round to monitor their impact on the metabolome and replicate the sensory profile comparison.

### Third Screening Round

In this phase, our investigation continued with the six strains identified in the initial screening rounds, aiming to validate their effects on the sensory profiles of pea-protein-based beverages. Building on hypotheses developed from earlier findings, we conducted a deeper analysis of these selected starters, focusing on texture, aroma quantification, bacterial growth, taste, and retronasal aroma evaluation over a 48 h fermentation period. The goal was to replicate the positive outcomes observed with the top-performing strains and investigate the mechanisms responsible for flavor improvement.

#### pH, CFU Count and Texture Shifts

Details regarding the strains, pH values, and cell counts are given in Table 2. A significant pH decrease was noted in samples fermented with strains such as *L. johnsonii* NCC533 and *L. lactis* subsp. *lactis* NCC2378, as detailed in Table 2 and previously described in phase 1. This acidification affected the texture of the beverages, leading to clotted material in some cases, likely due to changes in protein solubility and surface properties.^21^ The colony-forming unit (CFU) count, also reported in Table 2, varied across the samples, with some exhibiting a decrease over the fermentation time. This effect suggests differential bacterial adaptability to the pea protein matrix over the 48 h incubation time. *L. fermentum* NCC3059 and *L. rhamnosus* NCC4007 did not exhibit growth when comparing the 0 h control vs the 48 h fermented sample. As far as *L. fermentum* NCC3059, according to the flow cytometry data acquired in the first round, most of the growth seems to happen in the first 24 h and possibly leading to a decrease in viable cells and increase in dead/damage cells from 24 to 48 h (SI, Figure S1). This is most likely due to limited nutrient availability, which limits growth beyond the 24 h time point.

**Table 2:** Summary of the Species and Strains Employed during the Third Round^a^

NCC identifier	species	CFU/mL, 0 h	CFU/mL, 48 h	pH	observation after fermentation
NCC2705	*Bifidobacterium longum*	8.80 × 10^8^	1.20 × 10^8^	5.35	hard clotted
NCC2378	*Lactococcus lactis* subsp. *lactis*	1.55 × 10^8^	1.50 × 10^9^	5.69	creamy clotted
NCC3059	*Lactobacillus fermentum*	6.60 × 10^8^	1.50 × 10^7^	6.52	liquid
NCC660	*Lactobacillus fermentum*	1.40 × 10^8^	5.00 × 10^7^	6.08	clotted
NCC4007	*Lactobacillus rhamnosus*	8.60 × 10^8^	2.00 × 10^7^	6.93	liquid
NCC533	*Lactobacillus johnsonii*	1.10 × 10^9^	1.50 × 10^8^	5.80	hard clotted

a Final pH, cell counts before and after fermentation, and observationafter fermentation are also included.

#### Aroma and Taste Evaluation

**Figure 5:**
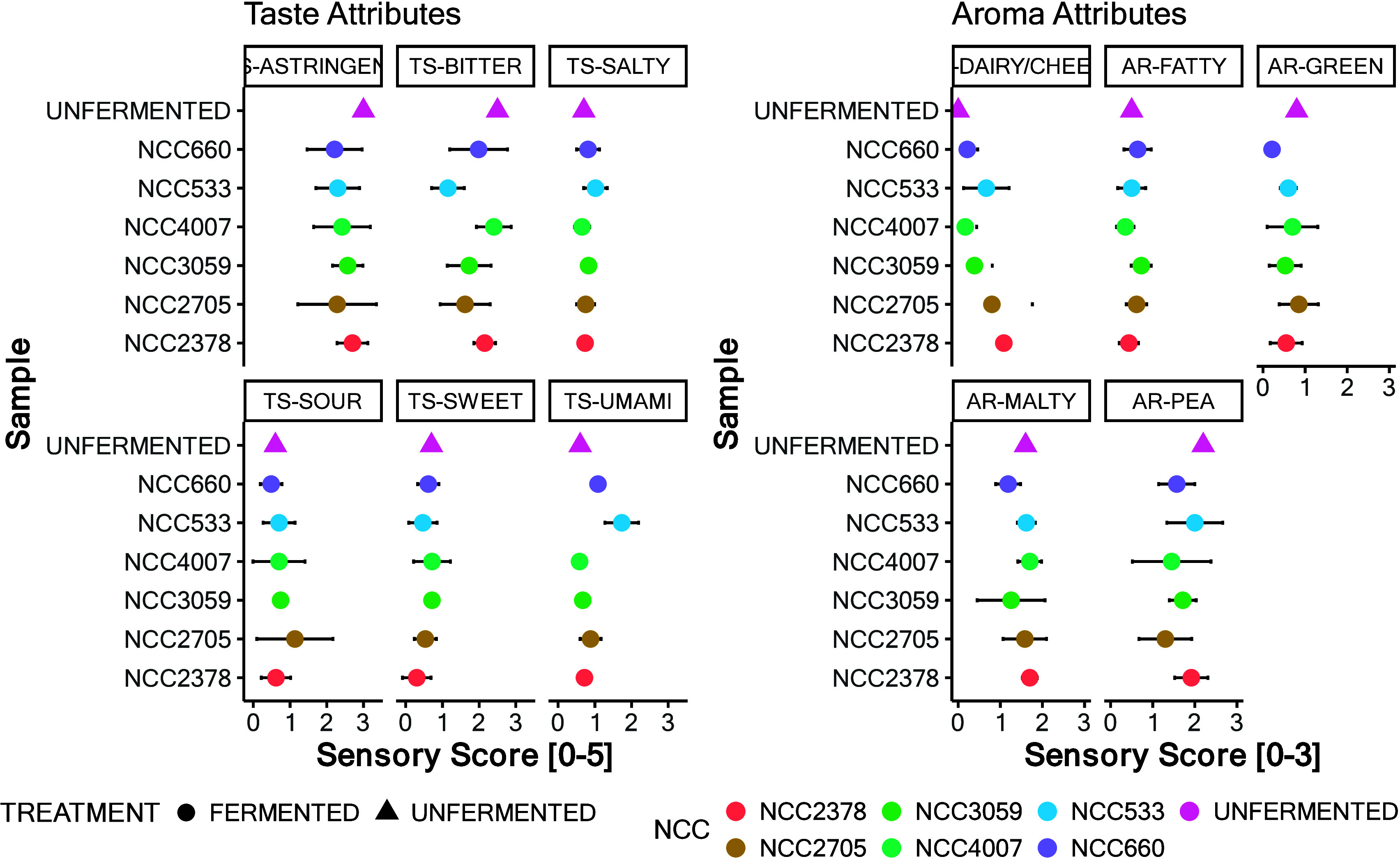
Sensory profile analysis from the third screening round. depicts the outcomes of the sensory profile analysis from the third screening round, focusing on samples subjected to a 48 h incubation period. The reference point for comparison is the unfermented product, positioned at the top of the *y*-axis. This figure illustrates the mean sensory scores of the unfermented baseline, against which the sensory attributes of the 48 h fermented samples were evaluated by panelists. Both the mean values and standard deviation for these assessments are depicted.

In the sensory evaluation of aroma and taste profiles, consistent with findings from the previous two rounds: bacterial strains such as *L. rhamnosus*, *L. lactis* subsp. *lactis*, *L. fermentum*, *L. johnsonii*, and *B. longum* were observed to reduce pea-like and green aroma attributes, as summarized in Figure 5 and detailed in SI, Table S13. In particular, *L. fermentum* NCC660 notably diminished the green attribute. Most strains, particularly *L. lactis* and *L. johnsonii*, increased the dairy/cheese aroma, whereas no significant changes were noted in the fatty and malty aromas. In addition, taste profile (Figure 5 and SI, Table S14) analysis showed that these bacteria reduced bitterness, an effect observed especially for strains such as *L. fermentum* (NCC660 and NCC3059), *L. johnsonii* NCC533, and *L. lactis* subsp. *lactis* NCC2378, and *B. longum* NCC2705. Umami and salty tastes were significantly enhanced by fermentation with *L. johnsonii* NCC533 and *L. fermentum* NCC660. These results aligned with those observed in previous rounds.

#### Untargeted and Targeted Metabolomics

**Figure 6:**
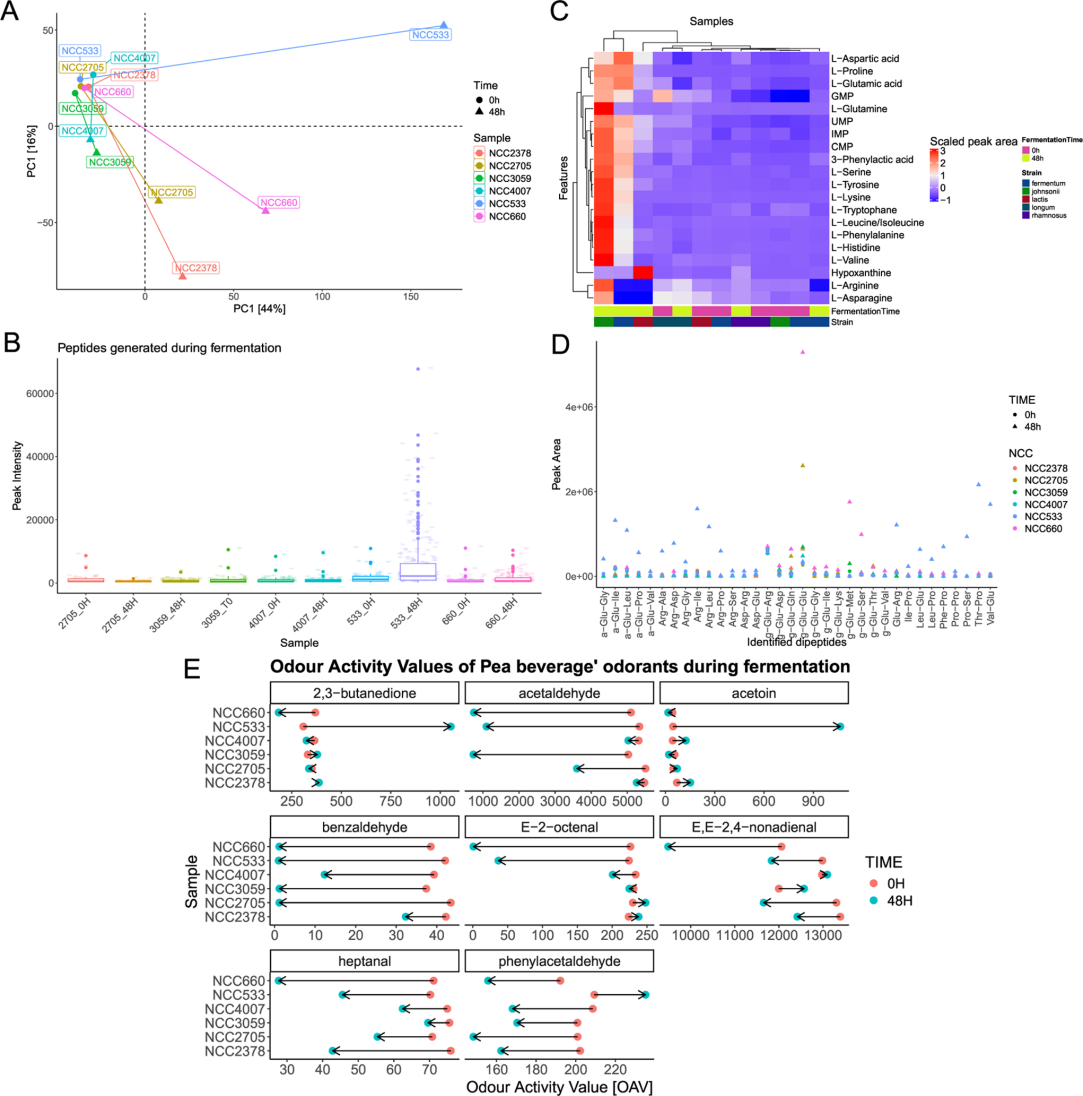
A multipanel visual summary of the analytical results from the third screening round. (A) Biplot obtained from the principal component analysis of the untargeted metabolomics data. (B) Boxplots of peptide intensities obtained from the peptidomics screening for peptides generated from *Pisum sativum* storage protein. (C) Heatmap displaying the relevant changing features and their peak areas obtained from the untargeted metabolomics alignment results of the third screening round of the fermented products. (D) The peak areas of the identified dipeptides in the fermented products. (E) The odor activity values (OAVs) of the odorants, which considerably changed during the fermentation of pea beverages with different strains in round 3. Quantified odorants face the plot. Points indicate the OAV of a particular analyte for a particular fermented product. Points are colored based on fermentation time, and arrows indicate the direction of the change after 48 h of fermentation. Further data, including the additional identified odorants, have been reported in the SI.

To get deeper inside of the chemical compounds and compound classes responsible for the perceived sensory shifts during fermentation, additional targeted and untargeted metabolomics and proteomics measurements were performed. For example, Figure 6A shows a PCA biplot of untargeted metabolomic profiles from the third round’s 48 h fermentation, revealing a time-based metabolic shift from circular to triangular shapes. *L. johnsonii*, *L. fermentum* NCC660, and *L. lactis* subsp. *lactis* NCC2378 displayed significant shifts, while *L. rhamnosus* NCC4007 and *L. fermentum* NCC3059 had minimal changes, especially in the first dimension. Analysis indicated proteolytic activity in fermentations with *L. johnsonii* NCC533 and *L. fermentum* NCC660, although it was less than that of *B. subtilis*. The peptide point plot in Figure 6B shows the highest peptide counts at 48 h in these samples, suggesting a link between chemical shifts and peptide count. Most peptides were fragments of vicilin, a key protein in *Pisum sativum*.^50^ According to the literature, proteolytic activity during the fermentation of pea protein, accompanied by decreased perceived bitterness, has previously been observed.^51^ Further details regarding the peptides produced during the fermentations of round 3 are visually depicted in SI, Figure S10.

In this study, both longer peptides (identified by MaxQuant) and shorter prolyl, glutamyl, and arginyl dipeptides (detected by targeted LC-MS/MS methods) were analyzed in fermented samples to assess their presence and intensity changes compared to unfermented samples (Figure 6D). Especially the short peptides analyzed were shown to contribute to the taste profiles of fermented foods such as parmesan cheese and fish sauce in the past.^31−33,52^ Thus, they are of significant interest in the field of plant-based alternatives, enhancing umami taste and supporting the adoption of plant-based diets.^41^

Especially, fermentation with *L. johnsonii* NCC533 and *L. fermentum* NCC660 for 48 h increased the peak area of these peptides, with *L. johnsonii* NCC533 enhancing arginyl and prolyl peptides and *L. fermentum* NCC660 increasing γ-glutamyl peptides such as γ-glutamylglycine, known for producing an umami-enhancing or kokumi taste.^52,53^

This study also detected free amino acids and nucleosides, which are known to contribute to umami and savory flavors in fermented foodstuffs (Figure 6C). *L. johnsonii* NCC533 and *L. fermentum* NCC660 significantly increased peak areas of free amino acids and nucleotides, including glutamic acids and guanosine 5′ monophosphate.

In summary, as the controlled release of amino acids, nucleosides, and peptides has been associated with increased umami perception in several fermented food items,^54,55^ these results implicate samples with proteolytic activity as a possible source of umami-tasting molecules. The debittering effect could not be explained by a reduction of bitter-tasting metabolites in pea protein; therefore, it is hypothesized that the debittering impact was associated with increased umami-tasting molecules, a phenomenon already known and described in the literature regarding other foodstuffs.^56^ Further investigations using quantitation analysis and recombination experiments (sensomics/sensoproteomics) are necessary to mechanistically explain the improvement in flavor of pea protein products by *L. johnsonii* NCC533 and *L. fermentum* NCC660 beyond a mere correlation and will be published in a separated paper.

#### Aroma Quantitation

We quantified key food odorants in pea protein isolates and dairy products to assess aroma profile changes during fermentation, detailing exact concentrations and odor activity values (OAVs) in SI, Table S15 and Figure 6E, respectively. Our analysis revealed that all tested strains significantly reduced the OAVs of key aldehydes, including hexanal (**20**, Figure 1) and benzaldehyde (**17**, Figure 1), with specific reductions in (*E*)-2-octenal (**18**, Figure 1) by *L. johnsonii* and *L. fermentum*. This led to a notable decline in green and pea-like aroma notes, aligning with sensory analysis outcomes depicted in Figures 3 and 5. Additionally, the increase in diacetyl and acetoin OAVs corresponded with heightened dairy notes in products fermented by *L. johnsonii* NCC533 and *L. lactis* NCC2378. *L. fermentum* NCC660 stood out for its remarkable efficacy in diminishing green, pea-like, and aldehyde-related aromas, including hexanal, (*E*)-2-octenal, heptanal (**16**, see Figure 1), and acetaldehyde (**23**, see Figure 1).

Reducing aldehydes during the fermentation of plant-based protein beverages and consequently removing their green and beany aroma notes is a well-known phenomenon, and these results are consistent with those previously reported in the literature regarding the fermentation of plant-based beverages with lactic acid bacteria.^17,18,21,24,49,57^ This study has thus confirmed with further evidence the suitability of lactic acid fermentation as a biotechnological methodology to improve the sensory quality of plant-based beverages.

## Conclusion

This study conducted three screening rounds to identify strains that improved the sensory attributes of pea-protein-based beverages. The findings showed significant taste and aroma enhancements after 48 h of fermentation, including reduced bitterness and richer umami and dairy-like aromas. Strains such as *L. johnsonii* NCC533 and *L. fermentum* NCC660 effectively enhanced flavor without drastic metabolomic changes, offering debittering effects, increased umami and salty tastes, and enriched amino acids and peptides. The aroma improvements involved the reduction of green-beany off-flavors and the introduction of pleasant notes, as confirmed by quantitative aroma compound analysis.

Among the tested strains, *L. fermentum* NCC3059, *L. fermentum* NCC660, *L. lactis* NCC2378, *L. johnsonii* NCC533, *L. rhamnosus* NCC4007, and *B. longum* NCC2705 stood out as effective starter cultures. The results of this study emphasize the importance of empirical screening, focusing on how cultures impact a beverage’s metabolome and sensory qualities. However, *in vivo* sensory assessments are also crucial for validation. Early rounds with *Bacillus* produced significant metabolic changes but led to excessive peptide production, negatively impacting taste. This phenomenon highlights the need for comprehensive screening that combines analytical chemistry and sensory evaluation, cautioning against relying solely on analytics or *in silico* methods. In summary, the findings of this research underscore the importance of empirical screening in selecting starter cultures for pea protein beverage fermentation, linking metabolomic changes to sensory enhancements, and supporting a multidimensional assessment approach to developing more appealing products. In conclusion, this study helped to identify key starter cultures from a large starter collection that can enhance the flavor of pea protein food products. Choosing the right culture is the first and most crucial step. The next phase of research will focus on understanding the mechanisms by which these cultures improve flavor, specifically examining the key flavor actives and the metabolic processes involved as published in our companion paper.^58^

## Supporting Information Available

Flow cytometry data; untargeted metabolomics and peptidomics additional graphs; visual representation of the aroma profile descriptive analysis; details of the starter cultures from round 1 and taxonomy; microbial growth conditions employed in the first round; SWATH-MS parameters; LC-MS details of the pure standards used for identification in HILIC-mode separation; aroma compounds used for attribute training; flow cytometry data; UHPLC-TOF-MS data of features selected during the first and second round; sensory data from first and second round; aroma quantitation data (PDF)
