# Supplementary material for: Screening of a Microbial Culture Collection: Empowering Selection of Starters for Enhanced Sensory Attributes of Pea-Protein-Based Beverages
Source: J Agric Food Chem. 2024 Jul 2;72(28):15890–905. doi: 10.1021/acs.jafc.4c02316 (PMC11261627; doi:10.1021/acs.jafc.4c02316)
Supplement: Supplementary file 1 — jf4c02316_si_001.pdf [file jf4c02316_si_001.pdf]

Supplementary Information

Screening of a microbial culture collection: Empowering selection of starters for enhanced sensory attributes of pea-protein-based beverages

Andrea Spaccasassi [†*], Florian Utz [†], Andreas Dunkel[''], Rosa Aragao Börner [#], Lijuan Ye [#], Filippo De Franceschi [#], Biljana Bogicevic [#], Arne Glabasnia[#], Thomas Hofmann [†], and Corinna Dawid [^†*1]

[†] Chair of Food Chemistry and Molecular and Sensory Science, TUM School of Life Sciences, Technical University of Munich, Lise-Meitner-Str. 34, 85354 Freising, Germany

* TUM CREATE, 1 CREATE Way, #10-02 CREATE Tower, Singapore, 138602 Singapore

[''] Leibniz-Institute for Food Systems Biology at the Technical University of Munich, 85354 Freising, Germany

Société des Produits Nestlé S.A., Nestlé Research, Route du Jorat 57, CH 1000 Lausanne 26, Switzerland

^ Professorship for Functional Phytometabolomics, TUM School of Life Sciences, Technical University of Munich, Lise-Meitner-Str. 34, 85354 Freising, Germany

Running Title: Microbes that improve pea-protein beverage flavor.

---

[1] **To whom correspondence should be addressed:**

PHONE +49-8161-712902

FAX +49-8161-712949

E-MAIL corinna.dawid@tum.de

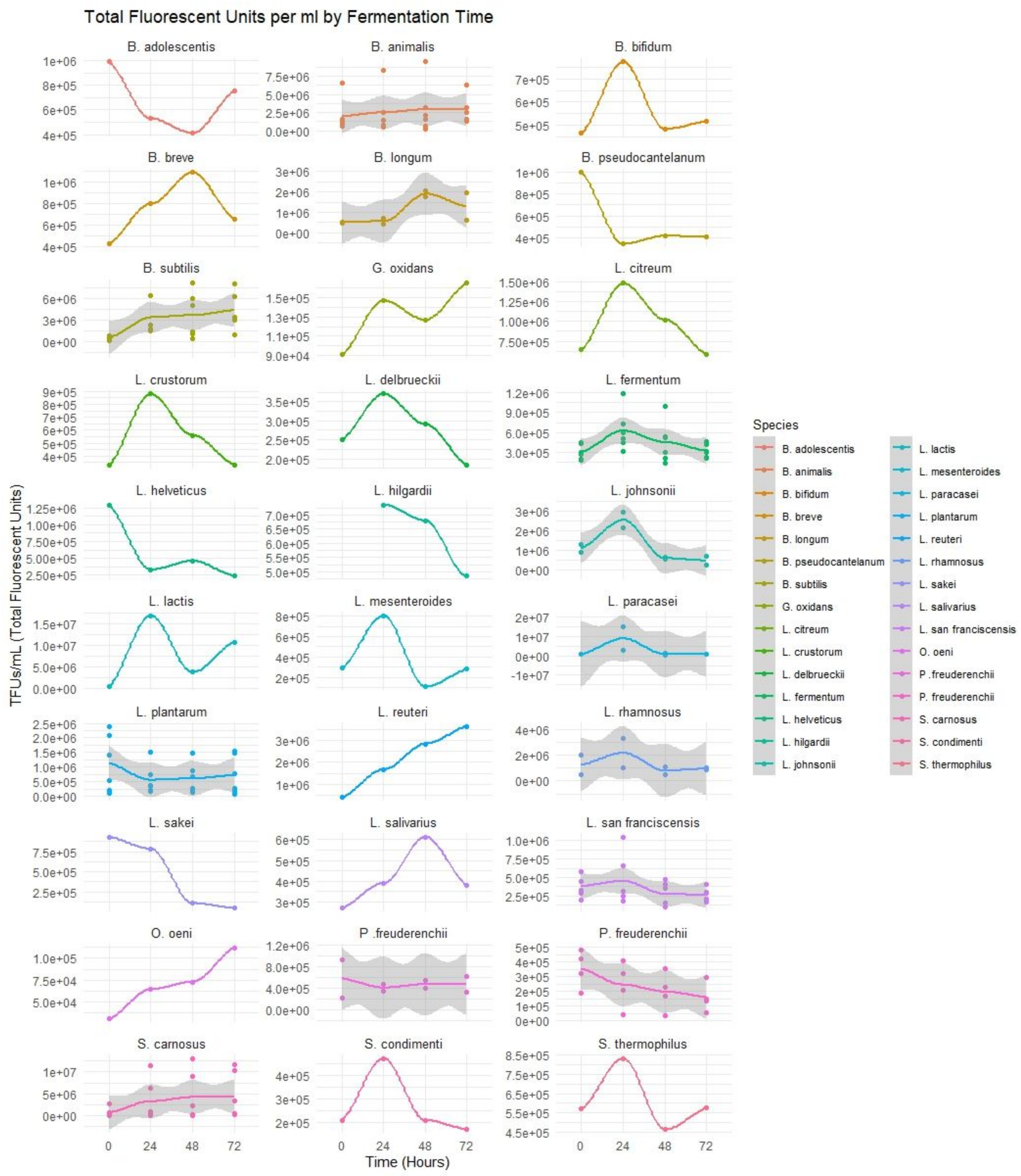

Figure S1

Quantitative assessment of total bacterial concentration in TFUs/ml (Total Fluorescent Units) for diverse bacterial strains tested during the first screening round over a period of 72 hours of fermentation, illustrating the growth dynamics and population changes of each strain over time. rior to visualization, the dataset underwent a filtering process to ensure the integrity and completeness of the data analyzed. Specifically, two bacterial species, *Pediococcus parvulus* and *Enterococcus faecium* (E. faecium), were identified as having incomplete data points across the measured time intervals.

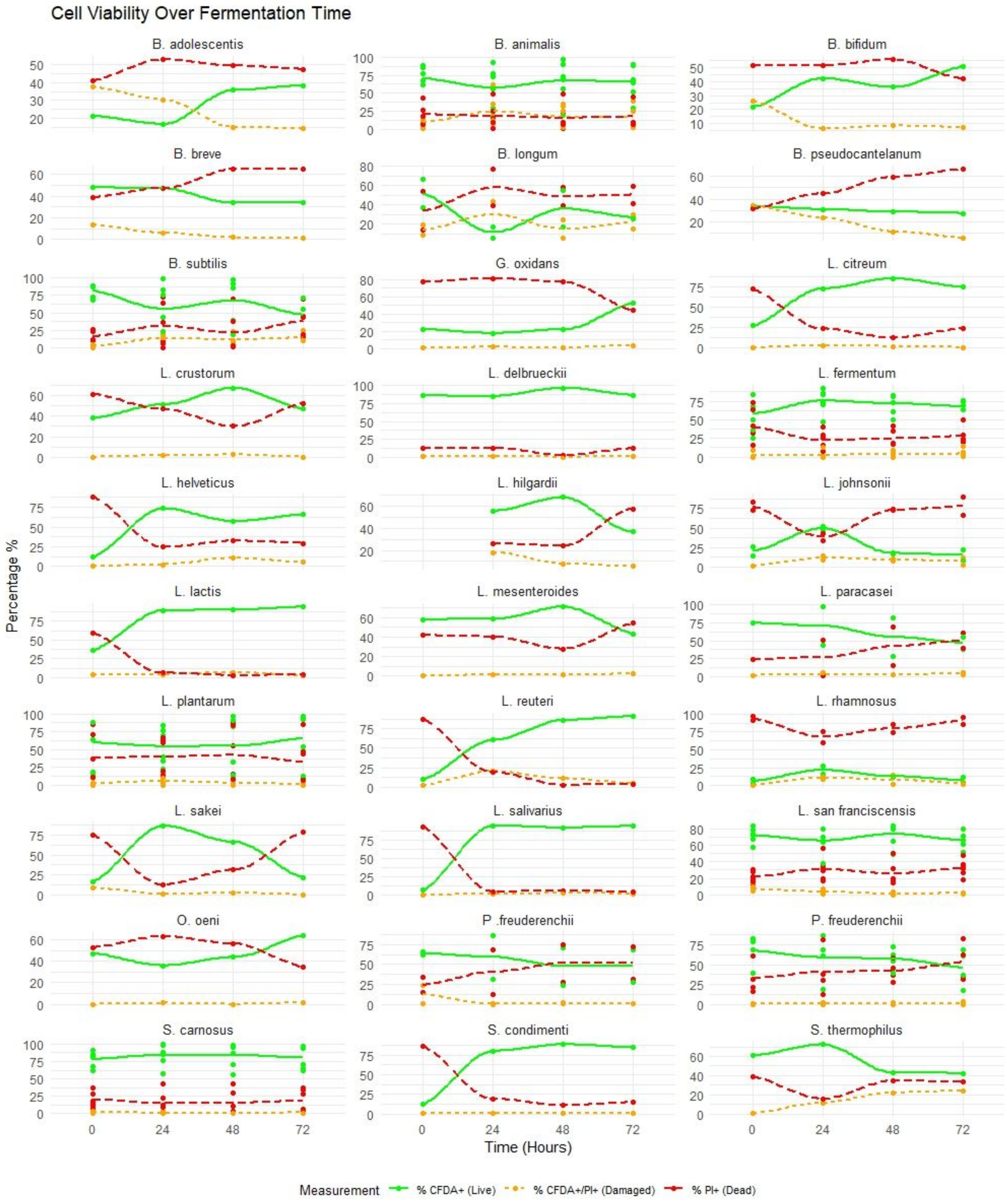

Figure S2

Graphical representation of cell viability in various bacterial strains during fermentation of pea beverage of round 1 over a 72-hour period, measured by percentage of live cells (CFDA+), damaged cells (CFDA+/PI+), and dead cells (PI+).

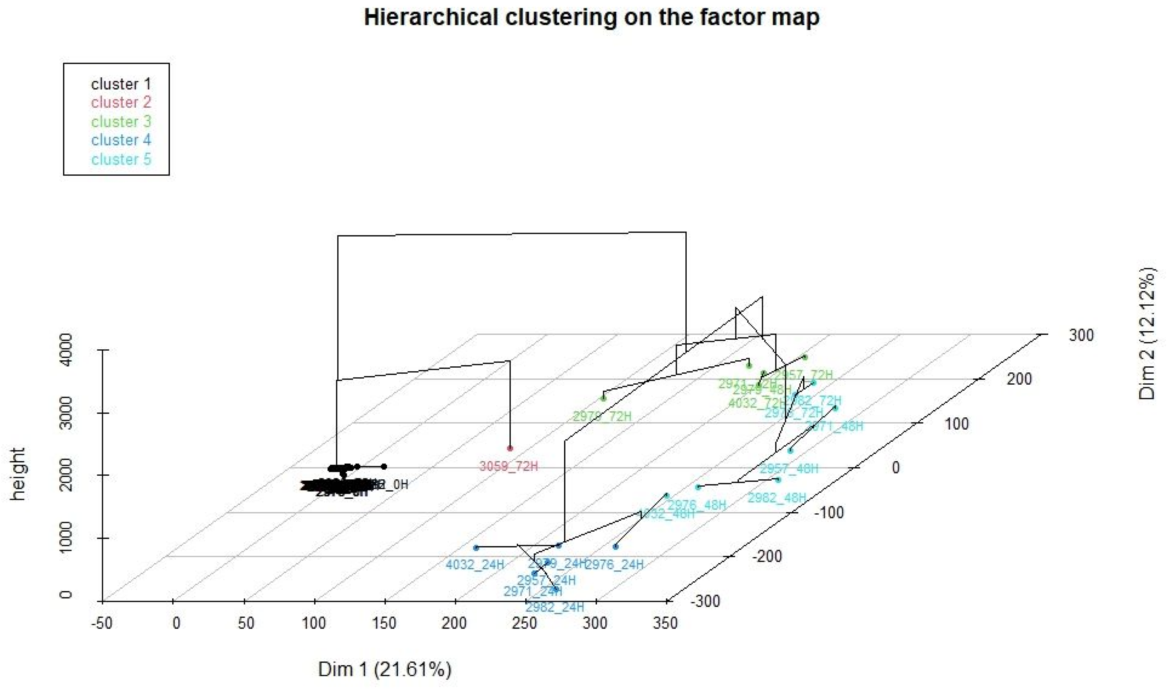

Figure S3

Hierarchical clustering on the factorial map. Figure is a three-dimensional plot combining the hierarchical clustering and the factorial map obtained from HCPC() function in FactoMineR package, which is used to compute hierarchical clustering on principal components. The analysis was performed on the alignment file obtained from the study of the untargeted data obtained in Round 1. Negative and positive polarities were combined in a single matrix.

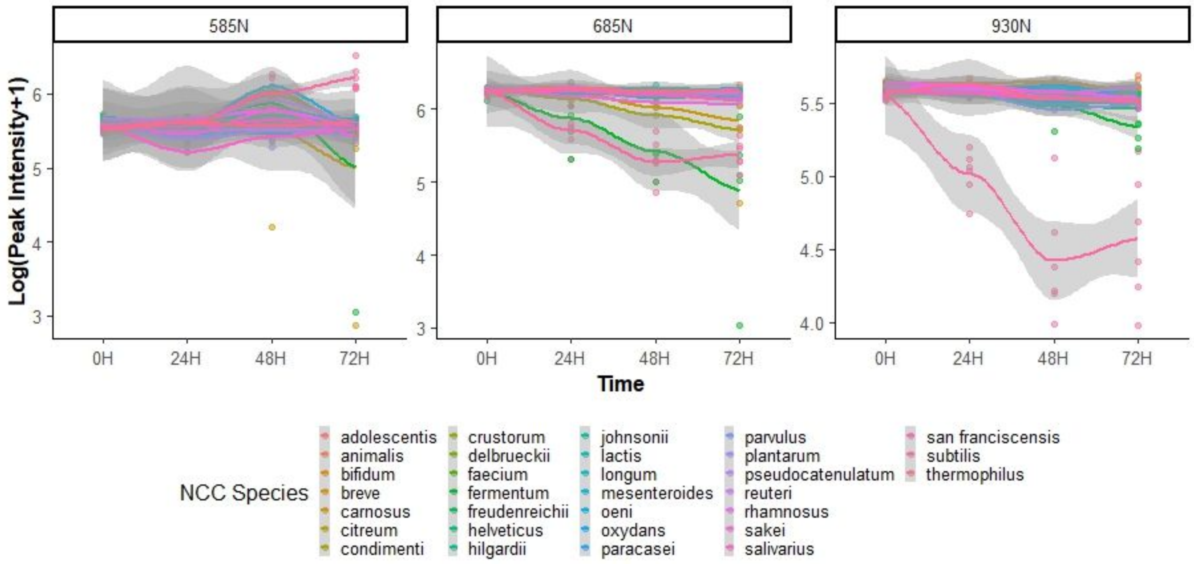

Figure S4

Line plot indicating the longitudinal change in the logarithmically transformed peak area of features belonging to the class of C18 fatty acids and their oxidation products extracted from the alignment file obtained from the untargeted metabolomics data from round 1. Feature 585N corresponds to Linoleic acid, Feature 685N to 13-Hydroxyoctadeca-9,11-dienoic acid (13-HODE), and feature 930N to 9,10,13-Trihydroxy-11-octadecenoic acid (9,10,11-TriHOME). Details of on the mass spectra, retention time and identification are detailed in Table S5.

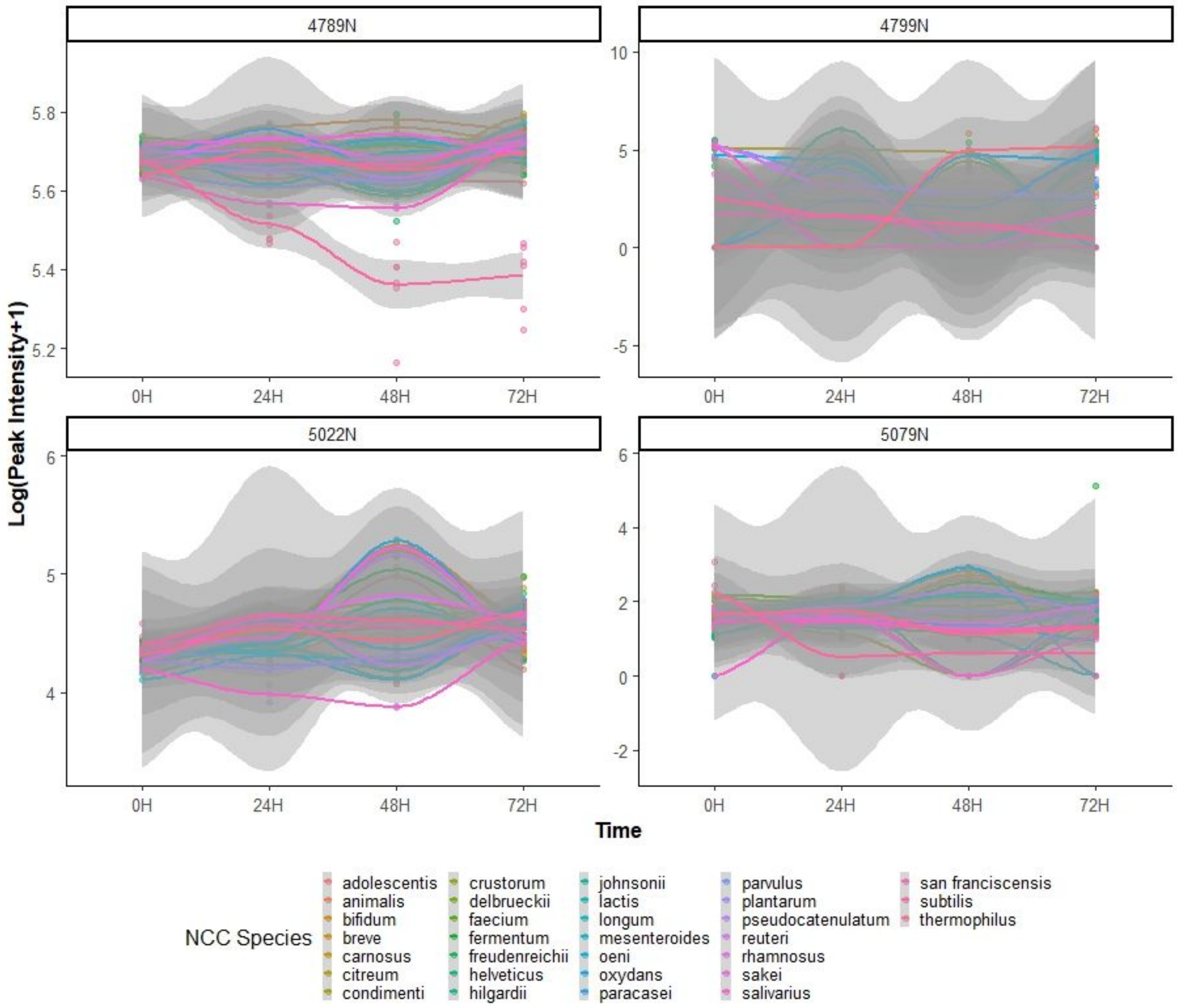

Figure S5

Line plot indicating the longitudinal change in the logarithmically transformed peak area of features belonging to the class of saponins extracted from the alignment file obtained from the untargeted metabolomics data from round 1. Details of on the mass spectra, retention time and identification are detailed in Table S5. Following the feature numbers and their tentative identification annotated with MSFINDER software: 4789N Dehydrosoyasaponin I, 4799N Soyasaponin I, 5022N Pisumsaponin I, 4758N Soyasaponin Bg.

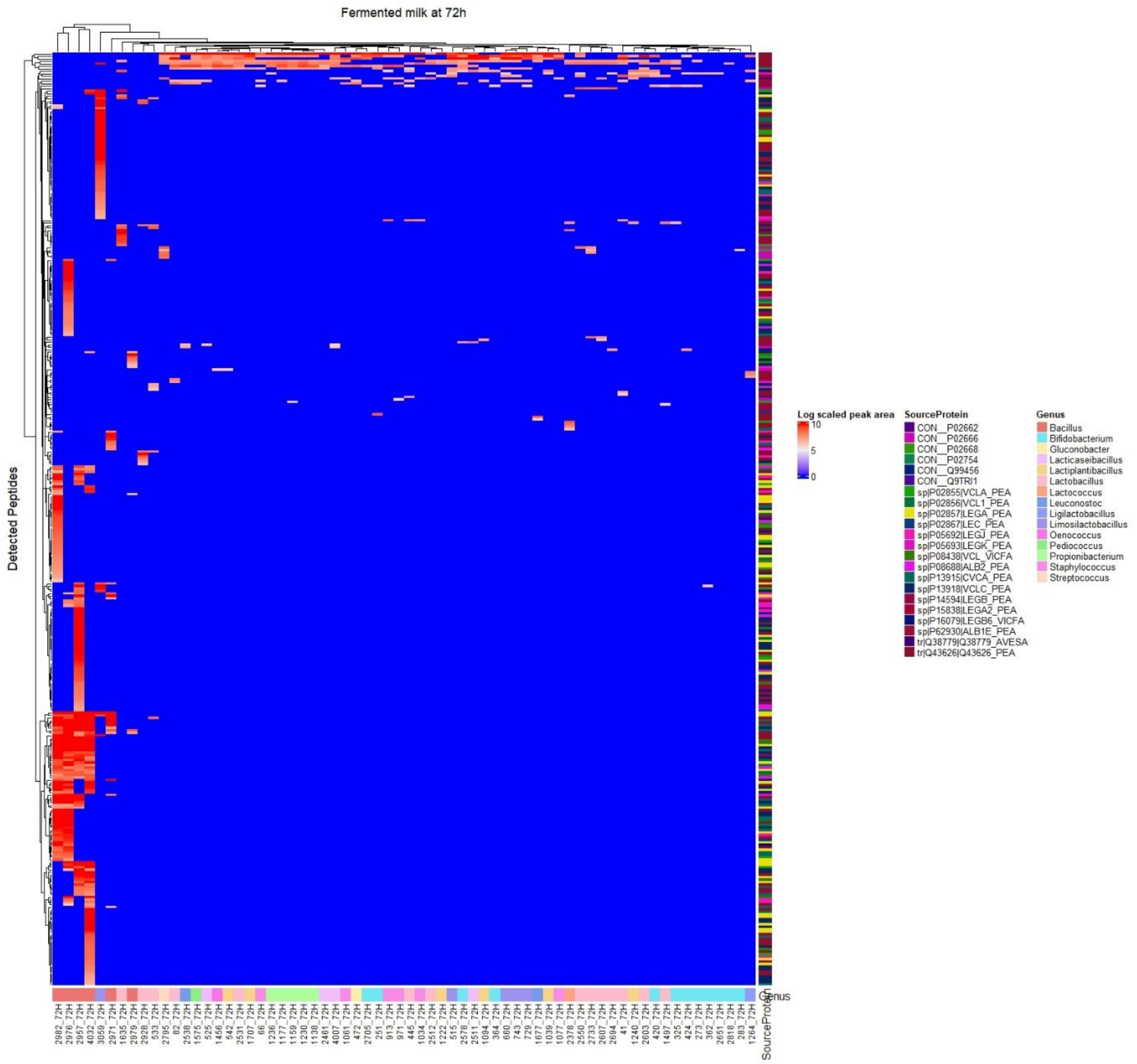

Figure S6

This figure displays a peptidomics heatmap that visualizes the peptides detected in each 72h fermented sample analyzed in Round 1, along with their scaled and centered peak areas, as derived from MaxQuant peptidomic deconvolution, as detailed in the methods section. Each sample is labeled according to the strain used as the inoculum, identified by its NCC number and its fermentation time (72H for 72 hours fermented). For instance, a pea beverage fermented with L. *johnsonii* NCC533 for 72 hours is denoted as 533_72H.

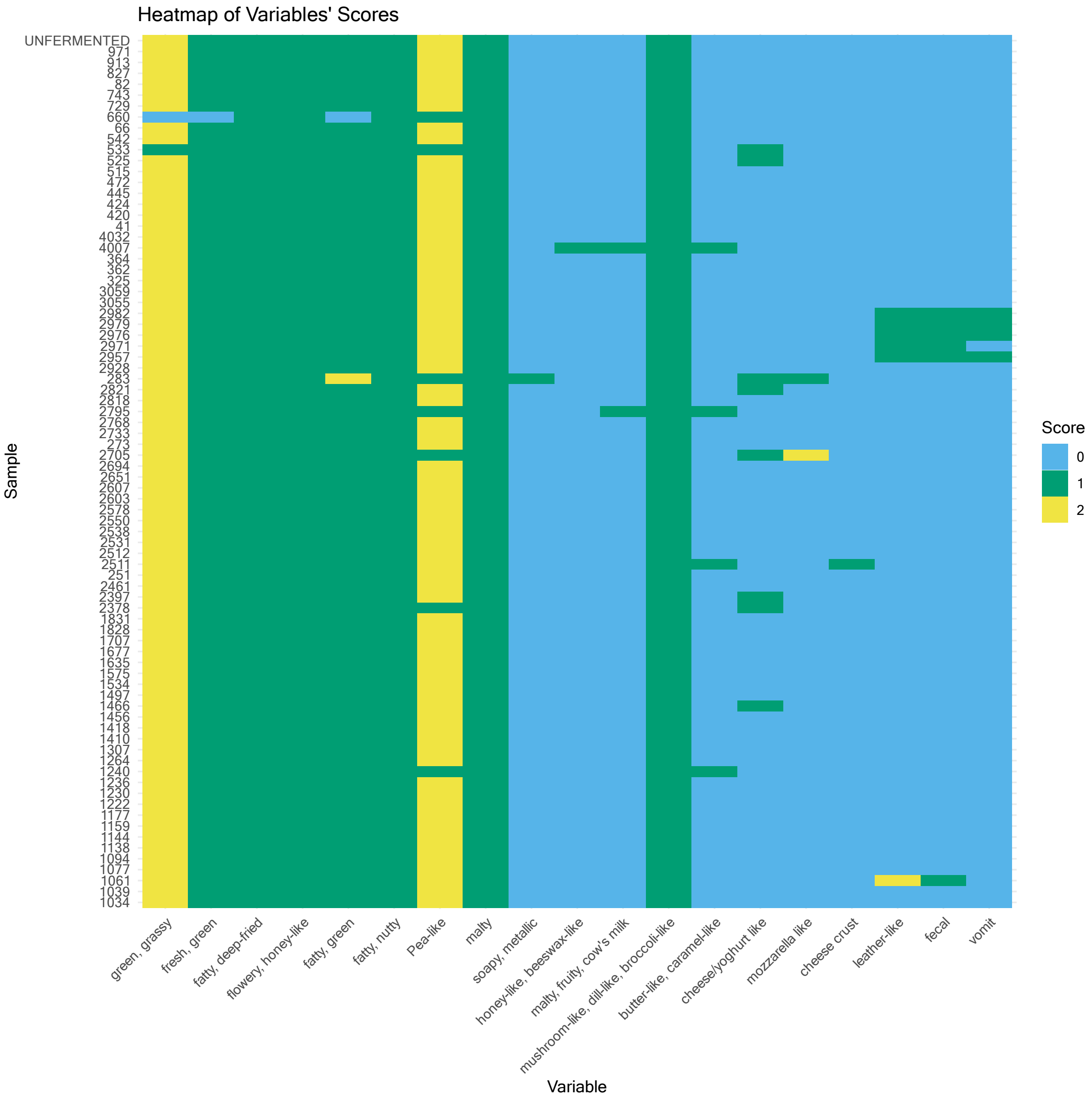

Figure S7: Heatmap of Aroma Profile Scores by Sample. This heatmap visualizes the sensory profile scores ranging from 0 to 4 for various samples across multiple variables, including descriptors. When an attribute was perceived by the panelist this was rated as 1 (Barely recognizable), 2 (Slight), 3 (Moderate), 4 (Strong). When an attribute was not identified this was filled with 0 (Not present). Each tile represents the score of a particular sensory attribute for a given sample, with the sample names listed on the y-axis and sensory descriptors on the x-axis. The color scalecorresponds to scores from 0 to , with 3 and 4 not diplayed due to the lack of score in that region, indicating the intensity of each sensory attribute.

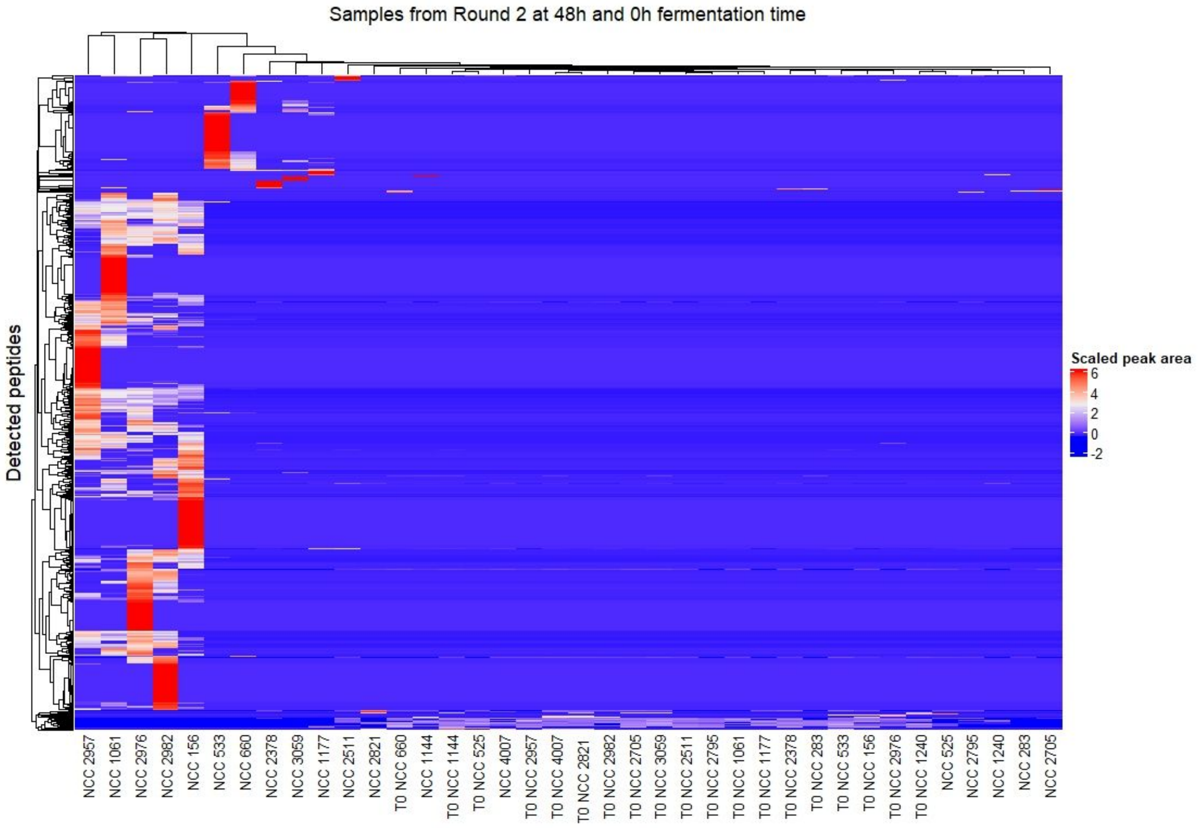

Figure S8

This figure displays a peptidomics heatmap that visualizes the peptides detected in each sample analyzed in Round 2, along with their scaled and centered peak areas, as derived from MaxQuant peptidomic deconvolution, as detailed in the methods section. Each sample is labeled according to its fermentation time (T0 for unfermented or T48 for 48 hours fermented) and the strain used as the inoculum, identified by its NCC number. For instance, a pea beverage fermented with L. *johnsonii* NCC533 for 48 hours is denoted as 'T48_NCC533'.

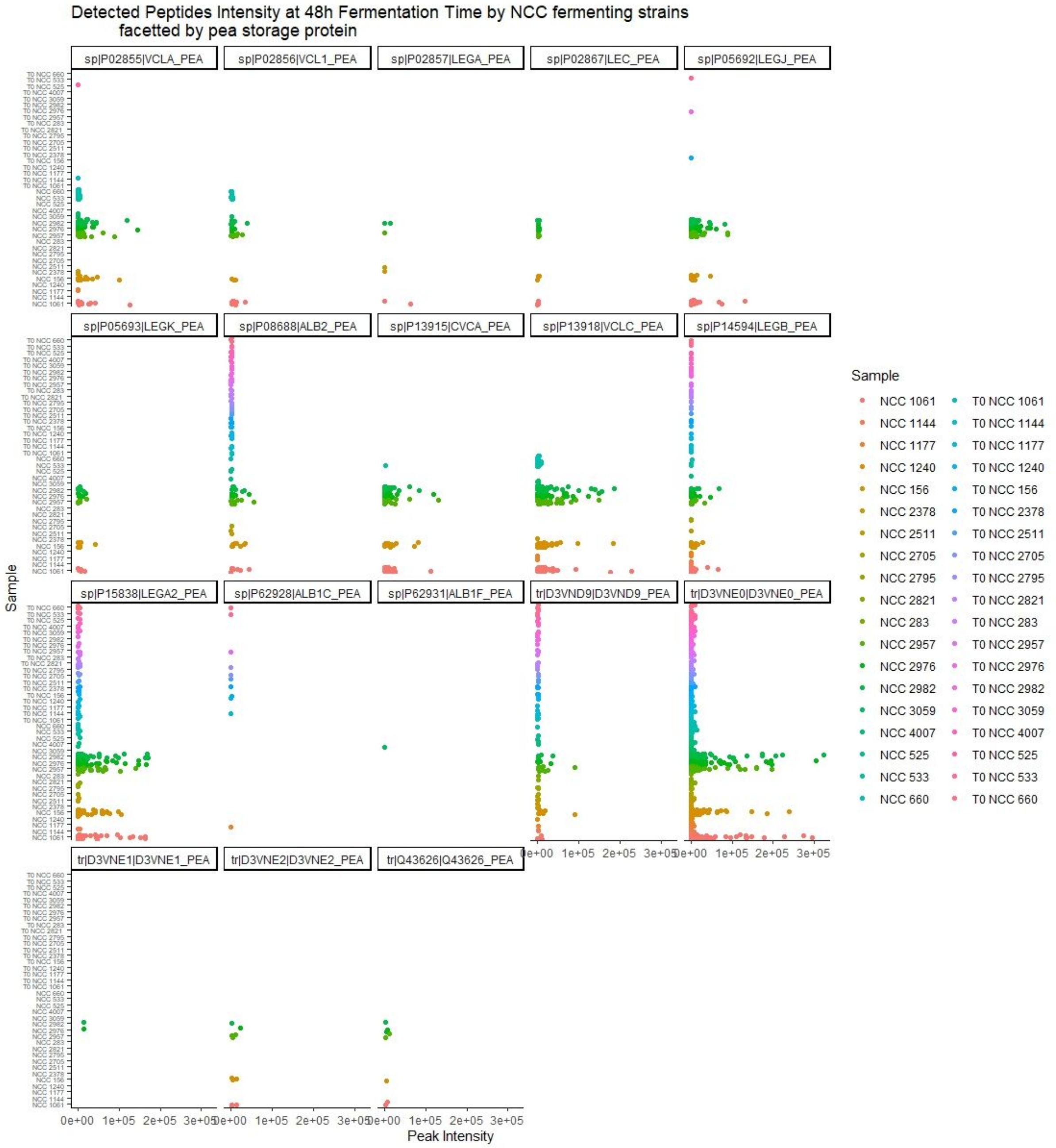

Figure S9

This figure presents multipanel point plots showcasing the peptides generated from each fermented sample in Round 3, with the plots organized by Pisum sativum storage protein. The y-axis labels the samples used during Round 2, identified by their NCC number and fermentation time (formatted as NCCnumber_fermentationtime), while the x-axis represents the peak intensity of the deconvoluted peptides as determined by the MaxQuant analysis, as detailed in the corresponding methods section. Each panel is dedicated to a different *Pisum sativum* storage protein, facilitating a comparative analysis of peptide profiles across the various samples.

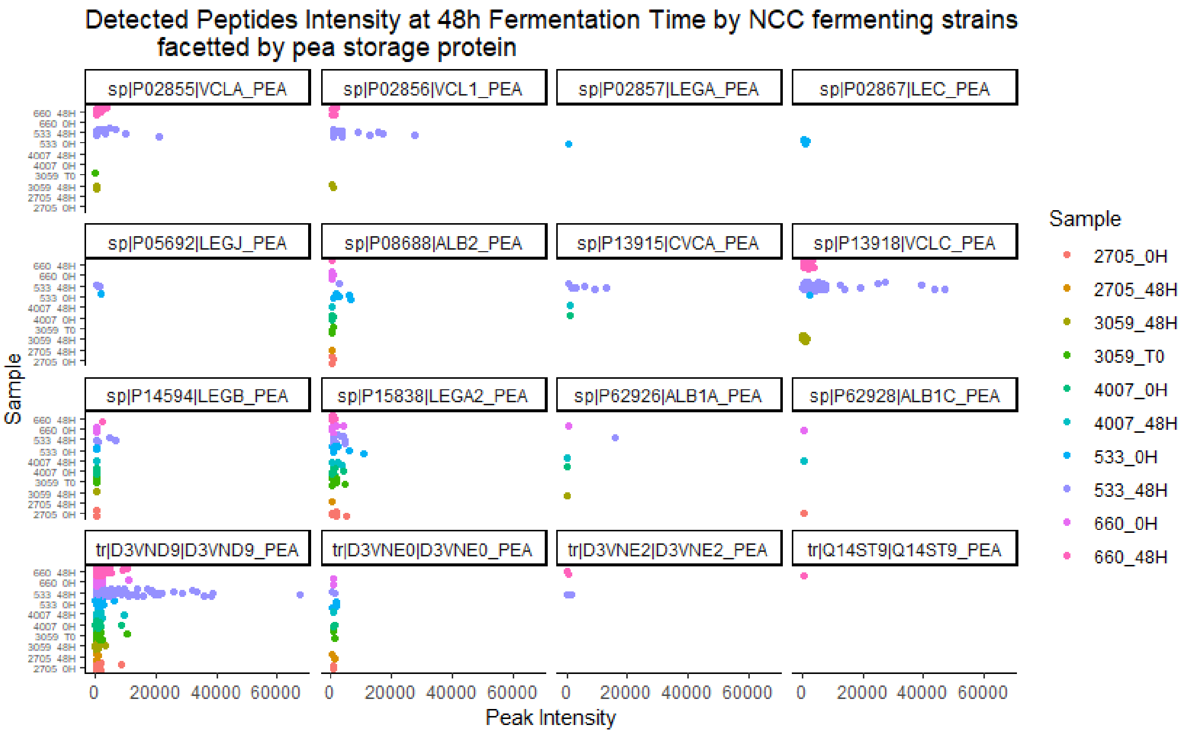

Figure S10

This figure presents multipanel point plots showcasing the peptides generated from each fermented sample in Round 3, with the plots organized by Pisum sativum storage protein. The y-axis labels the samples used during Round 3, identified by their NCC number and fermentation time (formatted as NCCnumber_fermentationtime), while the x-axis represents the peak intensity of the deconvoluted peptides as determined by the MaxQuant analysis, as detailed in the corresponding methods section. Each panel is dedicated to a different Pisum sativum storage protein, facilitating a comparative analysis of peptide profiles across the various samples.

Table S1

This table outlines the bacteria strains from the Nestlé Culture Collection (NCC) utilized in the study, along with their detailed taxonomic classification. It specifies the NCC strain numbers, uniquely identifying each culture, and classifies each strain by phylum, class, order, family, genus, and species.

NCC strain	Species	Phylum	Class	Order	Family	Genus
41	*Lactobacillus sakei*	*Firmicutes*	*Bacilli*	*Lactobacillales*	*Lactobacillaceae*	*Lactobacillus*
66	*Staphylococcus condimenti*	*Firmicutes*	*Bacilli*	*Bacillales*	*Staphylococcaceae*	*Staphylococcus*
82	*Lactobacillus delbrueckii*	*Firmicutes*	*Bacilli*	*Lactobacillales*	*Lactobacillaceae*	*Lactobacillus*
251	*Bifidobacterium adolescentis*	*Actinobacteria*	*Actinobacteria*	*Bifidobacteriales*	*Bifidobacteriaceae*	*Bifidobacterium*
273	*Bifidobacterium animalis*	*Actinobacteria*	*Actinobacteria*	*Bifidobacteriales*	*Bifidobacteriaceae*	*Bifidobacterium*
283	*Bifidobacterium longum*	*Actinobacteria*	*Actinobacteria*	*Bifidobacteriales*	*Bifidobacteriaceae*	*Bifidobacterium*
325	*Bifidobacterium pseudocatenulatum*	*Actinobacteria*	*Actinobacteria*	*Bifidobacteriales*	*Bifidobacteriaceae*	*Bifidobacterium*
362	*Bifidobacterium animalis*	*Actinobacteria*	*Actinobacteria*	*Bifidobacteriales*	*Bifidobacteriaceae*	*Bifidobacterium*
364	*Bifidobacterium breve*	*Actinobacteria*	*Actinobacteria*	*Bifidobacteriales*	*Bifidobacteriaceae*	*Bifidobacterium*
420	*Bifidobacterium bifidum*	*Actinobacteria*	*Actinobacteria*	*Bifidobacteriales*	*Bifidobacteriaceae*	*Bifidobacterium*
424	*Bifidobacterium animalis*	*Actinobacteria*	*Actinobacteria*	*Bifidobacteriales*	*Bifidobacteriaceae*	*Bifidobacterium*
445	*Lactobacillus san franciscensis*	*Firmicutes*	*Bacilli*	*Lactobacillales*	*Lactobacillaceae*	*Lactobacillus*
472	*Gluconobacter oxydans*	*Proteobacteria*	*Alphaproteobacteria*	*Rhodospirillales*	*Acetobacteraceae*	*Gluconobacter*
515	*Limosilactobacillus fermentum*	*Firmicutes*	*Bacilli*	*Lactobacillales*	*Lactobacillaceae*	*Limosilactobacillus*
525	*Lacticaseibacillus rhamnosus*	*Firmicutes*	*Bacilli*	*Lactobacillales*	*Lactobacillaceae*	*Lacticaseibacillus*
533	*Lactobacillus johnsonii*	*Firmicutes*	*Bacilli*	*Lactobacillales*	*Lactobacillaceae*	*Lactobacillus*
542	*Lactiplantibacillus plantarum*	*Firmicutes*	*Bacilli*	*Lactobacillales*	*Lactobacillaceae*	*Lactiplantibacillus*
660	*Limosilactobacillus fermentum*	*Firmicutes*	*Bacilli*	*Lactobacillales*	*Lactobacillaceae*	*Limosilactobacillus*
692	*Limosilactobacillus fermentum*	*Firmicutes*	*Bacilli*	*Lactobacillales*	*Lactobacillaceae*	*Limosilactobacillus*
729	*Limosilactobacillus fermentum*	*Firmicutes*	*Bacilli*	*Lactobacillales*	*Lactobacillaceae*	*Limosilactobacillus*

743	*Limosilactobacillus fermentum*	*Firmicutes*	*Bacilli*	*Lactobacillales*	*Lactobacillaceae*	*Limosilactobacillus*
827	*Staphylococcus carnosus*	*Firmicutes*	*Bacilli*	*Bacillales*	*Staphylococcaceae*	*Staphylococcus*
913	*Staphylococcus carnosus*	*Firmicutes*	*Bacilli*	*Bacillales*	*Staphylococcaceae*	*Staphylococcus*
971	*Staphylococcus carnosus*	*Firmicutes*	*Bacilli*	*Bacillales*	*Staphylococcaceae*	*Staphylococcus*
1034	*Staphylococcus carnosus*	*Firmicutes*	*Bacilli*	*Bacillales*	*Staphylococcaceae*	*Staphylococcus*
1039	*Lactiplantibacillus plantarum*	*Firmicutes*	*Bacilli*	*Lactobacillales*	*Lactobacillaceae*	*Lactiplantibacillus*
1061	*Staphylococcus carnosus*	*Firmicutes*	*Bacilli*	*Bacillales*	*Staphylococcaceae*	*Staphylococcus*
1077	*Staphylococcus carnosus*	*Firmicutes*	*Bacilli*	*Bacillales*	*Staphylococcaceae*	*Staphylococcus*
1094	*Lactiplantibacillus plantarum*	*Firmicutes*	*Bacilli*	*Lactobacillales*	*Lactobacillaceae*	*Lactiplantibacillus*
1138	*Propionibacterium freudenreichii*	*Actinobacteria*	*Actinobacteria*	*Actinomycetales*	*Propionibacteriaceae*	*Propionibacterium*
1159	*Propionibacterium freudenreichii*	*Actinobacteria*	*Actinobacteria*	*Actinomycetales*	*Propionibacteriaceae*	*Propionibacterium*
1177	*Propionibacterium freudenreichii*	*Actinobacteria*	*Actinobacteria*	*Actinomycetales*	*Propionibacteriaceae*	*Propionibacterium*
1222	*Lactiplantibacillus plantarum*	*Firmicutes*	*Bacilli*	*Lactobacillales*	*Lactobacillaceae*	*Lactiplantibacillus*
1230	*Propionibacterium freudenreichii*	*Actinobacteria*	*Actinobacteria*	*Actinomycetales*	*Propionibacteriaceae*	*Propionibacterium*
1236	*Propionibacterium freudenreichii*	*Actinobacteria*	*Actinobacteria*	*Actinomycetales*	*Propionibacteriaceae*	*Propionibacterium*
1240	*Lactiplantibacillus plantarum*	*Firmicutes*	*Bacilli*	*Lactobacillales*	*Lactobacillaceae*	*Lactiplantibacillus*
1264	*Ligilactobacillus salivarius*	*Firmicutes*	*Bacilli*	*Lactobacillales*	*Streptococcaceae*	*Ligilactobacillus*
1456	*Oenococcus oeni*	*Firmicutes*	*Bacilli*	*Lactobacillales*	*Leuconostocaceae*	*Oenococcus*
1497	*Lactobacillus hilgardii*	*Firmicutes*	*Bacilli*	*Lactobacillales*	*Lactobacillaceae*	*Lactobacillus*
1575	*Pediococcus parvulus*	*Firmicutes*	*Bacilli*	*Lactobacillales*	*Lactobacillaceae*	*Pediococcus*
1635	*Lactobacillus helveticus*	*Firmicutes*	*Bacilli*	*Lactobacillales*	*Lactobacillaceae*	*Lactobacillus*
1677	*Leuconostoc mesenteroides*	*Firmicutes*	*Bacilli*	*Lactobacillales*	*Leuconostocaceae*	*Leuconostoc*
1707	*Lactiplantibacillus plantarum*	*Firmicutes*	*Bacilli*	*Lactobacillales*	*Lactobacillaceae*	*Lactiplantibacillus*
2378	*Lactococcus lactis*	*Firmicutes*	*Bacilli*	*Lactobacillales*	*Streptococcaceae*	*Lactococcus*
2461	*Lacticaseibacillus paracasei*	*Firmicutes*	*Bacilli*	*Lactobacillales*	*Lactobacillaceae*	*Lacticaseibacillus*
2511	*Lacticaseibacillus paracasei*	*Firmicutes*	*Bacilli*	*Lactobacillales*	*Lactobacillaceae*	*Lacticaseibacillus*

2512	*Lactobacillus san franciscensis*	*Firmicutes*	*Bacilli*	*Lactobacillales*	*Lactobacillaceae*	*Lactobacillus*
2531	*Lactobacillus crustorum*	*Firmicutes*	*Bacilli*	*Lactobacillales*	*Lactobacillaceae*	*Lactobacillus*
2538	*Leuconostoc citreum*	*Firmicutes*	*Bacilli*	*Lactobacillales*	*Leuconostocaceae*	*Leuconostoc*
2550	*Lactobacillus san franciscensis*	*Firmicutes*	*Bacilli*	*Lactobacillales*	*Lactobacillaceae*	*Lactobacillus*
2578	*Bifidobacterium animalis*	*Actinobacteria*	*Actinobacteria*	*Bifidobacteriales*	*Bifidobacteriaceae*	*Bifidobacterium*
2603	*Lactobacillus reuteri*	*Firmicutes*	*Bacilli*	*Lactobacillales*	*Lactobacillaceae*	*Lactobacillus*
2607	*Lactobacillus san franciscensis*	*Firmicutes*	*Bacilli*	*Lactobacillales*	*Lactobacillaceae*	*Lactobacillus*
2651	*Bifidobacterium animalis*	*Actinobacteria*	*Actinobacteria*	*Bifidobacteriales*	*Bifidobacteriaceae*	*Bifidobacterium*
2694	*Lactobacillus san franciscensis*	*Firmicutes*	*Bacilli*	*Lactobacillales*	*Lactobacillaceae*	*Lactobacillus*
2705	*Bifidobacterium longum*	*Actinobacteria*	*Actinobacteria*	*Bifidobacteriales*	*Bifidobacteriaceae*	*Bifidobacterium*
2733	*Lactobacillus san franciscensis*	*Firmicutes*	*Bacilli*	*Lactobacillales*	*Lactobacillaceae*	*Lactobacillus*
2768	*Enterococcus faecium*	*Firmicutes*	*Bacilli*	*Lactobacillales*	*Enterococcaceae*	*Enterococcus*
2795	*Streptococcus thermophilus*	*Firmicutes*	*Bacilli*	*Lactobacillales*	*Streptococcaceae*	*Streptococcus*
2818	*Bifidobacterium animalis*	*Actinobacteria*	*Actinobacteria*	*Bifidobacteriales*	*Bifidobacteriaceae*	*Bifidobacterium*
2928	*Lactobacillus johnsonii*	*Firmicutes*	*Bacilli*	*Lactobacillales*	*Lactobacillaceae*	*Lactobacillus*
2957	*Bacillus subtilis*	*Firmicutes*	*Bacilli*	*Bacillales*	*Bacillaceae*	*Bacillus*
2971	*Bacillus subtilis*	*Firmicutes*	*Bacilli*	*Bacillales*	*Bacillaceae*	*Bacillus*
2976	*Bacillus subtilis*	*Firmicutes*	*Bacilli*	*Bacillales*	*Bacillaceae*	*Bacillus*
2979	*Bacillus subtilis*	*Firmicutes*	*Bacilli*	*Bacillales*	*Bacillaceae*	*Bacillus*
2982	*Bacillus subtilis*	*Firmicutes*	*Bacilli*	*Bacillales*	*Bacillaceae*	*Bacillus*
3059	*Limosilactobacillus fermentum*	*Firmicutes*	*Bacilli*	*Lactobacillales*	*Lactobacillaceae*	*Limosilactobacillus*
4007	*Lacticaseibacillus rhamnosus*	*Firmicutes*	*Bacilli*	*Lactobacillales*	*Lactobacillaceae*	*Lacticaseibacillus*
4032	*Bacillus subtilis*	*Firmicutes*	*Bacilli*	*Bacillales*	*Bacillaceae*	*Bacillus*

Table S2

This table presents the starter cultures selected from the Nestlé Culture Collection (NCC Strain) used in this study, alongside their specific incubation conditions. It details whether oxygen was present (aerobic) or absent (anaerobic) during incubation, the temperatures set for activation and incubation phases, and the cultivation media utilized. The media include MRS, MRS+Cys (MRS with cysteine), MHH, PSM-AAB, BHI, HJL, MRS (adjusted to pH 4.5), and BST. These incubation conditions were applied during the P1 and P2 activation steps to re-activate the lyophilized cultures and throughout the incubation of the inoculated pea beverage, ensuring optimal growth and activity of the starter cultures.

NCC Strains	incubation temperature (degree Celcius)	Cultivation media (activation step)	Incubation condition
41	30	MRS	aerobiosis
66	30	MRS	aerobiosis
82	40	MRS	aerobiosis
251	37	MRS CYS	anaeorobiosis
273	37	MRS CYS	anaeorobiosis
283	37	MRS CYS	anaeorobiosis
325	37	MRS CYS	anaeorobiosis
362	37	MRS CYS	anaeorobiosis
364	37	MRS CYS	anaeorobiosis
420	37	MRS CYS	anaeorobiosis
424	37	MRS CYS	anaeorobiosis
445	30	MHH	anaeorobiosis
472	30	PSM-AAB	aerobiosis
515	40	MRS	aerobiosis
525	37	MRS	aerobiosis
533	37	MRS	aerobiosis
542	30	MRS	aerobiosis
660	40	MRS	aerobiosis
692	40	MRS	aerobiosis
729	40	MRS	aerobiosis
743	40	MRS	aerobiosis
827	30	BHI	aerobiosis
913	30	BHI	aerobiosis
971	30	BHI	aerobiosis
1034	30	BHI	aerobiosis
1039	30	MRS	aerobiosis
1061	30	BHI	aerobiosis
1077	30	BHI	aerobiosis
1094	30	MRS	aerobiosis
1138	30	MRS	anaeorobiosis
1144	30	HJL	aerobiosis
1159	30	MRS	anaeorobiosis
1177	30	MRS	anaeorobiosis
1222	30	MRS	aerobiosis

1230	30	MRS	anaeorobiosis
1236	30	MRS	anaeorobiosis
1240	30	MRS	aerobiosis
1264	30	MRS	aerobiosis
1456	30	MRS ph4.5	aerobiosis
1466	30	HJL	aerobiosis
1497	30	MRS	aerobiosis
1575	30	MRS	aerobiosis
1635	40	MRS	aerobiosis
1677	30	MRS	aerobiosis
1707	30	MRS	aerobiosis
1828	30	HJL	aerobiosis
1831	30	HJL	aerobiosis
2378	30	HJL	aerobiosis
2461	37	MRS	aerobiosis
2511	30	MRS	aerobiosis
2512	30	MHH	aerobiosis
2531	30	MRS	aerobiosis
2538	30	MRS	aerobiosis
2550	30	MHH	aerobiosis
2578	37	MRS CYS	anaeorobiosis
2603	40	MRS	aerobiosis
2607	30	MHH	aerobiosis
2651	37	MRS CYS	anaeorobiosis
2694	30	MHH	aerobiosis
2705	37	MRS CYS	anaeorobiosis
2733	30	MHH	aerobiosis
2768	37	BHI/HBL	aerobiosis
2795	40	HJL	aerobiosis
2818	37	MRS CYS	anaeorobiosis
2821	30	HJL	aerobiosis
2928	37	MRS	aerobiosis
2957	40	BST	aerobiosis
2971	40	BST	aerobiosis
2976	40	BST	aerobiosis
2979	40	BST	aerobiosis
2982	40	BST	aerobiosis
3059	40	MRS	aerobiosis
4007	37	MRS	aerobiosis
4032	40	BST	aerobiosis

Table S3

This table outlines the MS experiments performed during each cycle time for individual SWATH windows, detailing the acquisition parameters for both reverse-phase and HILIC separation methods. For the MS1 experiment, an acquisition time of 100 ms was employed, while subsequent

experiments utilized a 50 ms acquisition time. The upper section of the table specifies the SWATH windows used in the reverse-phase (RP) method, covering a mass range from 50 to 1500 Da. Conversely, the lower section details the SWATH windows for the Hydrophilic Interaction Liquid Chromatography (HILIC) method, which focuses on a narrower mass range from 50 to 1000 Da.

experiments REVERSED PHASE METHOD	MS Type	Min *m/z*	Max *m/z*
0	TOF SCAN	50	1500
1	SWATH	49.5	96
2	SWATH	95	131
3	SWATH	130	159.6
4	SWATH	158.6	190.1
5	SWATH	189.1	215.6
6	SWATH	214.6	243.6
7	SWATH	242.6	274.1
8	SWATH	273.1	303.6
9	SWATH	302.6	341.2
10	SWATH	340.2	374.6
11	SWATH	373.6	421.8
12	SWATH	420.8	453.2
13	SWATH	452.2	482.8
14	SWATH	481.8	513.8
15	SWATH	512.8	543.8
16	SWATH	542.8	593.1
17	SWATH	592.1	652.8
18	SWATH	651.8	705.3
19	SWATH	704.3	806.5
20	SWATH	805.5	1000.5
21	SWATH	999.5	1100.5
22	SWATH	1099.5	1200.5
23	SWATH	1199.5	1500
Experiments HILIC METHOD	**MS TYPE**	**MIN *M/Z***	**MAX *M/Z***
0	SCAN	49.5	1000
1	SWATH	49.5	135.5
2	SWATH	134.5	186
3	SWATH	185	222.5
4	SWATH	221.5	247.5
5	SWATH	246.5	267.5
6	SWATH	266.5	292.5
7	SWATH	291.5	315.5
8	SWATH	314.5	339.5
9	SWATH	338.5	365
10	SWATH	364	387.5

11	SWATH	386.5	421.5
12	SWATH	420.5	462.5
13	SWATH	461.5	493.5
14	SWATH	492.5	524
15	SWATH	523	562.5
16	SWATH	561.5	617.5
17	SWATH	616.5	669.5
18	SWATH	668.5	725.5
19	SWATH	724.5	815.5
20	SWATH	814.5	1000.5

Table S4

This table lists the reference standard compounds that were injected alongside the Round 3 samples, utilizing Hydrophilic Interaction Liquid Chromatography (HILIC) in separation mode and Sequential Window Acquisition of all Theoretical Mass Spectra (SWATH-MS) in Electrospray Ionization (ESI) negative mode. It includes amino acids and nucleosides reference standards, detailing their deprotonated experimental monoisotopic mass, the two principal fragments, and their retention times. These annotations facilitated the identification of compounds in the Round 3 samples from the HILIC negative mode data. The peak areas extracted from this analysis were subsequently used to generate the comparative heatmap presented in Figure 6C

**Standard reference compounds**	**M/Z $[M-H]^-$**	**MS/MS frag 1**	**MS/MS frag 2**	**Retention Time HILIC Method**
L-Arginine	173.1042	131.0881		9.12
L-Alanine	88.03877	88.0462	71.0188	8.556
L-Glyine	74.02455	59.0187	74.0308	7.706
L-Asparagine	131.04604	114.0252	70.03	8.084
L-Aspartic acid	132.03088	88.0462	115.0087	8.545
L-Glutamic acid	146.04631	102.059	128.0365	8.202
L-Glutamine	145.06216	127.0427	109.0409	7.934
L-Histidine	154.0624	93.0483	137.0354	8.911
L-Isoleucine (co elution with Isoleucine)	130.08707	130.091		5.804
L-Leucine (co elution with leucine)	130.08707	130.0941		5.929
L-Lysine	145.098666	145.1009	97.0816	9.308
L-Methionine	148.04378	148.0481	100.0464	6.185
L-Phenylalanine	164.07101	103.0451	147.0435	5.653
L-Proline	114.05584	114.0626	68.0551	6.441
L-Serine	104.03472	104.0336	74.0278	8.006
L-Threonine	118.05	74.0286		7.58
L-Tryptophan	203.08334	116.0574	142.0694	5.782
L-Tyrosine	180.06769	119.0535	163.0438	6.662
L-Valine	116.0719	116.0717		6.344
L-Pyroglutamic acid	128.03601	128.0269	82.0313	6.319

Adenosin 5'-monophosphat	346.05444	78.9591	134.0451	8.13
Adenosin 5'-diphosphat	426.0244	158.9245	78.9577	9.202
Adenosin 5'-triphosphat	505.98715	158.9279	408.0071	10
Cytidin 5'-monophosphat	322.04486	78.9635	322.042	8.71
Cytidin 5'-diphosphat	402.01007	158.92	78.9622	9.812
Cytidin 5'-triphosphat	481.9762	158.9281	383.996	10.34
Guanosin 5'-monophosphat	362.05136	78.9587	362.049	8.796
Guanosin 5'-diphosphat	442.01511	158.928	344.0406	10.03
Guanosin 5'-triphosphat	521.98364	424.0002	158.9284	10.39
Uridin 5'-monophosphat	323.02881	78.9637	211.0016	8.202
Uridin 5'-diphosphat	402.99744	158.9233	78.9573	9.383
Uridin 5'-triphosphat	na	na	na	na
Xanthosin 5'-monophosphat	363.03683	211.0063	151.0304	8.839
Inosin 5'-monophosphat	347.04129	78.9652	135.035	7.538
Thymidin 5'-monophosphat	321.04706	78.9582	125.0338	7.6
Adenosin 3':5'-monophosphat	328.04449	134.051	328.0425	6.861
Guanosin 3':5'-monophosphat	344.04053	150.0465	344.0376	7.524
Uridin 5'-diphosphoglucose	565.04437	196.06	291.0052	6.637
Uridin 5'-diphoshpoglucuronsäure	579.03485	402.9973	579.0162	9.984
Thymin	125.03663	125.0402	93.0515	1.778
Xanthosin	283.0674	151.0271	108.0134	5.658
Thymidin	241.0829	111.0166	233.1563	1.956
Cytosin	110.0346	67.0282	99.923	3.799
Uracil	111.01947	111.0231	65.9968	1.947
Uridin	243.06229	110.0234	152.0342	3.186
Inosin	267.07556	135.0338	108.0207	4.669
Xanthin	151.02673	108.0255	151.0302	3.552
Hypoxanthin	135.03165	92.0309	65.0189	3.044
Cytidin	242.07896	109.0392	81.0446	5.37
Guanosin	282.08447	150.0406	108.0148	6.012
Adenosin	266.09045	134.0465	107.0358	3.067
Guanin	150.04128	66.0084	133.013	5.072

Table S5

List of aroma compounds used during panellists' training (attributes familiarization). According to Utz et al., 2021 and 2022.

No.	Aroma compound used for training according to Utz et al., 2022	Odor Quality
1	3-methylbutanal	malty
2	hexanal	green, grassy
3	acetaldehyde	fresh, green
4	(*E,E*)-2,4-decadienal	fatty, deep-fried
5	phenylacetaldehyde	flowery, honey-like
6	(*E,E*)-2,4-nonadienal	fatty, green
7	(*E*)-2-octenal	fatty, nutty
8	diacetyl	butter-like
9	benzaldehyde	bitter almond-like, marzipan-like
10	heptanal	citrus-like, fatty
11	2-methylbutanal	malty
12	(*E*)-2-undecenal	soapy, metallic
13	nonanoic acid	moldy, pungent
14	methional	cooked potato-like
15	acetic acid	vinegar-like
16	3-methylbutanoic acid	sweaty
17	decanoic acid	soapy, musty
18	vanillin	vanilla-like, sweet
19	(*E,E*)-3,5-octadien-2-one	woody, mushroom-like, green
20	hexanoic acid	sweaty
21	octanoic acid	carrot-like, musty
22	phenylacetic acid	honey-like, beeswax-like
23	γ-octalactone	coconut-like
24	2-methylbutanoic acid	malty, fruity, cow's milk
25	2,3-octanedione	mushroom-like, dill-like, broccoli-like
26	2-undecanone	soapy, green
27	acetoin	butter-like, caramel-like

Table S6

Comprehensive analysis of bacterial strain viability, damage, and death overtime across various strains, including pH values, live (CFDA+), damaged (CFDA+/PI+), and dead (PI+) bacterial percentages, and total bacterial counts (TFUs/ml).

Strain NCC	Time (Hours)	pH value	% CFDA+ (Live)	%CFDA+/PI+ (Damaged)	% PI+ (Dead)	Total Bacteria (TFUs/ml)
41	0	7.18	16.8	8.6	74.6	9.34E+05
	24	6.5	86.8	0.6	12.6	7.85E+05
	48	6.41	66.4	2.2	31.4	1.08E+05
	72	6.5	21.2	0.0	78.8	4.85E+04
66	0	6.8	12.6	0.8	86.6	2.11E+05
	24	7.05	80.5	0.5	19.0	4.74E+05
	48	7.07	88.8	0.3	10.9	2.10E+05
	72	7.1	84.9	0.4	14.7	1.70E+05
82	0	7.2	85.8	1.7	12.4	2.51E+05
	24	7.2	85.6	1.4	13.0	3.70E+05
	48	7.14	97.0	0.3	2.7	2.91E+05
	72	7.2	86.7	0.8	12.5	1.83E+05
251	0	6.8	21.4	37.4	41.2	9.91E+05
	24	5.9	16.9	30.5	52.6	5.30E+05
	48	5.62	35.7	15.1	49.2	4.11E+05
	72	5.53	38.3	14.3	47.3	7.53E+05
273	0	7	84.9	1.3	13.7	9.23E+05
	24	5.94	29.5	61.1	9.4	1.38E+06
	48	5.67	55.9	35.2	8.8	2.15E+06
	72	5.48	51.8	39.3	9.0	2.46E+06
283	0	6.83	36.8	9.4	53.8	5.07E+05
	24	5.83	5.8	17.0	77.2	7.01E+05
	48	5.66	17.2	24.2	58.6	2.05E+06
	72	5.58	28.8	29.6	41.6	1.95E+06
325	0	7.11	33.8	34.7	31.5	9.94E+05
	24	6.44	31.1	23.8	45.1	3.46E+05
	48	6.44	28.9	11.6	59.5	4.18E+05
	72	6.44	27.7	6.0	66.4	4.06E+05
362	0	6.96	61.7	11.5	26.8	1.61E+06
	24	5.37	56.0	18.7	25.3	8.01E+05
	48	5.3	72.7	5.3	22.0	6.54E+05
	72	5.41	63.9	7.2	28.9	1.31E+06
364	0	7.07	47.8	13.3	38.9	4.25E+05
	24	6.58	46.8	6.0	47.2	7.98E+05
	48	6.5	33.5	1.7	64.8	1.09E+06

	72	6.42	34.0	1.3	64.7	6.58E+05
420	0	7.05	21.7	26.4	51.8	4.65E+05
	24	6.96	42.3	6.4	51.4	7.74E+05
	48	6.96	35.9	8.4	55.7	4.82E+05
	72	6.96	50.9	7.3	41.8	5.15E+05
424	0	6.98	43.7	13.1	43.2	7.27E+05
	24	5.41	17.2	34.5	48.3	2.50E+06
	48	5.42	19.6	31.7	48.7	1.61E+06
	72	5.4	29.8	24.9	45.4	3.12E+06
445	0	7.03	73.3	4.4	22.2	4.45E+05
	24	6.92	69.5	0.4	30.0	1.05E+06
	48	6.55	64.9	3.2	31.9	4.10E+05
	72	6.42	65.8	2.6	31.6	4.08E+05
472	0	6.7	22.1	1.0	76.9	9.09E+04
	24	7.18	17.6	1.4	81.1	1.47E+05
	48	7.1	21.9	1.0	77.1	1.27E+05
	72	7.16	52.5	3.4	44.1	1.65E+05
515	0	7.18	66.9	0.4	32.6	4.36E+05
	24	6.84	70.4	0.0	29.6	3.11E+05
	48	6.82	82.9	0.0	17.1	5.31E+05
	72	6.86	49.6	0.8	49.6	4.63E+05
525	0	6.95	8.9	0.2	90.8	2.04E+06
	24	6.96	15.1	9.4	75.6	3.34E+06
	48	6.97	13.9	1.2	84.9	1.07E+06
	72	6.95	11.2	3.9	84.9	1.01E+06
533	0	6.6	26.1	0.8	73.1	1.33E+06
	24	6.08	51.8	14.3	33.9	2.99E+06
	48	5.94	16.8	10.8	72.4	6.36E+05
	72	5.728	21.6	12.2	66.2	7.08E+05
542	0	6.9	63.7	0.0	36.3	1.21E+05
	24	6.58	70.2	15.8	14.0	1.51E+06
	48	6.33	82.1	2.1	15.8	1.49E+06
	72	6.58	96.3	0.1	3.6	1.47E+06
660	0	7.22	84.6	0.0	15.4	2.62E+05
	24	6.5	85.6	1.4	12.9	5.04E+05
	48	6.39	50.3	8.2	41.5	9.97E+05
	72	6.42	73.7	6.1	20.2	2.24E+05
692	0	6.91	84.4	0.0	15.6	1.91E+05
	24	6.46	92.7	0.0	7.3	7.23E+05
	48	6.31	60.4	4.2	35.4	1.39E+05
	72	6.35	75.4	2.5	22.1	3.13E+05
729	0	7.11	35.8	0.9	63.2	1.75E+05
	24	6.53	84.1	0.4	15.5	1.18E+06
	48	6.49	81.7	0.9	17.5	5.21E+05
	72	6.55	76.0	0.5	23.5	3.05E+05
743	0	7.12	49.9	8.4	41.7	4.45E+05

	24	6.51	73.4	1.0	25.6	5.93E+05
	48	6.38	81.8	0.8	17.5	2.10E+05
	72	6.42	68.8	1.5	29.7	2.04E+05
827	0	7.25	61.9	1.4	36.7	1.51E+05
	24	7.2	76.9	0.4	22.7	1.46E+05
	48	7.23	70.6	0.0	29.4	1.50E+05
	72	7.24	70.4	1.2	28.5	2.37E+05
913	0	7.27	68.3	3.2	28.5	1.04E+05
	24	7.26	85.1	3.3	11.6	3.22E+05
	48	7.26	86.6	2.4	11.1	3.13E+05
	72	7.21	64.6	1.8	33.7	2.55E+05
971	0	7.26	82.5	0.3	17.2	3.72E+05
	24	7.48	98.2	0.1	1.7	6.23E+06
	48	7.8	97.4	0.2	2.3	9.09E+06
	72	8.04	95.2	0.9	3.9	1.04E+07
1034	0	7.23	91.4	0.6	8.0	3.39E+05
	24	7.42	99.4	0.2	0.4	1.15E+07
	48	7.74	95.9	0.2	3.9	2.23E+06
	72	7.99	93.4	0.9	5.7	3.41E+06
1039	0	7.05	86.1	0.2	13.8	5.53E+05
	24	6.1	33.5	3.5	63.0	2.03E+05
	48	6.13	32.4	12.4	55.2	1.38E+05
	72	6.18	48.1	4.5	47.5	8.47E+04
1061	0	7.18	81.3	0.6	18.1	6.59E+05
	24	7.71	88.4	2.5	9.1	9.39E+05
	48	7.93	97.9	0.5	1.7	1.31E+07
	72	8.074	96.3	2.1	1.6	1.16E+07
1077	0	7.2	84.6	3.2	12.2	2.70E+06
	24	7.18	57.2	0.0	42.8	3.98E+05
	48	7.24	56.2	0.3	43.5	3.14E+05
	72	7.32	61.3	1.1	37.7	4.86E+05
1094	0	6.8	86.1	2.7	11.2	1.42E+06
	24	6.11	83.5	1.9	14.6	3.67E+05
	48	6.14	96.7	0.2	3.1	2.87E+05
	72	6.19	92.1	0.2	7.7	2.65E+05
1138	0	7.16	82.5	1.0	16.4	3.19E+05
	24	7.24	87.3	0.5	12.2	4.10E+05
	48	7.15	62.2	0.8	37.0	1.68E+05
	72	7.26	61.5	2.9	35.6	2.95E+05
1144	0	na	39.0	0.5	60.6	4.78E+05
	24	na	60.8	1.7	37.6	3.74E+04
	48	na	72.3	0.0	27.7	3.37E+04
	72	na	69.1	0.0	30.9	5.60E+04
1159	0	7.2	78.7	0.0	21.3	4.21E+05
	24	7.22	69.1	0.4	30.5	3.18E+05
	48	7.08	54.6	0.4	45.0	2.26E+05

	72	7.23	36.4	0.7	62.9	1.48E+05
1177	0	7.18	69.1	0.0	30.9	1.84E+05
	24	7.16	18.7	0.0	81.3	2.07E+05
	48	7.11	39.4	1.8	58.8	3.57E+05
	72	7.11	16.9	0.0	83.1	1.32E+05
1222	0	6.95	18.8	9.5	71.7	2.40E+06
	24	5.74	22.5	10.5	67.1	1.84E+05
	48	5.8	12.6	3.5	83.9	6.77E+05
	72	5.83	12.2	2.2	85.6	7.88E+05
1230	0	7.22	62.5	23.1	14.4	9.34E+05
	24	7.28	86.8	1.0	12.2	3.48E+05
	48	7.18	71.3	1.6	27.1	4.07E+05
	72	7.18	68.8	0.2	31.0	3.41E+05
1236	0	7.18	65.4	0.6	34.1	2.29E+05
	24	7.21	31.4	0.0	68.6	4.77E+05
	48	7.11	23.9	0.8	75.3	5.42E+05
	72	7.12	27.5	0.9	71.6	6.26E+05
1240	0	6.95	14.2	0.0	85.8	2.24E+05
	24	6.2	40.1	0.0	59.9	3.44E+05
	48	6.15	13.8	0.0	86.2	1.60E+05
	72	6.18	54.1	1.5	44.4	1.58E+05
1264	0	6.93	6.7	0.0	93.3	2.70E+05
	24	6.29	95.2	0.7	4.1	3.91E+05
	48	5.87	92.4	2.0	5.6	6.13E+05
	72	5.5	94.5	1.6	3.9	3.80E+05
1456	0	7.09	47.2	0.0	52.8	3.08E+04
	24	7.16	35.9	1.0	63.1	6.42E+04
	48	7.07	43.7	0.0	56.3	7.29E+04
	72	7.09	64.3	1.3	34.4	1.12E+05
1497	0	7.06	na	na	na	na
	24	6.97	55.8	18.1	26.1	7.36E+05
	48	6.86	68.1	7.8	24.1	6.80E+05
	72	6.91	37.0	5.4	57.6	4.85E+05
1575	0	6.93	33.9	0.6	65.5	1.10E+06
	24	6.94	na	na	na	na
	48	6.92	na	na	na	na
	72	6.94	29.4	1.7	68.9	7.29E+05
1635	0	6.87	12.4	0.0	87.6	1.30E+06
	24	6.71	73.3	2.4	24.4	3.27E+05
	48	6.7	57.1	10.5	32.3	4.59E+05
	72	6.65	65.5	5.0	29.4	2.33E+05
1677	0	6.96	58.2	0.0	41.8	2.90E+05
	24	6.35	58.6	1.0	40.4	8.06E+05
	48	6.1	71.3	0.9	27.9	1.08E+05
	72	5.95	43.6	2.1	54.3	2.79E+05
1707	0	6.55	87.5	1.5	11.0	2.08E+06

	24	6.18	77.4	2.3	20.2	7.44E+05
	48	6.25	91.0	0.9	8.0	8.94E+05
	72	6.19	92.1	0.3	7.6	1.54E+06
2378	0	7.2	36.9	4.1	59.0	4.56E+05
	24	6.3	89.4	4.5	6.1	1.69E+07
	48	6.36	90.0	6.9	3.0	3.81E+06
	72	6.57	94.2	2.3	3.5	1.08E+07
2461	0	7.08	na	na	na	na
	24	7.01	97.3	0.9	1.8	1.49E+07
	48	6.96	28.0	2.9	69.1	3.36E+05
	72	6.93	37.5	2.5	60.0	9.38E+05
2511	0	6.93	74.5	1.7	23.7	9.46E+05
	24	7	43.4	5.4	51.2	2.99E+06
	48	6.98	82.0	2.9	15.1	1.38E+06
	72	6.95	55.6	5.0	39.4	1.07E+06
2512	0	6.9	67.8	4.1	28.2	4.52E+05
	24	7.04	63.6	2.4	34.0	6.61E+05
	48	6.93	83.6	1.9	14.5	4.72E+05
	72	6.94	79.8	2.3	17.8	2.91E+05
2531	0	7.1	38.2	0.0	61.8	3.29E+05
	24	7.02	51.5	1.4	47.0	8.79E+05
	48	6.96	67.8	2.1	30.0	5.61E+05
	72	7.007	47.1	0.0	52.9	3.32E+05
2538	0	7.07	27.2	0.4	72.5	6.45E+05
	24	6.54	73.3	3.2	23.5	1.48E+06
	48	6.55	85.8	1.9	12.3	1.02E+06
	72	6.55	75.0	0.6	24.4	5.82E+05
2550	0	7.11	57.5	11.5	31.0	2.83E+05
	24	6.86	37.8	5.5	56.6	1.87E+05
	48	6.94	81.7	0.0	18.3	1.50E+05
	72	6.91	65.8	0.5	33.7	1.86E+05
2578	0	7.15	77.4	9.3	13.3	1.26E+06
	24	5.65	92.3	6.9	0.7	8.32E+06
	48	5.61	96.6	2.4	1.0	9.48E+06
	72	5.58	88.7	2.6	8.7	6.36E+06
2603	0	7.09	9.7	2.4	87.8	4.02E+05
	24	7.1	60.7	20.3	19.0	1.67E+06
	48	6.34	86.2	10.9	2.9	2.84E+06
	72	6.12	91.2	4.8	4.0	3.64E+06
2607	0	6.77	78.0	5.8	16.1	3.28E+05
	24	6.92	79.6	3.9	16.4	3.15E+05
	48	6.89	82.5	0.3	17.1	1.49E+05
	72	6.88	60.9	2.3	36.8	1.65E+05
2651	0	7.02	67.3	14.1	18.6	6.04E+05
	24	5.37	72.4	12.7	14.9	1.48E+06
	48	5.4	69.0	25.2	5.9	3.18E+06

	72	5.38	69.1	24.5	6.3	2.47E+06
2694	0	7	83.9	5.6	10.5	1.88E+05
	24	6.85	79.3	1.8	18.9	2.49E+05
	48	6.81	80.1	0.0	19.9	1.06E+05
	72	6.89	71.0	1.8	27.2	2.12E+05
2705	0	6.85	67.1	19.0	13.8	4.99E+05
	24	6.3	17.6	43.4	39.1	4.16E+05
	48	6.27	55.0	5.9	39.1	1.74E+06
	72	6	25.4	15.3	59.3	6.01E+05
2733	0	7.11	72.4	9.3	18.3	5.83E+05
	24	6.89	63.5	7.1	29.4	2.42E+05
	48	6.92	49.1	0.3	50.6	3.57E+05
	72	6.86	51.4	1.4	47.3	2.97E+05
2768	0	7.2	73.1	1.9	24.9	3.15E+05
	24	6.68	na	na	na	na
	48	6.64	84.8	0.5	14.7	3.42E+05
	72	6.5	na	na	na	na
2795	0	7.11	60.7	0.4	38.9	5.70E+05
	24	6.38	72.4	11.8	15.8	8.33E+05
	48	6.59	43.1	22.2	34.7	4.63E+05
	72	6.55	42.4	24.3	33.3	5.76E+05
2818	0	6.82	87.7	4.9	7.3	6.52E+06
	24	5.43	77.3	12.9	9.8	4.21E+05
	48	5.4	90.2	3.1	6.8	2.68E+05
	72	5.42	89.9	3.8	6.3	1.57E+06
2928	0	6.74	14.9	1.9	83.2	9.21E+05
	24	6.19	47.6	9.0	43.5	2.14E+06
	48	5.88	18.6	7.6	73.9	5.42E+05
	72	5.85	8.4	2.1	89.5	2.29E+05
2971	0	7.23	88.7	1.0	10.3	1.52E+05
	24	6.72	99.3	0.2	0.5	2.32E+06
	48	7.22	39.3	22.2	38.4	1.04E+06
	72	6.92	17.0	13.3	69.7	3.34E+06
2957	0	7.25	68.7	4.8	26.5	7.25E+05
	24	6.78	9.7	17.2	73.1	1.68E+06
	48	7.16	86.3	5.1	8.7	1.35E+06
	72	7.73	na	na	na	na
2976	0	7.23	na	na	na	na
	24	6.67	82.4	12.0	5.6	6.36E+06
	48	7.07	69.9	23.8	6.3	5.93E+06
	72	7.48	71.5	13.0	15.5	6.31E+06
2979	0	7.51	87.8	0.5	11.7	3.51E+05
	24	6.87	43.0	21.4	35.6	1.77E+06
	48	6.92	98.2	1.1	0.7	8.12E+06
	72	8.37	45.4	10.3	44.3	2.96E+06
2982	0	7.38	89.2	1.3	9.5	5.58E+05

	24	6.78	76.4	15.2	8.4	6.36E+06
	48	7.2	92.0	5.8	2.2	5.09E+06
	72	7.54	55.6	25.4	19.0	8.07E+06
3059	0	7.19	25.9	0.7	73.4	3.02E+05
	24	6.63	46.6	12.5	40.9	4.42E+05
	48	6.62	72.7	8.5	18.8	3.01E+05
	72	6.61	63.2	14.0	22.7	4.15E+05
4007	0	6.85	2.8	0.4	96.8	4.88E+05
	24	6.91	27.8	11.8	60.4	1.04E+06
	48	6.98	11.2	14.4	74.4	5.27E+05
	72	6.97	2.9	1.2	95.9	8.84E+05
4032	0	7.27	72.6	4.6	22.8	7.46E+05
	24	6.75	22.7	13.9	63.4	1.50E+06
	48	7.37	19.3	10.5	70.2	3.35E+05
	72	7.98	45.3	9.7	45.0	9.71E+05

Table S7

Summary of Untargeted LC-TOF-MS Feature Analysis obtained from the alignment file of round 1. This table presents the detailed characteristics of features identified through LC-TOF-MS alignment. 'Feature ID' corresponds to the unique alignment identifier for each deconvoluted and aligned signal. 'Retention Time (min)' denotes the elution time in minutes on the C18 column employed during the first round. 'Precursor m/z' indicates the deprotonated (Neg ionization mode) monoisotopic mass of the precursor ion. 'Predicted Formula' is derived using the MSFINDER formula calculator. 'Mass Error (mDa)' reflects the deviation between the exact and experimental mass. 'Rank 1 Structure' represents the most plausible structure as determined by the MSFINDER annotation tool.

Feature ID	RT (C18 column)	precursor m/z	precursor type	Formula	Error [mDa]	Structure rank 1 (+ MSFINDER score)	Reference standard
5022	9	1027.49856	[M-H]-	C51H8O21	13.37	Pisumsaponin I	NA
4799	10.24	941.51154	[M-H]-	C48H78O18	-0.0008	Soyasaponin I	NA
4798	10.69	939.4987	[M-H]-	C48H76O18	-2.9675	Dehydrosoyasaponin I	NA
4758	10.58	925.51994	[M-H]-	C48H78O17	-3.5154	Triterpene saponins (unknown ID)	NA
685	13.15	295.23027	[M-H]-	C18H32O3	-2.8516	13-Hydroxyoctadeca-9,11-dienoic Acid	Matched with coelution

683	14.7	295.23072	[M-H]-	C18H32O3	-2.4016	9-Hydroxyoctadeca-9,11-dienoic Acid	Matched
804	11.55	313.23492	[M-H]-	C18H34O4	3.5131	9,10-DHOME	Matched
930	9.15	329.23482	[M-H]-	C18H34O5	-1.3123	9,10,11-TriHOME	Matched
578	16.36	279.23124	[M-H]-	C18H32O2	1.7138	Linoleic acid	Matched
805	11.6821	313.2378	[M-H]-	C18H34O4	11.6821	12,13-DHOME	Matched
5026	9.8	1029.52504	[M-H]-	C51H82O21	2.57432	Steroidal saponins (unknown ID)	NA
931	10.15	329.23172	[M-H]-	C18H34O5	-1.4723	11,12,13-TriHOME	NA
5097	10.8867	1067.5469	[M-H]-	C54H84O21	-3.6567	Soyasaponin Bg	NA
931	10.15	329.23172	[M-H]-	C18H34O5	1.6277	9,12,13-TriHOME	Matched
925	9.88	329.23172	[M-H]-	C18H34O5	1.6277	9,10,13-TriHOME	Matched

Table S8

Continuation of table S7. Feature ID as described in the table.

Feature ID	InChKey	Ontology	MS/MS (Relative abudance)
5022	UZZVCPOZXWRBFO-UHFFFAOYNA-N	Triterpene saponins	1027.51 (100), 983.52 (65)
4798	PTDAHAWQAGSZDD-UHFFFAOYNA-N	Triterpene saponins	941.51 (100), 615.39 (5), 205.0707 (3)
4798	CROUPKILZUPLQA-UHFFFAOYNA-N	Triterpene saponins	939.5006 (100), 613.3773 (2)
685	NPDSHTNEKLQQIJ-UINYOVNOSA-N	Lineolic acids and derivatives	277.21 (100), 195.14 (100), 57.03 (100)
683	HNICUWMFWZBIFP-IRQZEAMPSA-N	Lineolic acids and derivatives	277.20 (40), 247.20 (40), 123.08 (40), 69.03 (60), 55.01 (100)
804	XEBKSQSGNGRGDW-YFHOEESVNA-N	Long-chain fatty acids	177.09 (90), 127.10 (100)
930	MDIUMSLCYIJBQC-MVFSOIOZSA-N	Long-chain fatty acids	293.21 (10), 229.1432 (20), 201.11 (100), 171.10 (70), 139.11 (30), 99.08 (30), 71 (5)
578	OYHQOLUKZRVURQ-HZJYTTRNSA-N	Lineolic acids and derivatives	297.2397 (95), 183.01 (75), 57.03 (39)
805	CQSLTKIXAJTQGA-FLIBITNWNA-N	Long-chain fatty acids	171.10 (70), 127.10 (100)
5026		Steroidal saponins	985.5379 (100), 205.0716 (10)

931	NTVFQBIHLSPEGQ-SYMVGPSASA-N	Long-chain fatty acids	129.09 (50), 199.13 (40), 221.12 (50), 211.13 (30)
5097	ONAAMCDHQSWPDU-UHFFFAOYNA-N	Triterpene saponins	1067.54 (100), 163.0594 (10)
931	MDIUMSLCYIJBQC-MVFSOIOZSA-N	Long-chain fatty acids	329.22 (40), 229.14 (40), 211.13 (50), 68.0122 (100)
925	NTVFQBIHLSPEGQ-SYMVGPSASA-N	Long-chain fatty acids	329.23 (100), 233.12 (90), 211.13 (100)

Table S9

Viability and Concentration of the 17 Bacterial Strains employed in Round 2 Post-48h-Fermentation. This table displays the viability percentages of various bacterial strains, categorized as live (% CFDA+), damaged (% CFDA+/PI+), and dead (% PI+), following a standard fermentation process. Additionally, the total bacterial count is quantified as total fluorescent units per milliliter (TFUs/ml).

NCC strain	Species	% CFDA+ (Live)	% CFDA+/PI+ (Damaged)	% PI+ (Dead)	Total Bacteria (TFUs/ml)
156	*Bacillus amyloliquefaciens*	45.8	19.3	34.9	2.76E+06
283	*Bifidobacterium longum*	54.9	9.9	35.2	1.93E+06
525	*Lacticaseibacillus rhamnosus*	62.7	7.3	30	1.44E+06
533	*Lactobacillus johnsonii*	30.3	18.6	51.1	1.95E+06
660	*Limosilactobacillus fermentum*	91.6	2.1	6.3	2.93E+06
1061	*Staphylococcus carnosus*	87.6	8	4.4	1.03E+07
1177	*Propionibacterium freudenreichii*	14.9	10.1	75.1	1.09E+06
1240	*Lactiplantibacillus plantarum*	52.4	3.1	44.4	4.18E+05
2378	*Lactococcus lactis*	94.2	3.3	2.5	1.05E+07
2511	*Lacticaseibacillus paracasei*	86.6	3.3	10.1	3.64E+06
2705	*Bifidobacterium longum*	49.5	36.6	14	1.77E+06
2795	*Streptococcus thermophilus*	88.7	2.8	8.5	1.86E+06

2957	*Bacillus subtilis*	9.8	18.4	71.7	1.06E+07
2976	*Bacillus subtilis*	41.6	26.6	31.8	1.10E+07
2982	*Bacillus subtilis*	37.9	12.2	49.8	3.79E+05
3059	*Limosilactobacillus fermentum*	12.4	19.3	68.3	2.97E+06
4007	*Lacticaseibacillus rhamnosus*	49.3	4.2	46.5	7.73E+05

Table S10

This table report the sensory data from round 2 depicted in Figure 3, for the taste attributes specifically. For each fermented sample tested and for each attribute evaluated this table report the average intensity “intensity”, the standard deviation, the standard error of the mean (se) and the related confidence intervals. The columns Intensity change is reporting the respective change in intensiy (%) compared to the reference unfermented score value.

Attribute	TREATMENT	NCC	SAMPLE	Intensity	sd	se	ci	Intensity Change (%)
TS-ASTRINGENT	FERMENTED	1061	NCC 1061	2.785714	1.409998	0.532929	1.304031	-7.14286
TS-ASTRINGENT	FERMENTED	1177	NCC 1177	2.028571	1.007827	0.380923	0.932084	-32.381
TS-ASTRINGENT	FERMENTED	1240	NCC 1240	2.457143	1.1458	0.433072	1.059688	-18.0952
TS-ASTRINGENT	FERMENTED	156	NCC 156	3.171429	1.501904	0.567666	1.389029	5.714286
TS-ASTRINGENT	FERMENTED	2378	NCC 2378	1.9375	0.903861	0.319563	0.755647	-35.4167
TS-ASTRINGENT	FERMENTED	2511	NCC 2511	2.6125	0.823646	0.291203	0.688585	-12.9167
TS-ASTRINGENT	FERMENTED	2705	NCC 2705	3.5	0.559017	0.186339	0.429699	16.66667
TS-ASTRINGENT	FERMENTED	2795	NCC 2795	2.333333	0.829156	0.276385	0.637346	-22.2222
TS-ASTRINGENT	FERMENTED	283	NCC 283	2.833333	0.829156	0.276385	0.637346	-5.55556
TS-ASTRINGENT	FERMENTED	2957	NCC 2957	3.166667	0.829156	0.276385	0.637346	5.555556
TS-ASTRINGENT	FERMENTED	2976	NCC 2976	3.022222	0.983333	0.327778	0.755857	0.740741
TS-ASTRINGENT	FERMENTED	3059	NCC 3059	2.111111	0.650854	0.216951	0.500291	-29.6296
TS-ASTRINGENT	FERMENTED	4007	NCC 4007	2.777778	0.794949	0.264983	0.611052	-7.40741
TS-ASTRINGENT	FERMENTED	525	NCC 525	2.4	0.526783	0.175594	0.404921	-20
TS-ASTRINGENT	FERMENTED	533	NCC 533	2.277778	0.794949	0.264983	0.611052	-24.0741
TS-ASTRINGENT	FERMENTED	660	NCC 660	2.1	0.991211	0.330404	0.761913	-30
TS-ASTRINGENT	UNFERMENTED	UNFERMENTED	UNFERMENTED	3	NA	NA	NA	0
TS-BITTER	FERMENTED	1061	NCC 1061	3.1	1.042433	0.394003	0.96409	24
TS-BITTER	FERMENTED	1177	NCC 1177	2.157143	0.269921	0.10202	0.249635	-13.7143
TS-BITTER	FERMENTED	1240	NCC 1240	2.342857	0.556349	0.21028	0.514537	-6.28571
TS-BITTER	FERMENTED	156	NCC 156	2.785714	0.755929	0.285714	0.699118	11.42857
TS-BITTER	FERMENTED	2378	NCC 2378	1.7125	0.888719	0.31421	0.742988	-31.5
TS-BITTER	FERMENTED	2511	NCC 2511	2.2125	0.653425	0.231021	0.546277	-11.5
TS-BITTER	FERMENTED	2705	NCC 2705	2.333333	1.089725	0.363242	0.837637	-6.66667
TS-BITTER	FERMENTED	2795	NCC 2795	2.344444	0.450309	0.150103	0.346138	-6.22222
TS-BITTER	FERMENTED	283	NCC 283	2.188889	0.45674	0.152247	0.351081	-12.4444
TS-BITTER	FERMENTED	2957	NCC 2957	3.477778	0.701388	0.233796	0.539134	39.11111
TS-BITTER	FERMENTED	2976	NCC 2976	3.255556	0.60231	0.20077	0.462977	30.22222
TS-BITTER	FERMENTED	3059	NCC 3059	2.177778	0.5696	0.189867	0.437834	-12.8889
TS-BITTER	FERMENTED	4007	NCC 4007	2.055556	1.210487	0.403496	0.930462	-17.7778
TS-BITTER	FERMENTED	525	NCC 525	1.744444	0.72476	0.241587	0.5571	-30.2222
TS-BITTER	FERMENTED	533	NCC 533	1.666667	0.559017	0.186339	0.429699	-33.3333
TS-BITTER	FERMENTED	660	NCC 660	1.844444	0.963212	0.321071	0.74039	-26.2222
TS-BITTER	UNFERMENTED	UNFERMENTED	UNFERMENTED	2.5	NA	NA	NA	0
TS-SALTY	FERMENTED	1061	NCC 1061	0.642857	0.198806	0.075142	0.183865	-8.16327
TS-SALTY	FERMENTED	1177	NCC 1177	0.728571	0.075593	0.028571	0.069912	4.081633
TS-SALTY	FERMENTED	1240	NCC 1240	0.7	0	0	0	0
TS-SALTY	FERMENTED	156	NCC 156	0.714286	0.037796	0.014286	0.034956	2.040816

TS-SALTY	FERMENTED	2378	NCC 2378	0.7	0	0	0	0
TS-SALTY	FERMENTED	2511	NCC 2511	0.725	0.070711	0.025	0.059116	3.571429
TS-SALTY	FERMENTED	2705	NCC 2705	0.588889	0.202759	0.067586	0.155854	-15.873
TS-SALTY	FERMENTED	2795	NCC 2795	0.711111	0.033333	0.011111	0.025622	1.587302
TS-SALTY	FERMENTED	283	NCC 283	0.7	0	0	0	0
TS-SALTY	FERMENTED	2957	NCC 2957	0.7	0	0	0	0
TS-SALTY	FERMENTED	2976	NCC 2976	0.688889	0.078174	0.026058	0.06009	-1.5873
TS-SALTY	FERMENTED	3059	NCC 3059	0.688889	0.033333	0.011111	0.025622	-1.5873
TS-SALTY	FERMENTED	4007	NCC 4007	0.677778	0.120185	0.040062	0.092382	-3.1746
TS-SALTY	FERMENTED	525	NCC 525	0.677778	0.083333	0.027778	0.064056	-3.1746
TS-SALTY	FERMENTED	533	NCC 533	0.977778	0.222361	0.07412	0.170922	39.68254
TS-SALTY	FERMENTED	660	NCC 660	0.811111	0.252212	0.084071	0.193868	15.87302
TS-SALTY	UNFERMENTED	UNFERMENTED	UNFERMENTED	0.7	NA	NA	NA	0
TS-SOUR	FERMENTED	1061	NCC 1061	0.371429	0.262769	0.099317	0.243021	-38.0952
TS-SOUR	FERMENTED	1177	NCC 1177	0.642857	0.113389	0.042857	0.104868	7.142857
TS-SOUR	FERMENTED	1240	NCC 1240	0.571429	0.34983	0.132223	0.323539	-4.7619
TS-SOUR	FERMENTED	156	NCC 156	0.671429	0.249762	0.094401	0.230991	11.90476
TS-SOUR	FERMENTED	2378	NCC 2378	0.5125	0.299702	0.105961	0.250557	-14.5833
TS-SOUR	FERMENTED	2511	NCC 2511	0.9125	0.513914	0.181696	0.429642	52.08333
TS-SOUR	FERMENTED	2705	NCC 2705	1.066667	1.11018	0.37006	0.85336	77.77778
TS-SOUR	FERMENTED	2795	NCC 2795	0.511111	0.116667	0.038889	0.089678	-14.8148
TS-SOUR	FERMENTED	283	NCC 283	0.611111	0.419656	0.139885	0.322576	1.851852
TS-SOUR	FERMENTED	2957	NCC 2957	0.711111	0.297676	0.099225	0.228814	18.51852
TS-SOUR	FERMENTED	2976	NCC 2976	0.511111	0.136423	0.045474	0.104864	-14.8148
TS-SOUR	FERMENTED	3059	NCC 3059	0.944444	0.961914	0.320638	0.739392	57.40741
TS-SOUR	FERMENTED	4007	NCC 4007	1.055556	1.300107	0.433369	0.999351	75.92593
TS-SOUR	FERMENTED	525	NCC 525	0.811111	0.548736	0.182912	0.421796	35.18519
TS-SOUR	FERMENTED	533	NCC 533	0.666667	0.482183	0.160728	0.370638	11.11111
TS-SOUR	FERMENTED	660	NCC 660	0.555556	0.101379	0.033793	0.077927	-7.40741
TS-SOUR	UNFERMENTED	UNFERMENTED	UNFERMENTED	0.6	NA	NA	NA	0
TS-SWEET	FERMENTED	1061	NCC 1061	0.357143	0.315474	0.119238	0.291765	-48.9796
TS-SWEET	FERMENTED	1177	NCC 1177	0.657143	0.161835	0.061168	0.149672	-6.12245
TS-SWEET	FERMENTED	1240	NCC 1240	0.685714	0.106904	0.040406	0.09887	-2.04082
TS-SWEET	FERMENTED	156	NCC 156	0.5	0.223607	0.084515	0.206802	-28.5714
TS-SWEET	FERMENTED	2378	NCC 2378	0.7125	0.379614	0.134214	0.317366	1.785714
TS-SWEET	FERMENTED	2511	NCC 2511	0.6375	0.206588	0.07304	0.172712	-8.92857
TS-SWEET	FERMENTED	2705	NCC 2705	0.611111	0.428499	0.142833	0.329373	-12.6984
TS-SWEET	FERMENTED	2795	NCC 2795	0.711111	0.208833	0.069611	0.160523	1.587302
TS-SWEET	FERMENTED	283	NCC 283	0.844444	0.579032	0.193011	0.445083	20.63492
TS-SWEET	FERMENTED	2957	NCC 2957	0.133333	0.217945	0.072648	0.167527	-80.9524
TS-SWEET	FERMENTED	2976	NCC 2976	0.477778	0.258736	0.086245	0.198882	-31.746
TS-SWEET	FERMENTED	3059	NCC 3059	0.811111	0.50111	0.167037	0.385187	15.87302
TS-SWEET	FERMENTED	4007	NCC 4007	0.988889	0.592546	0.197515	0.455471	41.26984
TS-SWEET	FERMENTED	525	NCC 525	0.711111	0.252212	0.084071	0.193868	1.587302
TS-SWEET	FERMENTED	533	NCC 533	0.955556	0.35746	0.119153	0.274768	36.50794
TS-SWEET	FERMENTED	660	NCC 660	0.722222	0.349205	0.116402	0.268423	3.174603

TS-SWEET	UNFERMENTED	UNFERMENTED	UNFERMENTED	0.7	NA	NA	NA	0
TS-UMAMI	FERMENTED	1061	NCC 1061	0.614286	0.533631	0.201694	0.493526	2.380952
TS-UMAMI	FERMENTED	1177	NCC 1177	0.642857	0.113389	0.042857	0.104868	7.142857
TS-UMAMI	FERMENTED	1240	NCC 1240	0.685714	0.106904	0.040406	0.09887	14.28571
TS-UMAMI	FERMENTED	156	NCC 156	0.557143	0.053452	0.020203	0.049435	-7.14286
TS-UMAMI	FERMENTED	2378	NCC 2378	0.5375	0.192261	0.067975	0.160734	-10.4167
TS-UMAMI	FERMENTED	2511	NCC 2511	0.575	0.138873	0.049099	0.116101	-4.16667
TS-UMAMI	FERMENTED	2705	NCC 2705	0.722222	0.227913	0.075971	0.17519	20.37037
TS-UMAMI	FERMENTED	2795	NCC 2795	0.577778	0.130171	0.04339	0.100058	-3.7037
TS-UMAMI	FERMENTED	283	NCC 283	0.677778	0.323179	0.107726	0.248417	12.96296
TS-UMAMI	FERMENTED	2957	NCC 2957	0.711111	0.503598	0.167866	0.3871	18.51852
TS-UMAMI	FERMENTED	2976	NCC 2976	0.644444	0.350397	0.116799	0.269339	7.407407
TS-UMAMI	FERMENTED	3059	NCC 3059	0.622222	0.109291	0.03643	0.084008	3.703704
TS-UMAMI	FERMENTED	4007	NCC 4007	0.666667	0.173205	0.057735	0.133137	11.11111
TS-UMAMI	FERMENTED	525	NCC 525	0.622222	0.192209	0.06407	0.147745	3.703704
TS-UMAMI	FERMENTED	533	NCC 533	1.544444	0.32059	0.106863	0.246427	157.4074
TS-UMAMI	FERMENTED	660	NCC 660	1.122222	0.286259	0.09542	0.220038	87.03704
TS-UMAMI	UNFERMENTED	UNFERMENTED	UNFERMENTED	0.6	NA	NA	NA	0

Table S11

This table report the sensory data from round 2 depicted in Figure 5, for the aroma attributes specifically. For each fermented sample tested and for each attribute evaluated this table report the average intensity “intensity”, the standard deviation, the standard error of the mean (se) and the related confidence intervals.

Attribute	TREATMENT	NCC	SAMPLE	Intensity	sd	se	ci	Intensity_Change
AR-DAIRY/CHEESE	FERMENTED	1061	NCC 1061	0.428571	0.731925	0.276642	0.676918	42757.14
AR-DAIRY/CHEESE	FERMENTED	1177	NCC 1177	0.142857	0.377964	0.142857	0.349559	14185.71
AR-DAIRY/CHEESE	FERMENTED	1240	NCC 1240	0.528571	1.105613	0.417883	1.022522	52757.14
AR-DAIRY/CHEESE	FERMENTED	156	NCC 156	0.1	0.191485	0.072375	0.177094	9900
AR-DAIRY/CHEESE	FERMENTED	2378	NCC 2378	0.9	0.960655	0.339643	0.803127	89900
AR-DAIRY/CHEESE	FERMENTED	2511	NCC 2511	0.6	0.424264	0.15	0.354694	59900

AR-DAIRY/CHEESE	FERMENTED	2705	NCC 2705	0.111111	0.220479	0.073493	0.169475	11011.11
AR-DAIRY/CHEESE	FERMENTED	2795	NCC 2795	0.666667	0.707107	0.235702	0.54353	66566.67
AR-DAIRY/CHEESE	FERMENTED	283	NCC 283	0.355556	0.474634	0.158211	0.364836	35455.56
AR-DAIRY/CHEESE	FERMENTED	2957	NCC 2957	0.4	0.691014	0.230338	0.53116	39900
AR-DAIRY/CHEESE	FERMENTED	2976	NCC 2976	0.277778	0.440959	0.146986	0.338951	27677.78
AR-DAIRY/CHEESE	FERMENTED	3059	NCC 3059	0.588889	0.747403	0.249134	0.574505	58788.89
AR-DAIRY/CHEESE	FERMENTED	4007	NCC 4007	1.333333	1.198958	0.399653	0.921601	133233.3
AR-DAIRY/CHEESE	FERMENTED	525	NCC 525	0.888889	0.600925	0.200308	0.461912	88788.89
AR-DAIRY/CHEESE	FERMENTED	533	NCC 533	0.633333	0.756637	0.252212	0.581603	63233.33
AR-DAIRY/CHEESE	FERMENTED	660	NCC 660	0.166667	0.353553	0.117851	0.271765	16566.67
AR-DAIRY/CHEESE	UNFERMENTED	UNFERMENTED	UNFERMENTED	0.001	NA	NA	NA	0
AR-FATTY	FERMENTED	1061	NCC 1061	0.671429	0.34983	0.132223	0.323539	34.28571
AR-FATTY	FERMENTED	1177	NCC 1177	0.385714	0.273426	0.103345	0.252877	-22.8571
AR-FATTY	FERMENTED	1240	NCC 1240	0.542857	0.304725	0.115175	0.281823	8.571429
AR-FATTY	FERMENTED	156	NCC 156	0.414286	0.247848	0.093678	0.229221	-17.1429
AR-FATTY	FERMENTED	2378	NCC 2378	0.725	0.349489	0.123563	0.29218	45
AR-FATTY	FERMENTED	2511	NCC 2511	0.55	0.287849	0.10177	0.240648	10
AR-FATTY	FERMENTED	2705	NCC 2705	0.388889	0.34801	0.116003	0.267504	-22.2222
AR-FATTY	FERMENTED	2795	NCC 2795	0.611111	0.220479	0.073493	0.169475	22.22222
AR-FATTY	FERMENTED	283	NCC 283	0.8	0.497494	0.165831	0.382408	60
AR-FATTY	FERMENTED	2957	NCC 2957	0.533333	0.471699	0.157233	0.36258	6.666667
AR-FATTY	FERMENTED	2976	NCC 2976	0.655556	0.415665	0.138555	0.319509	31.11111
AR-FATTY	FERMENTED	3059	NCC 3059	0.555556	0.300463	0.100154	0.230956	11.11111
AR-FATTY	FERMENTED	4007	NCC 4007	1	0.829156	0.276385	0.637346	100
AR-FATTY	FERMENTED	525	NCC 525	0.755556	0.353946	0.117982	0.272067	51.11111
AR-FATTY	FERMENTED	533	NCC 533	0.666667	0.433013	0.144338	0.332843	33.33333
AR-FATTY	FERMENTED	660	NCC 660	0.522222	0.272845	0.090948	0.209727	4.444444
AR-FATTY	UNFERMENTED	UNFERMENTED	UNFERMENTED	0.5	NA	NA	NA	0
AR-GREEN	FERMENTED	1061	NCC 1061	0.557143	0.461364	0.174379	0.426691	-30.3571
AR-GREEN	FERMENTED	1177	NCC 1177	0.757143	0.377964	0.142857	0.349559	-5.35714
AR-GREEN	FERMENTED	1240	NCC 1240	0.7	0.244949	0.092582	0.22654	-12.5

AR-GREEN	FERMENTED	156	NCC 156	0.742857	0.472077	0.178429	0.436599	-7.14286
AR-GREEN	FERMENTED	2378	NCC 2378	0.625	0.249285	0.088135	0.208407	-21.875
AR-GREEN	FERMENTED	2511	NCC 2511	0.8125	0.299702	0.105961	0.250557	1.5625
AR-GREEN	FERMENTED	2705	NCC 2705	0.644444	0.541089	0.180363	0.415918	-19.4444
AR-GREEN	FERMENTED	2795	NCC 2795	0.655556	0.350397	0.116799	0.269339	-18.0556
AR-GREEN	FERMENTED	283	NCC 283	0.588889	0.325747	0.108582	0.250391	-26.3889
AR-GREEN	FERMENTED	2957	NCC 2957	0.466667	0.37081	0.123603	0.28503	-41.6667
AR-GREEN	FERMENTED	2976	NCC 2976	0.655556	0.415665	0.138555	0.319509	-18.0556
AR-GREEN	FERMENTED	3059	NCC 3059	0.688889	0.448454	0.149485	0.344712	-13.8889
AR-GREEN	FERMENTED	4007	NCC 4007	1.255556	1.162015	0.387338	0.893203	56.94444
AR-GREEN	FERMENTED	525	NCC 525	0.644444	0.441902	0.147301	0.339676	-19.4444
AR-GREEN	FERMENTED	533	NCC 533	0.633333	0.53619	0.17873	0.412152	-20.8333
AR-GREEN	FERMENTED	660	NCC 660	0.544444	0.250555	0.083518	0.192594	-31.9444
AR-GREEN	UNFERMENTED	UNFERMENTED	UNFERMENTED	0.8	NA	NA	NA	0
AR-MALTY	FERMENTED	1061	NCC 1061	1.957143	0.793425	0.299887	0.733796	22.32143
AR-MALTY	FERMENTED	1177	NCC 1177	1.242857	0.541163	0.20454	0.500492	-22.3214
AR-MALTY	FERMENTED	1240	NCC 1240	1.828571	0.631702	0.238761	0.584227	14.28571
AR-MALTY	FERMENTED	156	NCC 156	1.871429	0.449868	0.170034	0.416058	16.96429
AR-MALTY	FERMENTED	2378	NCC 2378	1.525	0.634147	0.224205	0.53016	-4.6875
AR-MALTY	FERMENTED	2511	NCC 2511	1.2125	0.619764	0.21912	0.518135	-24.2188
AR-MALTY	FERMENTED	2705	NCC 2705	2.411111	0.49357	0.164523	0.379391	50.69444
AR-MALTY	FERMENTED	2795	NCC 2795	1.644444	0.296273	0.098758	0.227736	2.777778
AR-MALTY	FERMENTED	283	NCC 283	2.166667	0.33541	0.111803	0.257819	35.41667
AR-MALTY	FERMENTED	2957	NCC 2957	1.8	0.864581	0.288194	0.664576	12.5
AR-MALTY	FERMENTED	2976	NCC 2976	1.8	1.00995	0.33665	0.776317	12.5
AR-MALTY	FERMENTED	3059	NCC 3059	1.688889	0.615314	0.205105	0.472972	5.555556
AR-MALTY	FERMENTED	4007	NCC 4007	0.933333	0.784219	0.261406	0.602804	-41.6667
AR-MALTY	FERMENTED	525	NCC 525	1.477778	0.514242	0.171414	0.395281	-7.63889
AR-MALTY	FERMENTED	533	NCC 533	1.344444	0.371184	0.123728	0.285318	-15.9722
AR-MALTY	FERMENTED	660	NCC 660	1.988889	0.682723	0.227574	0.524788	24.30556
AR-MALTY	UNFERMENTED	UNFERMENTED	UNFERMENTED	1.6	NA	NA	NA	0
AR-PEA	FERMENTED	1061	NCC 1061	1.457143	0.824332	0.311568	0.76238	-33.7662
AR-PEA	FERMENTED	1177	NCC 1177	1.842857	0.624118	0.235895	0.577213	-16.2338
AR-PEA	FERMENTED	1240	NCC 1240	1.714286	0.708116	0.267643	0.654898	-22.0779
AR-PEA	FERMENTED	156	NCC 156	1.414286	0.504739	0.190774	0.466806	-35.7143
AR-PEA	FERMENTED	2378	NCC 2378	1.225	0.73046	0.258257	0.61068	-44.3182
AR-PEA	FERMENTED	2511	NCC 2511	1.3125	0.78638	0.278027	0.65743	-40.3409

AR-PEA	FERMENTED	2705	NCC 2705	2.4	0.5	0.166667	0.384334	9.090909
AR-PEA	FERMENTED	2795	NCC 2795	1.566667	0.703562	0.234521	0.540806	-28.7879
AR-PEA	FERMENTED	283	NCC 283	1.455556	0.583333	0.194444	0.44839	-33.8384
AR-PEA	FERMENTED	2957	NCC 2957	1.533333	1.17686	0.392287	0.904615	-30.303
AR-PEA	FERMENTED	2976	NCC 2976	1.8	0.984886	0.328295	0.75705	-18.1818
AR-PEA	FERMENTED	3059	NCC 3059	1.511111	0.776924	0.258975	0.597197	-31.3131
AR-PEA	FERMENTED	4007	NCC 4007	1.255556	0.728202	0.242734	0.559745	-42.9293
AR-PEA	FERMENTED	525	NCC 525	1.344444	0.563718	0.187906	0.433312	-38.8889
AR-PEA	FERMENTED	533	NCC 533	1	0.870345	0.290115	0.669006	-54.5455
AR-PEA	FERMENTED	660	NCC 660	1.655556	0.904311	0.301437	0.695115	-24.7475
AR-PEA	UNFERMENTED	UNFERMENTED	UNFERMENTED	2.2	NA	NA	NA	0

Table S12

Annotation of fatty acids and derivatives by Liquid Chromatography-Electrospray Ionization Mass Spectrometry.This table summarizes the annotation results of a metabolomic analysis focusing on fatty acids and their oxidation products. The average mass-to-charge ratio (Average Mz) serves as the primary identifier for each ionized metabolite, with the Metabolite Name detailing the specific compounds alongside the method of detection and conditions used. Adduct Types indicate the ion species detected, while Reference m/z provides a standard comparison

value. The Formula column lists the chemical makeup of each metabolite, and the Ontology column categorizes them into chemical families or biological functions. Alignment IDs are used for cross-comparison in different runs. The table also includes molecular identifiers with INCHIKEY and SMILES notations. Finally, the MS/MS Spectrum data offer detailed fragmentation patterns.

Alignment ID	Average Rt(min)	Average Mz	Metabolite name	Adduct type	Reference m/z	Formula	Ontology
125N	15.528	277.2171	gamma-Linolenic acid; LC-ESI-QTOF; MS2; CE	[M-H]-	277.21732	C18H30O2	Lineolic acids and derivatives
130N	16.364	279.2322	Chaulmoogric Acid	[M-H]-	279.23294	C18H32O2	Long-chain fatty acids
143N	13.882	293.2105	FA 18:2+O; PlaSMA ID-565	[M-H]-	293.21231	C18H30O3	Oxidized fatty acids
144N	13.446	293.2118	9-KODE; LC-ESI-QIT; MS2; CE	[M-H]-	293.21222	C18H30O3	Lineolic acids and derivatives
145N	13.654	293.2119	13-KODE; LC-ESI-QIT; MS2; CE	[M-H]-	293.21222	C18H30O3	Lineolic acids and derivatives
148N	13.206	295.2254	9-HODE; LC-ESI-QIT; MS2; CE	[M-H2O-H]-	295.22787	C18H32O3	Lineolic acids and derivatives
151N	13.011	295.2273	9-HODE; LC-ESI-QIT; MS2; CE	[M-H]-	295.22787	C18H32O3	Lineolic acids and derivatives
153N	13.435	295.2292	9-HODE; LC-ESI-QIT; MS2; CE	[M-H]-	295.22787	C18H32O3	Lineolic acids and derivatives
157N	15.06	297.2437	FA 18:1+1O; PlaSMA ID-585	[M-H]-	297.24399	C18H34O3	Oxidized fatty acids
168N	10.959	309.2067	13-HpOTrE; LC-ESI-QIT; MS2; CE	[M-H]-	309.20712	C18H30O4	Lineolic acids and derivatives
171N	12.417	311.2209	FA 18:2+2O; PlaSMA ID-645	[M-H]-	311.22189	C18H32O4	Oxidized fatty acids
174N	11.783	311.2238	FA 18:2+2O; PlaSMA ID-650	[M-H]-	311.22269	C18H32O4	Oxidized fatty acids
176N	11.584	311.224	FA 18:2+2O; PlaSMA ID-645	[M-H]-	311.22189	C18H32O4	Oxidized fatty acids
178N	11.109	311.2252	(9Z,12E)-15,16-dihydroxyoctadeca-9,12-dienoic acid	[M-H]-	311.22229	C18H32O4	Oxidized fatty acids
184N	11.645	313.2397	9,10-DiHOME; LC-ESI-QIT; MS2; CE	[M-H]-	313.23843	C18H34O4	Long-chain fatty acids

185N	11.786	313.2402	9,10-DiHOME; LC-ESI-QIT; MS2; CE	[M-H]-	313.23843	C18H34O4	Long-chain fatty acids
206N	9.727	329.2316	FA 18:1+3O	[M-H]-	329.23251	C18H34O5	Oxidized fatty acids
207N	10.737	329.2337	FA 18:1+3O	[M-H]-	329.2312	C18H34O5	Oxidized fatty acids
208N	9.832	329.2339	FA 18:1+3O	[M-H]-	329.23251	C18H34O5	Oxidized fatty acids
209N	9.258	329.234	FA 18:1+3O	[M-H]-	329.2312	C18H34O5	Oxidized fatty acids
210N	10.255	329.2344	FA 18:1+3O	[M-H]-	329.23251	C18H34O5	Oxidized fatty acids
211N	9.985	329.2351	FA 18:1+3O	[M-H]-	329.23251	C18H34O5	Oxidized fatty acids

Table S12 (continuation)

Alignment ID	INCHIKEY	SMILES	MS/MS spectrum
125N	VZCCETWTMQHEPK-QNEBEIHSSA-N	CCCCC\C=C/C\C=C/C\C=C/CCCCC(O)=O	71.01524:4
130N	XMVQWNRDPAAMJB-UHFFFAOYSA-N	O=C(O)CCCCCCCCCCCCC1C=CCC1	279.22210:4
143N	HKSDVVJONLXYKL-UHFFFAOYNA-N	O=C(O)CCCCCCC=CCC=CCC1OC1(CC)	95.04718:12 293.20244:12
144N	LUZSWWYKKLTDHU-SIGMCMEVSA-N	CCCCC\C=C\C=C\C(=O)CCCCCCCC(O)=O	57.03294:2 59.01506:2 71.01086:2 96.9527 [illegible]
145N	JHXAZBBVQSRKJR-KDFHGORWSA-N	CCCCCC(=O)\C=C\C=CCCCCCCCC(O)=O	113.09404:13
148N	NPDSHTNEKLQQIJ-ZJHFMPGANA-N	CCCCC\C=C/C=C/C(O)CCCCCCCC(O)=O	171.09974:19
151N	NPDSHTNEKLQQIJ-ZJHFMPGANA-N	CCCCC\C=C/C=C/C(O)CCCCCCCC(O)=O	277.21180:6
153N	NPDSHTNEKLQQIJ-ZJHFMPGANA-N	CCCCC\C=C/C=C/C(O)CCCCCCCC(O)=O	95.04984:4 171.09861:18 277.20909:7
157N	IMYZYCNQZDBZBQ-UHFFFAOYNA-N	O=C(O)CCCCCCCC1OC1(CCCCCCCC)	297.24355:8
168N	UYQGVDXDXBAABN-SKEAHQKTNA-N	CC\C=C/CC(OO)\C=C\C=CCCCCCCCC(O)=O	152.08330:5
171N	JGUNZIWGNMQSBM-UHFFFAOYNA-N	O=C(O)CCCCCCCC(OO)C=CC=CCCCCC	99.07939:4
174N	JGUNZIWGNMQSBM-UHFFFAOYNA-N	O=C(O)CCCCCCCC(OO)C=CC=CCCCCC	113.09774:5 125.09659:6 183.00950:6
176N	JGUNZIWGNMQSBM-UHFFFAOYNA-N	O=C(O)CCCCCCCC(OO)C=CC=CCCCCC	99.07873:8
178N	LKLLJYJTYPVCID-JVVXYUKTSA-N	O=C(O)CCCCCCCC=CCC=CCC(O)C(O)CC	183.13593:5
184N	XEBKSQSGNGRGDW-JXMROGBWNA-N	CCCCC\C=C\CC(O)C(O)CCCCCCCC(O)=O	99.08020:642
185N	XEBKSQSGNGRGDW-JXMROGBWNA-N	CCCCC\C=C\CC(O)C(O)CCCCCCCC(O)=O	58.00437:11 99.07911:9 125.09342:9 127.1 [illegible]
206N	MDIUMSLCYIJBQC-UHFFFAOYNA-N	O=C(O)CCCCCCCC(O)C=CC(O)C(O)CCCCC	58.00386:8 99.07914:9 129.08945:4 169.11 [illegible]
207N	MDIUMSLCYIJBQC-UHFFFAOYNA-N	O=C(O)CCCCCCCC(O)C=CC(O)C(O)CCCCC	57.03307:6 58.00472:18 59.00872:6 73.024 [illegible]
208N	MDIUMSLCYIJBQC-UHFFFAOYNA-N	O=C(O)CCCCCCCC(O)C=CC(O)C(O)CCCCC	99.07978:14
209N	MDIUMSLCYIJBQC-UHFFFAOYNA-N	O=C(O)CCCCCCCC(O)C=CC(O)C(O)CCCCC	99.08056:30 125.09537:21 139.11158:26 17 [illegible]
210N	MDIUMSLCYIJBQC-UHFFFAOYNA-N	O=C(O)CCCCCCCC(O)C=CC(O)C(O)CCCCC	99.07888:14
211N	MDIUMSLCYIJBQC-UHFFFAOYNA-N	O=C(O)CCCCCCCC(O)C=CC(O)C(O)CCCCC	59.01259:28 73.02878:4 99.07960:30 127.0 [illegible]

Table S13

This table report the sensory data from round 3 depicted in Figure 5, for the aroma attributes specifically. For each fermented sample tested and for each attribute evaluated this table report the average intensity “intensity”, the standard deviation, the standard error of the mean (se) and the related confidence intervals.

Attribute	TREATMENT	NCC	Intensity	sd	se	ci
AR-DAIRY/CHEESE	FERMENTED	NCC2378	1.083333	1.934339	0.789691	2.029964
AR-DAIRY/CHEESE	FERMENTED	NCC2705	0.8	0.916515	0.374166	0.961824
AR-DAIRY/CHEESE	FERMENTED	NCC3059	0.385714	0.456175	0.172418	0.421891
AR-DAIRY/CHEESE	FERMENTED	NCC4007	0.166667	0.258199	0.105409	0.270963
AR-DAIRY/CHEESE	FERMENTED	NCC533	0.666667	0.516398	0.210819	0.541926
AR-DAIRY/CHEESE	FERMENTED	NCC660	0.214286	0.267261	0.101015	0.247175
AR-DAIRY/CHEESE	UNFERMENTED	UNFERMENTED	0	NA	NA	NA
AR-FATTY	FERMENTED	NCC2378	0.433333	0.216025	0.088192	0.226704
AR-FATTY	FERMENTED	NCC2705	0.616667	0.231661	0.094575	0.243113
AR-FATTY	FERMENTED	NCC3059	0.728571	0.256348	0.09689	0.237082
AR-FATTY	FERMENTED	NCC4007	0.35	0.197484	0.080623	0.207247
AR-FATTY	FERMENTED	NCC533	0.5	0.316228	0.129099	0.331861
AR-FATTY	FERMENTED	NCC660	0.642857	0.35051	0.13248	0.324168
AR-FATTY	UNFERMENTED	UNFERMENTED	0.5	NA	NA	NA
AR-GREEN	FERMENTED	NCC2378	0.55	0.361939	0.147761	0.379832
AR-GREEN	FERMENTED	NCC2705	0.85	0.441588	0.180278	0.463418
AR-GREEN	FERMENTED	NCC3059	0.528571	0.411154	0.155402	0.380254
AR-GREEN	FERMENTED	NCC4007	0.7	0.572713	0.233809	0.601025
AR-GREEN	FERMENTED	NCC533	0.6	0.178885	0.07303	0.187729
AR-GREEN	FERMENTED	NCC660	0.214286	0.177281	0.067006	0.163958
AR-GREEN	UNFERMENTED	UNFERMENTED	0.8	NA	NA	NA
AR-MALTY	FERMENTED	NCC2378	1.7	0.167332	0.068313	0.175604
AR-MALTY	FERMENTED	NCC2705	1.583333	0.491596	0.200693	0.515898
AR-MALTY	FERMENTED	NCC3059	1.257143	0.869592	0.328675	0.804239
AR-MALTY	FERMENTED	NCC4007	1.7	0.268328	0.109545	0.281593
AR-MALTY	FERMENTED	NCC533	1.616667	0.204124	0.083333	0.214215
AR-MALTY	FERMENTED	NCC660	1.185714	0.318479	0.120374	0.294544
AR-MALTY	UNFERMENTED	UNFERMENTED	1.6	NA	NA	NA
AR-PEA	FERMENTED	NCC2378	1.916667	0.376386	0.153659	0.394993
AR-PEA	FERMENTED	NCC2705	1.3	0.596657	0.243584	0.626153
AR-PEA	FERMENTED	NCC3059	1.714286	0.34365	0.129887	0.317823
AR-PEA	FERMENTED	NCC4007	1.45	0.889382	0.363089	0.933349
AR-PEA	FERMENTED	NCC533	2	0.632456	0.258199	0.663721
AR-PEA	FERMENTED	NCC660	1.571429	0.464451	0.175546	0.429545

AR-PEA	UNFERMENTED	UNFERMENTED	2.2	NA	NA	NA

Table S14

This table report the sensory data from round 3 depicted in Figure 5, for the taste attributes specifically. For each fermented sample tested and for each attribute evaluated this table report the average intensity "intensity", the standard deviation, the standard error of the mean (se) and the related confidence intervals.

Attribute	TREATMENT	NCC	Intensity	sd	se	ci
TS-ASTRINGENT	FERMENTED	NCC2378	2.7	0.4	0.163299	0.419774
TS-ASTRINGENT	FERMENTED	NCC2705	2.283333	1.020621	0.416667	1.071076
TS-ASTRINGENT	FERMENTED	NCC3059	2.571429	0.449868	0.170034	0.416058
TS-ASTRINGENT	FERMENTED	NCC4007	2.416667	0.73598	0.300463	0.772364
TS-ASTRINGENT	FERMENTED	NCC533	2.3	0.565685	0.23094	0.59365
TS-ASTRINGENT	FERMENTED	NCC660	2.214286	0.809174	0.305839	0.748361
TS-ASTRINGENT	UNFERMENTED	UNFERMENTED	3	NA	NA	NA
TS-BITTER	FERMENTED	NCC2378	2.15	0.281069	0.114746	0.294964
TS-BITTER	FERMENTED	NCC2705	1.616667	0.649359	0.2651	0.68146
TS-BITTER	FERMENTED	NCC3059	1.728571	0.644759	0.243696	0.596303
TS-BITTER	FERMENTED	NCC4007	2.4	0.451664	0.184391	0.473992
TS-BITTER	FERMENTED	NCC533	1.15	0.423084	0.172723	0.443999
TS-BITTER	FERMENTED	NCC660	1.985714	0.84937	0.321032	0.785536
TS-BITTER	UNFERMENTED	UNFERMENTED	2.5	NA	NA	NA
TS-SALTY	FERMENTED	NCC2378	0.733333	0.136626	0.055777	0.14338
TS-SALTY	FERMENTED	NCC2705	0.75	0.234521	0.095743	0.246114
TS-SALTY	FERMENTED	NCC3059	0.828571	0.138013	0.052164	0.127641
TS-SALTY	FERMENTED	NCC4007	0.65	0.197484	0.080623	0.207247
TS-SALTY	FERMENTED	NCC533	1.016667	0.30605	0.124944	0.32118
TS-SALTY	FERMENTED	NCC660	0.814286	0.338765	0.128041	0.313306
TS-SALTY	UNFERMENTED	UNFERMENTED	0.7	NA	NA	NA
TS-SOUR	FERMENTED	NCC2378	0.616667	0.381663	0.155813	0.400531
TS-SOUR	FERMENTED	NCC2705	1.133333	0.989276	0.40387	1.038181
TS-SOUR	FERMENTED	NCC3059	0.742857	0.181265	0.068512	0.167643
TS-SOUR	FERMENTED	NCC4007	0.7	0.675278	0.275681	0.708661
TS-SOUR	FERMENTED	NCC533	0.7	0.414729	0.169312	0.435231
TS-SOUR	FERMENTED	NCC660	0.485714	0.318479	0.120374	0.294544
TS-SOUR	UNFERMENTED	UNFERMENTED	0.6	NA	NA	NA
TS-SWEET	FERMENTED	NCC2378	0.3	0.368782	0.150555	0.387013
TS-SWEET	FERMENTED	NCC2705	0.533333	0.287518	0.117379	0.301732
TS-SWEET	FERMENTED	NCC3059	0.714286	0.157359	0.059476	0.145533
TS-SWEET	FERMENTED	NCC4007	0.716667	0.479236	0.195647	0.502927
TS-SWEET	FERMENTED	NCC533	0.466667	0.36697	0.149815	0.385111

TS-SWEET	FERMENTED	NCC660	0.614286	0.313202	0.118379	0.289663
TS-SWEET	UNFERMENTED	UNFERMENTED	0.7	NA	NA	NA
TS-UMAMI	FERMENTED	NCC2378	0.716667	0.160208	0.065405	0.168128
TS-UMAMI	FERMENTED	NCC2705	0.883333	0.271416	0.110805	0.284834
TS-UMAMI	FERMENTED	NCC3059	0.671429	0.095119	0.035952	0.08797
TS-UMAMI	FERMENTED	NCC4007	0.583333	0.040825	0.016667	0.042843
TS-UMAMI	FERMENTED	NCC533	1.733333	0.436654	0.178263	0.45824
TS-UMAMI	FERMENTED	NCC660	1.085714	0.157359	0.059476	0.145533
TS-UMAMI	UNFERMENTED	UNFERMENTED	0.6	NA	NA	NA

Table S15

This table presents the calculated Odor Activity Values (OAV), Odor threshold and concentration (µg/kg) for each aroma compound identified and quantified in the fermented samples from Round 3. It includes data on the NCC strain used, the fermentation time for each sample, and the associated standard deviations (sd). The 0h samples serve as control samples, while the 48h samples represent the fermented products. Analyses were conducted in triplicate for the fermented samples, and their standard deviations are accordingly reported. Unfermented controls were analyzed using one technical replicate, for this reason sd is set to NA.

Aroma Compound	TIME	NCC	Odor treshold (µg/kg)	Concentration (µg/kg)	OAV	sd
2,3-butanedione	0H	NCC2378	0.9	347.69	386.32	NA
2,3-butanedione	0H	NCC2705	0.9	316.48	351.64	NA
2,3-butanedione	0H	NCC3059	0.9	297.68	330.75	NA
2,3-butanedione	0H	NCC4007	0.9	328.56	365.07	NA
2,3-butanedione	0H	NCC533	0.9	277.61	308.46	NA
2,3-butanedione	0H	NCC660	0.9	332.30	369.22	NA
2,3-butanedione	48H	NCC2378	0.9	348.79	387.55	9.72
2,3-butanedione	48H	NCC2705	0.9	303.23	336.93	4.82
2,3-butanedione	48H	NCC3059	0.9	341.24	379.16	42.24
2,3-butanedione	48H	NCC4007	0.9	291.95	324.38	8.24
2,3-butanedione	48H	NCC533	0.9	947.85	1053.2	26.06
2,3-butanedione	48H	NCC660	0.9	165.42	183.80	13.82
2,3 octanedione	0H	NCC2378	29	307.75	10.61	NA
2,3 octanedione	0H	NCC2705	29	396.99	13.69	NA
2,3 octanedione	0H	NCC3059	29	325.43	11.22	NA
2,3 octanedione	0H	NCC4007	29	337.76	11.65	NA
2,3 octanedione	0H	NCC533	29	394.17	13.59	NA
2,3 octanedione	0H	NCC660	29	346.47	11.95	NA
2,3 octanedione	48H	NCC2378	29	311.76	10.75	0.65
2,3 octanedione	48H	NCC2705	29	348.88	12.03	0.57

2,3 octanedione	48H	NCC3059	29	336.08	11.59	1.43
2,3 octanedione	48H	NCC4007	29	364.47	12.57	1.06
2,3 octanedione	48H	NCC533	29	365.44	12.60	2.07
2,3 octanedione	48H	NCC660	29	343.32	11.84	0.18
3/2-methylbutanal	0H	NCC2378	0.5/1.5	409.36	818.72	NA
3/2-methylbutanal	0H	NCC2705	0.5/1.5	408.39	816.78	NA
3/2-methylbutanal	0H	NCC3059	0.5/1.5	368.66	737.32	NA
3/2-methylbutanal	0H	NCC4007	0.5/1.5	418.26	836.52	NA
3/2-methylbutanal	0H	NCC533	0.5/1.5	401.80	803.60	NA
3/2-methylbutanal	0H	NCC660	0.5/1.5	358.13	716.26	NA
3/2-methylbutanal	48H	NCC2378	0.5/1.5	349.29	698.58	18.32
3/2-methylbutanal	48H	NCC2705	0.5/1.5	345.42	690.83	37.67
3/2-methylbutanal	48H	NCC3059	0.5/1.5	434.71	869.43	78.06
3/2-methylbutanal	48H	NCC4007	0.5/1.5	380.90	761.81	46.44
3/2-methylbutanal	48H	NCC533	0.5/1.5	324.96	649.91	42.25
3/2-methylbutanal	48H	NCC660	0.5/1.5	214.95	429.90	33.50
Acetaldehyde	0H	NCC2378	16	87592.11	5474.51	NA
Acetaldehyde	0H	NCC2705	16	88087.78	5505.49	NA
Acetaldehyde	0H	NCC3059	16	80436.88	5027.31	NA
Acetaldehyde	0H	NCC4007	16	85030.51	5314.41	NA
Acetaldehyde	0H	NCC533	16	85368.21	5335.51	NA
Acetaldehyde	0H	NCC660	16	81640.83	5102.55	NA
Acetaldehyde	48H	NCC2378	16	84176.74	5261.0	362.79
Acetaldehyde	48H	NCC2705	16	57681.25	3605.1	402.78
Acetaldehyde	48H	NCC3059	16	11773.67	735.85	59.96
Acetaldehyde	48H	NCC4007	16	80453.06	5028.3	696.53
Acetaldehyde	48H	NCC533	16	17595.15	1099.7	56.33
Acetaldehyde	48H	NCC660	16	11986.15	749.13	77.39
Acetic acid	0H	NCC2378	5600	38052.57	6.80	NA
Acetic acid	0H	NCC2705	5600	36103.14	6.45	NA
Acetic acid	0H	NCC3059	5600	34160.60	6.10	NA
Acetic acid	0H	NCC4007	5600	39561.60	7.06	NA
Acetic acid	0H	NCC533	5600	38427.55	6.86	NA
Acetic acid	0H	NCC660	5600	34898.96	6.23	NA
Acetic acid	48H	NCC2378	5600	35088.36	6.27	1.17
Acetic acid	48H	NCC2705	5600	34917.14	6.24	0.76
Acetic acid	48H	NCC3059	5600	40816.87	7.29	0.85
Acetic acid	48H	NCC4007	5600	42412.36	7.57	0.55
Acetic acid	48H	NCC533	5600	31374.46	5.60	0.70
Acetic acid	48H	NCC660	5600	25776.98	4.60	0.16
Acetoin	0H	NCC2378	590	41002.85	69.50	NA
Acetoin	0H	NCC2705	590	28206.19	47.81	NA
Acetoin	0H	NCC3059	590	32976.20	55.89	NA
Acetoin	0H	NCC4007	590	26002.92	44.07	NA
Acetoin	0H	NCC533	590	27287.67	46.25	NA
Acetoin	0H	NCC660	590	25352.79	42.97	NA

Acetoin	48H	NCC2378	590	89413.50	151.55	32.09
Acetoin	48H	NCC2705	590	41850.05	70.93	2.31
Acetoin	48H	NCC3059	590	12432.11	21.07	3.13
Acetoin	48H	NCC4007	590	73240.48	124.14	8.86
Acetoin	48H	NCC533	590	627775.34	1064.03	31.09
Acetoin	48H	NCC660	590	9882.91	16.75	2.45
Benzaldehyde	0H	NCC2378	150	6336.38	42.24	NA
Benzaldehyde	0H	NCC2705	150	6517.94	43.45	NA
Benzaldehyde	0H	NCC3059	150	5611.00	37.41	NA
Benzaldehyde	0H	NCC4007	150	5897.72	39.32	NA
Benzaldehyde	0H	NCC533	150	6312.04	42.08	NA
Benzaldehyde	0H	NCC660	150	5778.39	38.52	NA
Benzaldehyde	48H	NCC2378	150	4851.09	32.34	5.16
Benzaldehyde	48H	NCC2705	150	178.34	1.19	0.32
Benzaldehyde	48H	NCC3059	150	182.20	1.21	0.16
Benzaldehyde	48H	NCC4007	150	1851.59	12.34	1.31
Benzaldehyde	48H	NCC533	150	151.51	1.01	0.14
Benzaldehyde	48H	NCC660	150	175.16	1.17	0.16
E-2-Octenal	0H	NCC2378	1.7	380.29	223.70	NA
E-2-Octenal	0H	NCC2705	1.7	390.42	229.66	NA
E-2-Octenal	0H	NCC3059	1.7	392.21	230.71	NA
E-2-Octenal	0H	NCC4007	1.7	397.41	233.77	NA
E-2-Octenal	0H	NCC533	1.7	380.98	224.10	NA
E-2-Octenal	0H	NCC660	1.7	384.57	226.22	NA
E-2-Octenal	48H	NCC2378	1.7	404.60	238.00	27.26
E-2-Octenal	48H	NCC2705	1.7	421.49	247.93	9.52
E-2-Octenal	48H	NCC3059	1.7	381.98	224.69	10.91
E-2-Octenal	48H	NCC4007	1.7	341.43	200.84	6.97
E-2-Octenal	48H	NCC533	1.7	62.42	36.72	2.20
E-2-Octenal	48H	NCC660	1.7	1.37	0.80	0.15
E-2-Undecenal	0H	NCC2378	0.78	974.51	1249.37	NA
E-2-Undecenal	0H	NCC2705	0.78	947.69	1214.99	NA
E-2-Undecenal	0H	NCC3059	0.78	915.89	1174.22	NA
E-2-Undecenal	0H	NCC4007	0.78	942.19	1207.94	NA
E-2-Undecenal	0H	NCC533	0.78	909.78	1166.39	NA
E-2-Undecenal	0H	NCC660	0.78	876.37	1123.55	NA
E-2-Undecenal	48H	NCC2378	0.78	847.92	1087.07	239.56
E-2-Undecenal	48H	NCC2705	0.78	734.57	941.75	36.40
E-2-Undecenal	48H	NCC3059	0.78	881.95	1130.71	58.80
E-2-Undecenal	48H	NCC4007	0.78	801.61	1027.71	94.94
E-2-Undecenal	48H	NCC533	0.78	630.25	808.02	20.24
E-2-Undecenal	48H	NCC660	0.78	583.85	748.53	23.55
E,E-2,4-Decadienal	0H	NCC2378	0.027	816.85	30253.77	NA
E,E-2,4-Decadienal	0H	NCC2705	0.027	831.47	30795.04	NA
E,E-2,4-Decadienal	0H	NCC3059	0.027	851.20	31525.85	NA
E,E-2,4-Decadienal	0H	NCC4007	0.027	871.84	32290.24	NA

E,E-2,4-Decadienal	0H	NCC533	0.027	862.33	31938.08	NA
E,E-2,4-Decadienal	0H	NCC660	0.027	819.71	30359.80	NA
E,E-2,4-Decadienal	48H	NCC2378	0.027	856.51	31722.56	4416.81
E,E-2,4-Decadienal	48H	NCC2705	0.027	705.98	26147.49	1161.48
E,E-2,4-Decadienal	48H	NCC3059	0.027	880.15	32598.22	4594.22
E,E-2,4-Decadienal	48H	NCC4007	0.027	724.86	26846.61	2231.98
E,E-2,4-Decadienal	48H	NCC533	0.027	605.43	22423.19	622.16
E,E-2,4-Decadienal	48H	NCC660	0.027	526.45	19498.30	1053.21
E,E-2,4-Nonadienal	0H	NCC2378	0.046	615.98	13390.83	NA
E,E-2,4-Nonadienal	0H	NCC2705	0.046	611.94	13303.06	NA
E,E-2,4-Nonadienal	0H	NCC3059	0.046	551.86	11996.90	NA
E,E-2,4-Nonadienal	0H	NCC4007	0.046	596.85	12975.07	NA
E,E-2,4-Nonadienal	0H	NCC533	0.046	597.26	12984.01	NA
E,E-2,4-Nonadienal	0H	NCC660	0.046	554.70	12058.63	NA
E,E-2,4-Nonadienal	48H	NCC2378	0.046	571.17	12416.66	1620.27
E,E-2,4-Nonadienal	48H	NCC2705	0.046	536.04	11653.05	455.27
E,E-2,4-Nonadienal	48H	NCC3059	0.046	578.20	12569.54	664.05
E,E-2,4-Nonadienal	48H	NCC4007	0.046	602.42	13096.12	482.62
E,E-2,4-Nonadienal	48H	NCC533	0.046	544.36	11833.84	848.43
E,E-2,4-Nonadienal	48H	NCC660	0.046	436.51	9489.28	375.56
E,E-3,5-Octadien-2-on	0H	NCC2378	27	12.43	0.46	NA
E,E-3,5-Octadien-2-on	0H	NCC2705	27	12.00	0.44	NA
E,E-3,5-Octadien-2-on	0H	NCC3059	27	16.18	0.60	NA
E,E-3,5-Octadien-2-on	0H	NCC4007	27	16.97	0.63	NA
E,E-3,5-Octadien-2-on	0H	NCC533	27	12.22	0.45	NA
E,E-3,5-Octadien-2-on	0H	NCC660	27	12.88	0.48	NA
E,E-3,5-Octadien-2-on	48H	NCC2378	27	15.51	0.57	0.16
E,E-3,5-Octadien-2-on	48H	NCC2705	27	13.32	0.49	0.16
E,E-3,5-Octadien-2-on	48H	NCC3059	27	13.97	0.52	0.05
E,E-3,5-Octadien-2-on	48H	NCC4007	27	14.40	0.53	0.11

E,E-3,5-Octadien-2-on	48H	NCC533	27	6.39	0.24	0.03
E,E-3,5-Octadien-2-on	48H	NCC660	27	2.65	0.10	0.02
heptanal	0H	NCC2378	6.1	463.43	75.97	NA
heptanal	0H	NCC2705	6.1	431.69	70.77	NA
heptanal	0H	NCC3059	6.1	461.20	75.61	NA
heptanal	0H	NCC4007	6.1	457.39	74.98	NA
heptanal	0H	NCC533	6.1	428.44	70.24	NA
heptanal	0H	NCC660	6.1	434.06	71.16	NA
heptanal	48H	NCC2378	6.1	261.11	42.81	5.97
heptanal	48H	NCC2705	6.1	337.81	55.38	3.03
heptanal	48H	NCC3059	6.1	424.49	69.59	4.93
heptanal	48H	NCC4007	6.1	381.03	62.46	6.40
heptanal	48H	NCC533	6.1	278.03	45.58	2.44
heptanal	48H	NCC660	6.1	168.66	27.65	7.26
Hexanal	0H	NCC2378	2.4	1352.72	563.63	NA
Hexanal	0H	NCC2705	2.4	1366.23	569.26	NA
Hexanal	0H	NCC3059	2.4	1275.60	531.50	NA
Hexanal	0H	NCC4007	2.4	1334.73	556.14	NA
Hexanal	0H	NCC533	2.4	1268.07	528.36	NA
Hexanal	0H	NCC660	2.4	1251.66	521.53	NA
Hexanal	48H	NCC2378	2.4	547.71	228.21	26.09
Hexanal	48H	NCC2705	2.4	438.48	182.70	11.13
Hexanal	48H	NCC3059	2.4	322.95	134.56	11.28
Hexanal	48H	NCC4007	2.4	597.46	248.94	30.24
Hexanal	48H	NCC533	2.4	250.23	104.26	5.68
Hexanal	48H	NCC660	2.4	63.66	26.53	2.49
Hexanoic acid	0H	NCC2378	5.4	0.32	0.06	NA
Hexanoic acid	0H	NCC2705	5.4	0.32	0.06	NA
Hexanoic acid	0H	NCC3059	5.4	0.26	0.05	NA
Hexanoic acid	0H	NCC4007	5.4	0.30	0.06	NA
Hexanoic acid	0H	NCC533	5.4	0.31	0.06	NA
Hexanoic acid	0H	NCC660	5.4	0.30	0.06	NA
Hexanoic acid	48H	NCC2378	5.4	0.32	0.06	0.008
Hexanoic acid	48H	NCC2705	5.4	0.26	0.05	0.006
Hexanoic acid	48H	NCC3059	5.4	0.29	0.05	0.006
Hexanoic acid	48H	NCC4007	5.4	0.50	0.09	0.002
Hexanoic acid	48H	NCC533	5.4	0.27	0.05	0.005
Hexanoic acid	48H	NCC660	5.4	0.14	0.03	0.005
phenylacetaldehyde	0H	NCC2378	5.2	1051.60	202.23	NA
phenylacetaldehyde	0H	NCC2705	5.2	1044.90	200.94	NA
phenylacetaldehyde	0H	NCC3059	5.2	1044.42	200.85	NA
phenylacetaldehyde	0H	NCC4007	5.2	1084.77	208.61	NA
phenylacetaldehyde	0H	NCC533	5.2	1088.65	209.36	NA
phenylacetaldehyde	0H	NCC660	5.2	999.66	192.24	NA

phenylacetaldehyde	48H	NCC2378	5.2	844.15	162.34	9.42
phenylacetaldehyde	48H	NCC2705	5.2	771.42	148.35	4.82
phenylacetaldehyde	48H	NCC3059	5.2	886.20	170.42	9.35
phenylacetaldehyde	48H	NCC4007	5.2	874.27	168.13	2.32
phenylacetaldehyde	48H	NCC533	5.2	1223.13	235.22	21.64
phenylacetaldehyde	48H	NCC660	5.2	809.94	155.76	7.12
Phenylacetic acid	0H	NCC2378	26	40.98	1.58	NA
Phenylacetic acid	0H	NCC2705	26	41.16	1.58	NA
Phenylacetic acid	0H	NCC3059	26	51.26	1.97	NA
Phenylacetic acid	0H	NCC4007	26	32.44	1.25	NA
Phenylacetic acid	0H	NCC533	26	33.35	1.28	NA
Phenylacetic acid	0H	NCC660	26	51.14	1.97	NA
Phenylacetic acid	48H	NCC2378	26	55.31	2.13	0.42
Phenylacetic acid	48H	NCC2705	26	55.45	2.13	0.58
Phenylacetic acid	48H	NCC3059	26	68.52	2.64	0.52
Phenylacetic acid	48H	NCC4007	26	44.28	1.70	0.32
Phenylacetic acid	48H	NCC533	26	324.08	12.46	1.64
Phenylacetic acid	48H	NCC660	26	50.91	1.96	0.06
Vanillin	0H	NCC2378	53	14.27	0.27	NA
Vanillin	0H	NCC2705	53	15.40	0.29	NA
Vanillin	0H	NCC3059	53	19.60	0.37	NA
Vanillin	0H	NCC4007	53	18.33	0.35	NA
Vanillin	0H	NCC533	53	18.23	0.34	NA
Vanillin	0H	NCC660	53	19.94	0.38	NA
Vanillin	48H	NCC2378	53	11.06	0.21	0.03
Vanillin	48H	NCC2705	53	13.60	0.26	0.08
Vanillin	48H	NCC3059	53	8.93	0.17	0.04
Vanillin	48H	NCC4007	53	12.64	0.24	0.02
Vanillin	48H	NCC533	53	9.12	0.17	0.03
Vanillin	48H	NCC660	53	4.93	0.09	0.01
